# START adolescents: study protocol of a randomised controlled trial to investigate the efficacy of a low-threshold group treatment programme in traumatised adolescent refugees

**DOI:** 10.1136/bmjopen-2021-057968

**Published:** 2021-12-28

**Authors:** Esther Sobanski, Florian Hammerle, Andrea Dixius, Eva Möhler, Susanne Koudela-Hamila, Ulrich Ebner-Priemer, Christian J Merz, Tina In-Albon, Brigitte Pollitt, Hanna Christiansen, David Kolar, Susanne Ocker, Nicole Fischer, Ina Burghaus, Michael Huss

**Affiliations:** 1 Department of Child and Adolescent Psychiatry and Psychotherapy, University Medical Centre of the Johannes Gutenberg University Mainz, Mainz, Germany; 2 Department of Psychiatry and Psychotherapy, Central Institute of Mental Health, Medical Faculty Mannheim, University Heidelberg, Mannheim, Germany; 3 Department of Child and Adolescent Psychiatry, SHG Saarland Hospital Group, Idar-Oberstein, Germany; 4 Department of Child and Adolescent Psychiatry, SHG Saarland Hospital Group, Klein-Blittersdorf, Germany; 5 Department of Applied Psychology, Mental mHealth Lab, Karlsruhe Institute of Technology, Karlsruhe, Germany; 6 Department of Cognitive Psychology, Institute of Cognitive Neuroscience, Ruhr University Bochum, Bochum, Germany; 7 Department of Child and Adolescent Psychology and Psychotherapy, University of Koblenz Landau - Campus Landau, Landau, Germany; 8 Department of Child and Adolescent Psychiatry and Psychotherapy, Johanniter Clinics, Neuwied, Germany; 9 Department of Psychology, Clinical Child and Adolescent Psychology, University of Marburg, Marburg, Germany; 10 Coordination Centre for Clinical Trials, University of Heidelberg, Heidelberg, Germany

**Keywords:** child & adolescent psychiatry, protocols & guidelines, psychiatry

## Abstract

**Introduction:**

No evaluated therapeutic approaches, that can efficiently be established in routine mental healthcare, are currently available for traumatised adolescent refugees in Germany. This study evaluates the efficacy of the Stress-Traumasymptoms-Arousal-Regulation-Treatment (START) programme to reduce trauma-related symptoms and psychological distress in traumatised adolescent refugees based in Germany.

**Methods and analysis:**

This randomised, waiting-list-controlled, multicentre trial with a 12-week follow-up will include 174 refugee minors with partial or full post-traumatic stress disorder who are fluent in either Arabic, Dari, English, German or Somali. Eligible refugee minors will be randomised to the START or waiting-list control groups. The manualised 8-week START programme is based on techniques of dialectical behaviour therapy (DBT), fosters adaptive coping with emotional distress and traumatic symptoms and comprises eight therapy modules and a booster session. Study assessments are planned at baseline, post-treatment (ie, after programme participation or waiting time), booster session at week 12 or 12-week waiting time, and at the 12-week follow-up. Primary and coprimary outcomes are changes in psychological distress and traumatic symptoms at post-treatment and will be analysed as response variables in linear mixed regression models. Secondary outcomes are changes in further trauma-related and other psychopathological symptoms, emotion regulation and intermediate effects of the programme at follow-up. We will also assess effects of the programme with ecological momentary assessments and on neuroendocrine stress parameters using hair cortisol.

**Ethics and dissemination:**

This study has been approved by the lead ethics committee of Rhineland-Palatinate and the ethics committees of participating sites. The study results will be disseminated through peer-reviewed publications and scientific conferences.

**Trial registration number:**

DRKS00020771.

**Strengths and limitations of this study**

- Randomised waiting-list controlled multicentre trial with follow-up.
- Manualised DBT-based psychotherapeutic programme with patient material available in Arabic, Dari, English, German and Somali.
- Multimodal assessment approach of intervention effects on symptoms, functioning and daily living outcomes by psychometric evaluations, electronic ambulatory assessments (e-diaries) and neuroendocrine stress parameters.
- Restriction of study participation to refugee minors with language skills in Arabic, Dari, English, German and Somali.

## Background and rationale

Among the forcibly displaced persons worldwide there is a high number of children and adolescents under the age of 18 years.^1^ At the peak of the refugee influx in Germany in 2016, 261 386 of the 745 545 asylum applications recorded in total were filed from refugees under the age of 18 of which 49 786 applications were filed from unaccompanied refugee minors in the care of youth welfare services and without the company of parents or persons with the right of custody. The high percentage of asylum applications filed by refugee minors has remained stable since then though due to political changes the total number of refugees entering Germany has steadily decreased.^2 3^ Of all refugees, children and adolescents, and especially unaccompanied refugee minors, are the most vulnerable group. They are at particular risk of traumatisation before, during and after their flight as well as for mental health problems.^4–6^ Children and adolescents seeking asylum in Germany report an average of eight potentially traumatic experiences, with 98% of unaccompanied refugee minors reporting traumatic events.^5–7^ They are exposed to stress due to insecure legal status and adaptation to new environments, cultural demands and language, the loss of stability provided by their cultural background of origin as well as provided by their families and caregivers if unaccompanied.^8^ In addition to their often prevalent traumatisation, they are also confronted with age-related developmental tasks like peer group integration, identity formation, education, professional training and finding their role in the host society. Cross-sectional studies and reviews assessing mental health problems in refugee minors report prevalence rates of 17%–71% for post-traumatic stress disorder (PTSD), 12%–44% for depressive disorders, 18%–38% for anxiety disorders and 33%–72% for behaviour problems.^9 10^ Longitudinal studies suggest a high risk of chronic mental health problems and indicate that baseline symptom severity, sex, being unaccompanied and post-migration factors like asylum status and access to mental healthcare are predictors of future mental health status.^6 11^ Studies evaluating trauma-related therapy programmes in refugee minors have mostly reported significant effects on trauma and/or additional mental health symptoms. However, conclusive interpretation of the findings is limited by heterogeneous therapy approaches, lack of manualised programmes and methodological shortcomings like lack of power analyses, randomisation and control groups, small sample sizes or heterogeneous study settings such as clinical, school or community settings in high-income countries, but also refugee camps in war regions.^12–17^ Evidence from rigorous methodological approaches like randomised controlled trials (RCTs) is needed to cross-validate the available results in terms of validity, reliability, and generalisability of the programmes.

## Study objectives

The manualised Stress-Traumasymptoms-Arousal-Regulation-Treatment (START) programme was developed from clinical work with traumatised unaccompanied refugee minors in Germany to promote coping with traumatic distress and emotional irritability.^18–20^ An uncontrolled pilot study in 22 traumatised refugee minors showed positive effects on emotion regulation, adaptive strategies, self-control, distress and good feasibility.^18^ A subsequent study confirmed the positive effects on adaptive emotion regulation strategies.^21^

The primary aim of this RCT is to evaluate the efficacy of the START programme in reducing psychological distress and trauma symptoms in traumatised adolescent refugees compared with a waiting-list control group (WL). Main secondary aims are to assess whether treatment effects will remain stable for at least 12 weeks after programme termination (follow-up at week 24) and to assess whether the participants of the START groups improve emotion regulation strategies and mental health compared with WL. As stress and emotions are context-dependent and highly dynamic,^22^ intervention effects are assessed by psychometric instruments and with electronic ambulatory assessment (e-diaries). Ambulatory assessment aims to reduce retrospective biases while gathering ecologically valid data from everyday life near real-time. It has been shown as superior to retrospective psychometric assessment in terms of predicting symptom and treatment outcome^23–26^ and assessing reliably everyday functioning.^27^ We also evaluate intervention effects on the neuroendocrine stress system by assessing hair cortisol, which reflects the cumulative cortisol release over the past months and has been shown to capture treatment effects in PTSD.^28^

The study is part of the START research consortium, which evaluates distress-reducing psychotherapeutic group and preventive family interventions in traumatised toddler, adolescent and young adult refugees and aims to contribute to improved evidence-based therapeutic interventions for refugees within a stepped mental healthcare approach. The consortium comprises the START Adolescent Study, the START Childcare Study,^29^ the START Young Adults Study, and the smartphone-based experience sampling study tracking symptoms and daily living functioning with the use of e-diaries across the START Adolescents and Childcare studies. For further information regarding the consortium, please see wwwmentalhealth4refugeesde. The presented paper reports on the START Adolescents protocol (V.4.0, 16 March 2020) and has been conceived according to the Standard Protocol Items: Recommendations for Interventional Trials (SPIRIT) guidelines.^30 31^ Please also refer to SPIRIT checklist (online supplemental file 1) and to table 1.

10.1136/bmjopen-2021-057968.supp1 Supplementary data

**Table 1:** SPIRIT flow diagram

	Study period				
**Time point**	Screening	T0 Study visit Baseline 1 week after random. (WL) or before START Programme	T1 Study visit 8 weeks after T0 (WL) or after START Programme	T2 Study visit 4 weeks after T1 (WL) or at booster session (START)	T3 Study visit Follow-up 12 weeks after T2
**Enrolment**					
Eligibility screen	**X**				
Informed consent	**X**				
Randomisation	**X**				
**Assessments**					
Demographic data	**X**				
Medical history	**X**				
Medication/ psychotherapy	**X**	**X**	**X**	**X**	**X**
Saliva drug screening	**X**				
TONI-4	**X**				
ETI-CA	**X**		**X**	**X**	**X**
K-SADS-PL	**X**				
CGI-S	**X**		**X**	**X**	**X**
C-SSRS	**X**		**X**	**X**	**X**
REB		Weekly			
AUDIT	**X**				
Endangerm. to others	**X**		**X**	**X**	**X**
PSS-10	**X**	**X**	**X**	**X**	**X**
IES-R	**X**	**X**	**X**	**X**	**X**
SDQ self		**X**	**X**	**X**	**X**
SDQ caregiver	**X**		**X**	**X**	**X**
BDI-II		**X**	**X**	**X**	**X**
DERS18		**X**	**X**	**X**	**X**
cPTCI		**X**	**X**	**X**	**X**
CATS-2		**X**	**X**	**X**	**X**
DDNSI		**X**	**X**	**X**	**X**
PMLD		**X**			
Therapy expectation		**X**			
E-Diaries		**X**	**X**	**X**	**X**
Hair cortisol		**X**		**X**	**X**
AE assessments		**X**	**X**	**X**	**X**
**Intervention**					
START programme					
Booster session		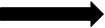		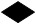	

AEs, adverse events; AUDIT, Alcohol Use Disorders Identification Test; BDI-II, Beck Depression Inventory, second edition; CATS-2, Child and Adolescent Trauma Screen-2; CGI-S, Clinical Global Impression Scale, severity of illness subscale; cPTCI, Post-traumatic Cognitions Inventory-child version; C-SSRS, Columbia-Suicide Severity Rating Scale; DDNSI, Disturbing Dream and Nightmare Severity Index; DERS-18, Difficulties in Emotion Regulation Scale, 18-Item; ETI-CA, Essen Trauma Inventory for Children and Adolescents; IES-R, Impact of Event Scale-Revised; K-SADS-PL, Kiddie-Schedule for Affective Disorders and Schizophrenia for School-Age Children-Present and Lifetime; PMLD, Post-Migration Living Difficulties Questionnaire; PSS-10, Perceived Stress Scale, 10-Item; SDQ, Strengths and Difficulties Questionnaire; SPIRIT, Standard Protocol Items: Recommendations for Interventional Trials; START, Stress-Traumasymptoms-Arousal-Regulation-Treatment; TONI 4, Test of Nonverbal Intelligence, Fourth Edition; WL, waiting-list.

### Methods and analysis

### Patient and public involvement

The original START programme was developed from clinical work with highly traumatised unaccompanied refugee minors of different countries of origin, who had recently arrived in Germany.^18–20^ The adapted and extended version of the programme, that is evaluated in this RCT, was redesigned according to the participants’ feedback of the pilot study groups, who were also traumatised refugee minors from different countries of origin. The public was not involved when designing the study.

### Trial design and setting

The study is conducted as a 12 weeks, two-arm, randomised, WL-controlled, multicentre trial with a 12-week follow-up and six participating outpatient departments in Germany, all of which are experienced in treating traumatised adolescent refugees. The study is coordinated by the Department of Child and Adolescent Psychiatry and Psychotherapy, University Medical Center, Johannes Gutenberg University, Mainz, Germany. The study intervention comprises an adapted version of the START programme.^18–20^ Languages used in the study for the intervention and the assessments are Arabic, Dari, English, German or Somali. Interpreters are involved if needed. The study is not blinded for the treatment condition, which will be evident to participants, caregivers and study therapists. Recruitment started 1 October 2020. We expect that the last participants will complete the study end of October 2022. Data base lock and analysis of primary outcomes are planned until end of December 2022.

Key stopping rules for patients are a withdrawal of informed consent, unwillingness to further participate in the trial, any factors affecting the patient’s or others’ well-being, for example, acute suicidality or acute endangerment to others, onset of other acute severe mental disorders, alcohol or substance abuse, inpatient treatment of over 2 days, start of concurrent psychotherapy and more than two psychotherapeutic crisis interventions. Key stopping rules for participating centres are non-adherence with the International Council for Harmonisation of Technical Requirements for Pharmaceuticals for Human Use: Guidelines for Good Clinical Practice (ICH-GCP)^32^ or the study protocol, insufficient recruitment of participants, or insufficient data quality. The key stopping rule of the trial is a change in the overall risk-benefit ratio.

## Participants

**Figure 1:**
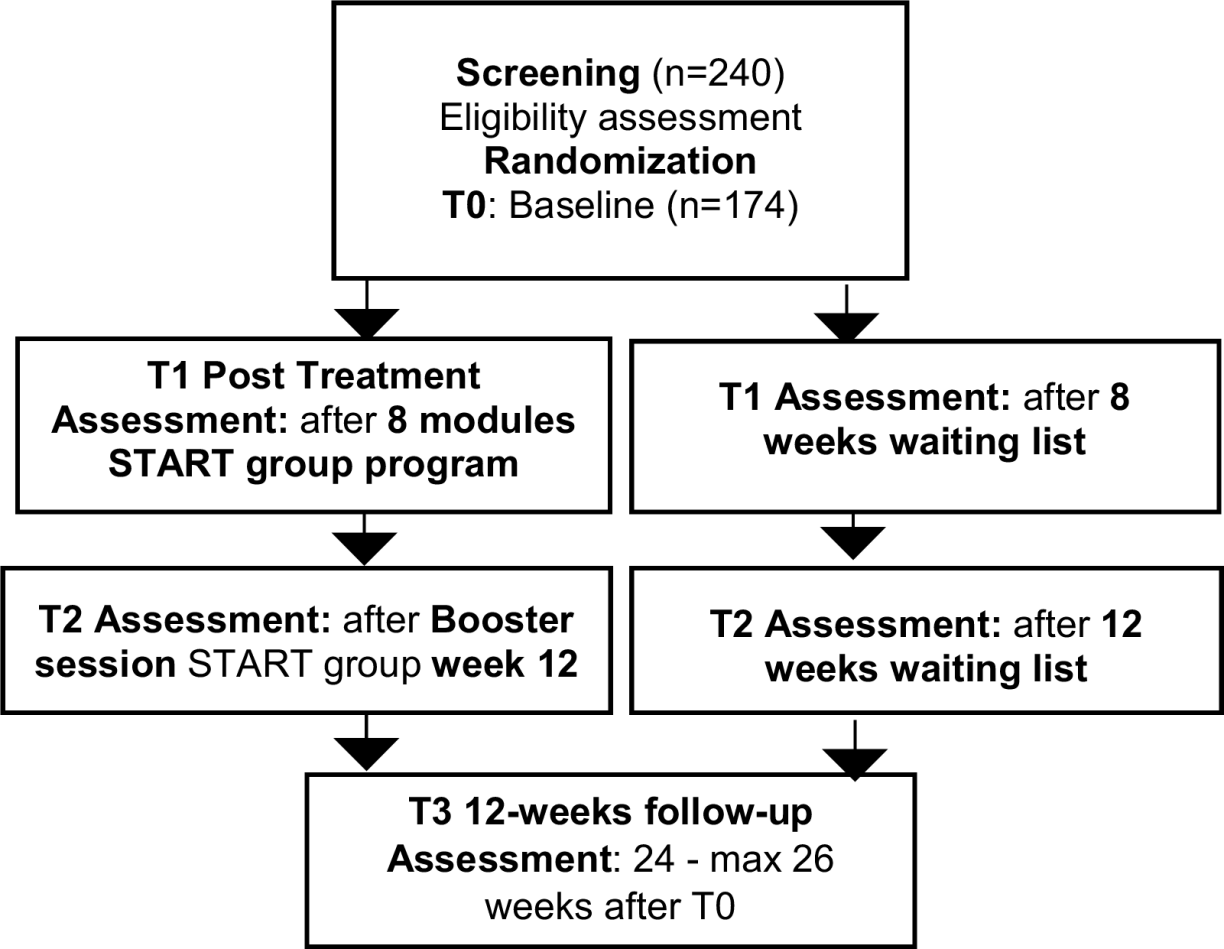
Participants‘ study flow. START, Stress-Traumasymptoms-Arousal-Regulation-Treatment.

We will allocate 174 participants to the trial and expect that we will have to screen 240 adolescent refugees for eligibility. Inclusion criteria are: informed consent by adolescents and caregivers, flight background, age between 13.00 and 17.11 years, verbal communication skills and reading comprehension in one of the study languages, non-verbal IQ ≥70, partial or full PTSD, PTSD symptoms are the clinically most impairing condition with at least moderate clinical symptom severity. We kept the exclusion criteria to a minimum, to foster the applicability of the programme in routine care as much as possible. Exclusion criteria are: current substance use disorder and/or harmful alcohol abuse, primary severe mental disorders other than PTSD, acute suicidality or danger to others, unstable psychotropic medication (change of psychotropic medication within the last 2 weeks before baseline assessment), current ongoing psychotherapy, inpatient status or study participation in another clinical trial, unaccompanied refugee minor without a legal representative, known pregnancy. Participants, who attend all assessment visits, will be given a study compensation. Participants will be insured by a clinical trial insurance during the time of study participation. Please see for detailed information about the assessment of eligibility criteria also the sections ‘screening’ and ‘psychometric instruments’. The participants’ study flow is provided in figure 1.

## Study conduct

### Recruitment

For recruitment, study information is provided to schools, child and adolescent psychotherapists/psychiatrists, social workers, youth welfare providers, at conferences, professional working group meetings, in newspaper articles or interviews, on the institutions’ homepages and on social media. All participants’ information about the study is provided in a caregiver and adolescents’ version in all study languages.

### Trial flow

For a summary of trial flow and assessment visits please see the SPIRIT flow diagram, table 1. All assessments are provided by trained study staff and are performed at the screening visit, baseline (T0), post-treatment (after 8-week START programme or 8-week waiting time; T1), after the booster session or 12-week waiting time (T2), and after 24 weeks at the 12-week follow-up (T3). The maximum time allowed between T0 and T3 is 26 weeks. After termination of the study, all participants are informed about their current diagnostic status and available interventions provided by child and adolescent psychiatric and psychotherapeutic routine care. Participants of the WL are given the opportunity to take part in independent START groups.

#### Screening visit

Assessment of informed consent for study participation, inclusion and exclusion criteria, demographic and medical data. Inclusion of eligible participants, randomisation to the intervention (START) or WL using a web-based randomisation system with permuted blocks stratified by centre (http://randomizer.at).

#### Baseline (T0), post-treatment (T1), booster session (T2), 12-week follow-up (T3) assessments

T0 takes place between one and 2 weeks after randomisation (WL) or before the first programme session (START), T1 within 1 week after the 8-week programme (START) or within 8–9 weeks after T0 (WL), T2 within 1 week after the booster session (START) or 4–5 weeks after T1 (WL) and T3 follow-up visits within 12 weeks after T2 (START/WL). At T0 only, the Post-Migration Living Difficulties Questionnaire (PMLD) and Questionnaire on Therapy Expectation are assessed. At the T0(T1)–T3 visits primary endpoints, trauma symptoms, emotion regulation, general psychopathology, overall clinical severity of mental disorders, suicidality and endangerment to others are assessed and 1 week e-diaries are applied. Hair cortisol is assessed at T0, T2 and T3. Adverse events are assessed at T0–T3 visits and weekly until T1.

### Intervention

Details of the START Adolescents programme are provided in table 2. START Adolescents study groups comprise three to eight participants and are restricted to study participants, who have been randomised to the intervention group. The manualised START group programme is based on DBT-techniques with emphasis on coping with emotional distress and traumatic symptoms, resilience, adaptive functioning and self-efficacy, but not on confrontation with traumatic memories. The group therapy is provided by two therapists through twice-weekly 60 min sessions and interactions are based on validation and acceptance techniques. Written participant programme materials are available in all study languages and if necessary, translators will attend the group therapies.

**Table 2:** Start adolescents programme, modules 1–8 and booster session

Before beginning of START: Written patient information. Handout. Every session: Mindfulness practice and stress signal light. Discussing between-session skills practice. Written patient material. Between the sessions: Participants are asked to train skills and techniques.		
	**Key interventions**	**Practice/training techniques**
**Module 1** Week 1 Sessions 1 and 2	Welcome. Introduction to START. Introduction of participants. Psychoeducation. Explanation of the concepts stress, high stress and stress-regulation skills. Teaching techniques. Skills for reducing distress and high distress. Relaxation techniques.	High-stress skills. Progressive muscle relaxation.
**Module 2** Week 2 Sessions 3 and 4	Consolidation: Available skills. Teaching techniques. Stress signal light for stress/inner tension monitoring and awareness. Emotion regulation strategies: mindful senses (hearing, feeling, tasting, watching, smelling). Skills box.	Mindful sensual experiences. Individual stress signal lights. Individual stimuli for distress/euthymia. High-stress skills. Individual skills boxes.
**Module 3** Week 3 Sessions 5 and 6	Consolidation. Individual skills boxes. Teaching techniques. Skills chains. Individual red light. Self-empowerment/resilience.	Skills chains Self-empowerment
**Module 4** Week 4 Sessions 7 and 8	Consolidation. Available skills. Psychoeducation. Explanation of the concept of crisis and management of crisis. Teaching techniques. Managing crisis: acceptance/skills/safe place. Managing flashbacks. Safety and emergency card. How to recognise change. How to change the moment?	Recognition and managing of crisis. Individual safe place. Skills that change the moment.
**Module 5** Week 5 Sessions 9 and 10	Consolidation. Available skills. Psychoeducation. Sleep disturbances and nightmares. Teaching techniques. Managing nightmares.	Telling/writing nightmares. Skills for managing nightmares.
**Module 6** Week 6 Sessions 11 and 12	Consolidation. Available skills. Psychoeducation. Emotions and emotion awareness. Teaching techniques. Emotion surfing ‘Letting go of emotional suffering’.	Recognising and naming emotions (emotion cards). Individual pleasant and unpleasant emotions. Emotion surfing.
**Module 7** Week 7 Sessions 13 and 14	Consolidation. Available skills, emotion recognition and understanding. Psychoeducation. Emotional network: Interdependence of emotions, thinking, behaviour and prompting stimuli. Skills for emotion regulation.	Role-play/competition: Recognising emotions and thoughts. Understand, name and regulate emotions and related behaviour and thoughts.
**Module 8** Week 8 Sessions 15 and 16	Review and consolidation: Available skills, current difficulties. Crisis plan. Close-out. Farewell celebration. Awarding the participants. Certificates: ‘You did your very best.’ Outlook to booster session.	Experiencing acquired/activated resources (balloon game). Discussing individual skills lists, open needs. Discussing crisis plan/designing emergency cards.
**Booster Session** Week 12 Session 17	Review and consolidation: Helpful skills, difficulties with skills practice within the last 4 weeks, current open needs. Small symbolic gift, for example, little gemstone.	Outlook for future/participants’ wishes/practicing how ‘to turn the tide’.

START, Stress-Traumasymptoms-Arousal-Regulation-Treatment.

The original START Adolescents programme comprised five modules with psychoeducation on trauma, stress, distressing emotions and crises, mindfulness training, arousal regulation, stress reduction techniques, and handling nightmares. For the current study, the START manual^18–20^ was extended by the three additional modules 6–8 and a booster session. The additional modules provide an intensified training of stress reduction skills, mindfulness techniques and additional psychoeducation about emotions and interpersonal effectiveness. Each session follows a defined structure and comprises, as fixed repeated elements, mindfulness training, monitoring one’s own inner tension/stress (‘stress signal light’), reviewing experiences when applying the programme skills, new contents/psychoeducation and skills training. The final session in module 8 is dedicated to a farewell ceremony and to validating and rewarding the participants' efforts and achievements. During the booster session, which is scheduled 4 weeks after programme termination, participants review their experiences when applying the acquired skills and techniques, their open needs, and provide an outlook on their personal future wishes and goals. If standardised risk assessments or participants’ personal information reveal acute danger to the self or others, additional psychotherapeutic crisis interventions will be offered to the participant. Participants with ongoing acute suicidal tendencies or ongoing danger to others after two subsequent crisis interventions must be excluded from study participation and transferred to specific intense psychiatric care.

## Assessments

### Psychometric instruments

For details of the instruments including psychometric properties, please see online supplemental file 2. All self-report assessments are applied in the study languages, all interviews are applied with the help of interpreters if indicated.

10.1136/bmjopen-2021-057968.supp2 Supplementary data

#### Inclusion and exclusion criteria

Inclusion and exclusion criteria are assessed with the Essen Trauma Inventory for Children and Adolescents,^33^ Clinical Global Impression Scale, severity of illness subscale (CGI-S^34^), the Kiddie-Schedule for Affective Disorders and Schizophrenia for School-Age Children-Present and Lifetime version,^35–37^ the Alcohol Use Disorders Identification Test,^38^ the Columbia-Suicide Severity Rating Scale,^39^ the Test of Nonverbal Intelligence, Fourth Edition,^40^ and the standardised Questionnaire to Assess Endangerment to Others.^39^

#### Primary endpoints

Psychological distress is assessed with the Perceived Stress Scale, 10-item version (PSS-10), a validated self-report questionnaire, that measures the intensity with which individuals appraise their daily life as stressful, unpredictable, uncontrollable and overloaded. Reliability and validity for the Arabic, English and German version have been shown to be sufficient or good.^41–44^ Traumatic symptoms are assessed with the Impact of Event Scale-Revised (IES-R), a validated, self-report questionnaire evaluating traumatic symptoms on the subscales intrusion, avoidance, hyperarousal and on an overall measure of traumatic distress. Reliability and validity for the Arabic, English and German version have been shown to be good.^45–48^

#### Secondary endpoints

We assess trauma-related psychosocial impairment and PTSD criteria according to Diagnostic and Statistical Manual of Mental Disorders, 5th ed. (DSM-5) with the Child and Adolescent Trauma Screen-2,^49^ trauma-related cognitions with the Posttraumatic Cognitions Inventory-child version^50–52^ and frequency and intensity of nightmares with the Disturbing Dream and Nightmare Severity Index.^53 54^ Emotion regulation is assessed with the 18-item version of the Difficulties in Emotion Regulation Scale (DERS-18^55^), general psychopathology with the Strengths and Difficulties Questionnaire,^56 57^ and the Beck Depression Inventory, second edition.^58 59^ Overall severity of illness is assessed with the CGI-S.^34^

#### Additional psychometric assessments

We assess adverse life experiences related to migration with the adapted German version of the self-rated PMLD,^60 61^ and therapy expectations with a self-designed questionnaire comprising three questions that are answered on a 5-point Likert-type scale.

### Ambulatory momentary assessment: electronic diaries

We use smartphone-based electronic (e)-diaries to assess aversive inner tension, emotional intensity, trauma symptoms (intrusions, hypervigilance), cognitive and behavioural avoidance of triggering situations in daily environments, sleep quality, stressfulness, content and number of nightmares, self-efficacy, self-esteem, rumination and somatisation.^62–64^ Filling in the answers takes a few minutes and items are available in all study languages. Rumination about cultural differences, social support and conflicts, discrimination experiences, and personally important events at school will be used as context variables to determine their associations with emotional distress and trauma symptoms. Participants receive a comprehensive explanation of the use of e-diaries as a spoken PowerPoint presentation and a written document in each participant’s respective language. After the presentation, the participant is asked to do a test trial on the smartphone. Smartphones are programmed with the e-diary app movisensXS^65^ and employ an hourly 1-week time-based e-diary design. Prompts are pseudorandomised within a time frame of 50–70 min to avoid expectancy effects. At the beginning of the study, participants are asked to enter their school schedules and determine their first prompt in the morning. On the weekends, e-diary assessments start at 10:00 hours no data are assessed during school hours. The prompts end at about 21:00 hours on school days and about 22:00 hours if there is no school the next day.

### Hair cortisol

If additional informed consent for hair cortisol collection is provided by study participants and their legal representatives, we collect thin hair strands from the posterior vertex of the head cut as close to the scalp as possible. Hair strands are tied together, sealed, and stored in a dry and dark place. Prior to analyses, they will be cut into a 2 cm segment proximal to the scalp, representing cumulative cortisol secretion of the last 2 months.^66^

### Quality assurance and monitoring

The study procedures are monitored by the Coordination Centre for Clinical Trials (KKS) Heidelberg according to the ICH-GCP^32^ with respect to a risk-based quality management strategy and ensure that the trial is conducted according to protocol and regulatory requirements. All data of the ongoing study are reviewed by an independent data monitoring committee once a year with special focus on safety issues.

Manual adherence across different therapists and participating centres is ensured by training all therapists on the START Adolescents programme and regular supervision with standardised assessments of manual adherence. We require a certain level of professional training from the therapists, of whom at least one per group must be a graduated psychologist, pedagogue, or physician.

The quality of psychometric assessments, tests and interviews is ensured by training the raters on the study assessments. Raters must have a bachelor’s or master’s degree in psychology or a comparable standard. Raters with a bachelor’s degree in psychology will administer questionnaires and psychological tests, raters with a Master‘s degree in psychology will administer the study interviews.

## Data management

The KKS Heidelberg is responsible for data management and analysis as well as data security and data transfer processes. All procedures are implemented in accordance with ICH-GCP guidelines and the Declaration of Helsinki.^32 67^ The protection of private data is ensured by a pseudonymisation procedure, and all private data are handled with respect to the European General Data Protection Regulation.^68^ Data are assessed by an electronic case report form (eCRF) and paper-based self-report questionnaires. The latter are transferred in copy to the KKS Heidelberg for double entry into the eCRF. E-diary data are transferred to the Mental mHealth lab, Karlsruhe Institute of Technology, analysed there and saved in encrypted form. The smartphones for e-diaries are provided by the research facility; no private smartphones are used for study purposes.

### Data availability

The research data generated during this study will be available on reasonable request by the study coordinating centre at the Department of Child and Adolescent Psychiatry, University Medical Center, Mainz, Germany. Anonymised data use by other researchers not involved in the study may be done with prior agreement.

## Statistics

### Sample size calculation

The sample size calculation is based on the coprimary outcomes of emotional distress (PSS-10) and traumatic symptoms (IES-R) at T1. Based on available studies^69 70^ we assume in our study an effect size of *d*=0.63 for the PSS-10 and of *d*=0.56 for the IES-R score at T1. This results in a required sample size of n=84 (PSS-10) and n=104 (IES-R) for a one-sided test at an *α*=0.025 level with a power of 80% to detect such differences. The START pilot trial showed a drop-out rate <10% and was conducted as a one-centre single-arm naturalistic trial with mostly inpatients.^20^ We, therefore, calculated the sample size on the assumption of 40% drop-outs between T0 and T1 to account for the multicentre, WL-controlled approach and unstable living conditions of the included population. This amounts to n=174 participants who need to be randomised in our trial.

### Analysis population and analysis

All randomised participants will be included in the full-analysis set as allocated. All randomised participants, who finish the study according to the study protocol until T2 as planned with no missing values for T1 (primary endpoint) will be included in the per-protocol set for analysis as allocated. All randomised participants will be included in the safety population according to the applied intervention.

Baseline characteristics will be analysed descriptively for the safety, full-analysis, and per-protocol set in the intervention and control group. The primary and coprimary endpoints will be analysed in the full-analysis set. Missing values will be imputed. Endpoints will be assessed as response variables in linear mixed regression models with site and psychotherapeutic group as random effects, intervention group, sex and region of origin as fixed effects, and age, PSS-10 and IES-R baseline scores, respectively, as linear effects with a hierarchical testing procedure that maintains the overall significance level of *α*=0.025. The primary analysis will be repeated in the per-protocol population as a sensitivity analysis. Since the different timing of the T0 baseline examination in the study groups after randomisation could cause biases in the effect estimates, that cannot be estimated or modelled, additional sensitivity analyses will be performed, such as repeating the primary analysis using the screening value instead of the baseline value. Secondary outcomes will be analysed in the full-analysis set. Missing values will not be imputed. The stability of treatment effects up to T3 will be assessed by calculating confidence intervals on the T1–T3 and T2–T3 difference for the PSS-10 and IES-R total scores in the intervention group. Psychometric scales used for assessment of secondary outcomes and hair cortisol concentrations will be analysed according to primary endpoints or descriptively. For the analysis of e-diary data, multilevel models will be used to characterise momentary mechanisms and context-dependent affective experiences, which allow a nested data structure (momentary experiences within participants), and enable different numbers of ratings per participant to be handled quite well.^71^ Safety variables will be analysed in the safety and full-analysis population.

### Ethics and dissemination

The study protocol, patient recruitment procedures, patients’ information and informed consent material have been approved by the lead ethics committee of Rhineland-Palatinate (ID 2019-14709) and the ethics committees of participating sites. For every substantial protocol modification approval of all ethics committees is required. The study will be conducted according to ICH-GCP^32^ and the Declaration of Helsinki.^67^ Study results will be published through peer-reviewed publications and presented at scientific and clinical conferences.

## Trial status

Recruitment started 1 October 2020.

## Discussion

Evidence-based, evaluated, low-threshold and culture-sensitive psychotherapeutic treatment programmes, that reduce distress caused by traumatic experiences that can be efficiently established in routine mental healthcare, would represent valuable therapeutic interventions to support refugee minors with trauma-related symptoms and emotion regulation difficulties. However, such interventions are currently lacking. The primary aim of this study is to evaluate the clinical efficacy and intermediate outcome of the distress-reducing psychotherapeutic START programme and to contribute to improved evidence-based therapeutic interventions for adolescent refugees within a stepped-care mental health approach in Germany. If shown to be effective, the first rigorously evaluated manualised intervention programme for traumatised adolescent refugees will be available, which will have widespread implications for clinical practice.

Some limitations for the application of the programme in mental health routine care in host countries may be that the therapeutic study groups are limited only to traumatised refugees, which may not reflect clinical reality. Another limitation is the restriction of study participation to refugee minors with language skills in Arabic, Dari, English, German and Somali. Thus, the study will not provide information on the effectiveness of the programme if applied to refugee minors with a different cultural or language background. However, since all programme materials are available in Arabic, Dari, English, German and Somali, the START programme will provide a treatment option not only in German-speaking or English-speaking host countries that care for refugee minors but also in refugee camps based in Arabic-speaking, Dari-speaking or Somali-speaking countries, where is a huge need for easy-to-apply psychotherapeutic treatment options.

Beyond effects on the symptom level, we will analyse intervention effects on psychosocial functioning and everyday behaviour through the use of e-diaries and on the neuroendocrine stress system by analysing hair cortisol. The multimodal assessment approach of symptoms, functioning, daily living outcomes, context variables and neuroendocrine effects, as well as the follow-up assessment, will enable us to analyse immediate and intermediate treatment effects not only on the phenomenological clinical level but also on the biological and daily living level. This will extend our understanding of intervention effects, influencing factors and the course of trauma-related mental health problems in refugee minors.

## Supplementary Material

Reviewer comments

Author's manuscript

## References

[1] United Nations High Commissioner for Refugees . Global trends forced displacement in 2019. Denmark: UNHCR, 2020.

[2] Office for Migration and Refugees . Schlüsselzahlen Asyl 2019. Nürnberg: Bundesamt für Migration und Flüchtlinge, 2020.

[3] Office for Migration and Refugees . Flüchtlinge (2019): Aktuelle Zahlen, 2019.

[4] Höhne E , van der Meer AS , Kamp-Becker I , et al . A systematic review of risk and protective factors of mental health in unaccompanied minor refugees. Eur Child Adolesc Psychiatry 2020. 10.1007/s00787-020-01678-2. [Epub ahead of print: 09 Nov 2020]. PMC9343263 33169230

[5] Müller LRF , Gossmann K , Hartmann F , et al . 1-year follow-up of the mental health and stress factors in asylum-seeking children and adolescents resettled in Germany. BMC Public Health 2019;19:1–11. 10.1186/s12889-019-7263-6 31286909 PMC6615278

[6] Müller LRF , Büter KP , Rosner R , et al . Mental health and associated stress factors in accompanied and unaccompanied refugee minors resettled in Germany: a cross-sectional study. Child Adolesc Psychiatry Ment Health 2019;13:1–13. 10.1186/s13034-019-0268-1 30719070 PMC6352340

[7] Walg M , Fink E , Großmeier M , et al . Häufigkeit psychischer Störungen bei unbegleiteten minderjährigen Flüchtlingen in Deutschland. Zeitschrift für Kinder- und Jugendpsychiatrie und Psychotherapie 2017;45:58–68. 10.1024/1422-4917/a000459 27550438

[8] Anders M , Christiansen H . Die Versorgung unbegleiteter minderjähriger Flüchtlinge – eine systematische Literaturübersicht zu psychologischen Interventionen. Kindheit und Entwicklung 2016;25:216–30.

[9] Sierau S , Schneider E , Nesterko Y . Psychische Belastung bei unbegleiteten jungen Geflüchteten in Jugendhilfeeinrichtungen. Psychiatr Prax 2019;46:135–40. 10.1055/a-0756-7970 30380585

[10] Kien C , Sommer I , Faustmann A , et al . Prevalence of mental disorders in young refugees and asylum seekers in European countries: a systematic review. Eur Child Adolesc Psychiatry 2019;28:1295–310. 10.1007/s00787-018-1215-z 30151800 PMC6785579

[11] Tam SY , Houlihan S , Melendez-Torres GJ . A systematic review of longitudinal risk and protective factors and correlates for posttraumatic stress and its natural history in forcibly displaced children. Trauma Violence Abuse 2017;18:377–95. 10.1177/1524838015622437 26721887

[12] Barron IG , Abdallah G , Smith P . Randomized control trial of a CBT trauma recovery program in Palestinian schools. Journal of Loss and Trauma 2013;18:306–21. 10.1080/15325024.2012.688712

[13] Tyrer RA , Fazel M . School and community-based interventions for refugee and asylum seeking children: a systematic review. PLoS One 2014;9:e89359. 10.1371/journal.pone.0089359 24586715 PMC3933416

[14] Pfeiffer E , Goldbeck L . Evaluation of a trauma‐focused group intervention for unaccompanied young refugees: a pilot study. J Trauma Stress 2017;30:531–6. 10.1002/jts.22218 28992383

[15] Gormez V , Kılıç HN , Orengul AC , et al . Evaluation of a school-based, teacher-delivered psychological intervention group program for trauma-affected Syrian refugee children in Istanbul, Turkey. Psychiatry and Clinical Psychopharmacology 2017;27:125–31. 10.1080/24750573.2017.1304748

[16] Nocon A , Eberle-Sejari R , Unterhitzenberger J , et al . The effectiveness of psychosocial interventions in war-traumatized refugee and internally displaced minors: systematic review and meta-analysis. Eur J Psychotraumatol 2017;8:1388709. 10.1080/20008198.2017.1388709 29163868 PMC5687794

[17] Demazure G , Gaultier S , Pinsault N . Dealing with difference: a scoping review of psychotherapeutic interventions with unaccompanied refugee minors. Eur Child Adolesc Psychiatry 2018;27:447–66. 10.1007/s00787-017-1083-y 29214387

[18] Dixius A , Möhler E . Start – Entwicklung einer intervention zur Erststabilisierung und Arousal-Modulation für stark belastete minderjährige Flüchtlinge. Prax Kinderpsychol Kinderpsychiatr 2017;66:277–86. 10.13109/prkk.2017.66.4.277 28393646

[19] Dixius A , Möhler E . Ein neues Therapie-Konzept validiert die besonderen Bedürfnisse geflüchteter Kinder und Jugendlicher: start. Psychother Forum 2017;22:76–85. 10.1007/s00729-017-0095-x

[20] Dixius A , Stevens A , Moehler E . A pilot evaluation study of an intercultural treatment program for stabilization and arousal modulation for intensely stressed children and adolescents and minor refugees, called start (Stress-Traumasymptoms-Arousal-Regulation-Treatment). ARC Journal of Psychiatry 2017;2:7–24.

[21] Dixius A , Möhler E . Feasibility and effectiveness of a new short-term psychotherapy concept for adolescents with emotional dysregulation. Front Psychiatry 2020;11:1630. 10.3389/fpsyt.2020.585250 PMC7858646 33551862

[22] Ebner-Priemer UW , Eid M , Kleindienst N , et al . Analytic strategies for understanding affective (in)stability and other dynamic processes in psychopathology. J Abnorm Psychol 2009;118:195–202. 10.1037/a0014868 19222325

[23] Forbes EE , Stepp SD , Dahl RE , et al . Real-world affect and social context as predictors of treatment response in child and adolescent depression and anxiety: an ecological momentary assessment study. J Child Adolesc Psychopharmacol 2012;22:37–47. 10.1089/cap.2011.0085 22339611 PMC3281286

[24] Peeters F , Berkhof J , Rottenberg J , et al . Ambulatory emotional reactivity to negative daily life events predicts remission from major depressive disorder. Behav Res Ther 2010;48:754–60. 10.1016/j.brat.2010.04.008 20537317

[25] Wichers M , Lothmann C , Simons CJP , et al . The dynamic interplay between negative and positive emotions in daily life predicts response to treatment in depression: a momentary assessment study. Br J Clin Psychol 2012;51:206–22. 10.1111/j.2044-8260.2011.02021.x 22574805

[26] Wichers M , Peeters F , Geschwind N , et al . Unveiling patterns of affective responses in daily life may improve outcome prediction in depression: a momentary assessment study. J Affect Disord 2010;124:191–5. 10.1016/j.jad.2009.11.010 20004977

[27] Santangelo PS , Reinhard I , Koudela-Hamila S , et al . The temporal interplay of self-esteem instability and affective instability in borderline personality disorder patients' everyday lives. J Abnorm Psychol 2017;126:1057–65. 10.1037/abn0000288 29154566

[28] Steudte-Schmiedgen S , Kirschbaum C , Alexander N , et al . An integrative model linking traumatization, cortisol dysregulation and posttraumatic stress disorder: insight from recent hair cortisol findings. Neurosci Biobehav Rev 2016;69:124–35. 10.1016/j.neubiorev.2016.07.015 27443960

[29] Mayer A , Taubner S , Bark C , et al . Herausforderungen in der frühpädagogischen Arbeit mit geflüchteten Familien mentalisierungsbasiert begegnen. Prax Kinderpsychol Kinderpsychiatr 2019;68:711–27. 10.13109/prkk.2019.68.8.711 31957564

[30] Chan A-W , Tetzlaff JM , Altman DG , et al . Spirit 2013: new guidance for content of clinical trial protocols. Lancet 2013;381:91–2. 10.1016/S0140-6736(12)62160-6 23305999

[31] Chan A-W , Tetzlaff JM , Altman DG , et al . Spirit 2013 statement: defining standard protocol items for clinical trials. Ann Intern Med 2013;158:200–7. 10.7326/0003-4819-158-3-201302050-00583 23295957 PMC5114123

[32] ICH Expert Working Group . ICH Harmonised tripartite guideline for good clinical practice E6 (R1). ICH Harmon Tripart Guidel 1996;1996.

[33] Graham FS W , Tagay S . ETI-CA Essen Trauma-Inventory for Children and Adolescents - Interview Essen: LVR-Klinikum Essen, Universität Duisburg, 2012.

[34] Guy W . Clinical global impression. Assessment manual for Psychopharmacology 1976:217–22.

[35] Kaufman J , Birmaher B , Brent D , et al . Schedule for affective disorders and schizophrenia for school-age children-present and lifetime version (K-SADS-PL): initial reliability and validity data. J Am Acad Child Adolesc Psychiatry 1997;36:980–8. 10.1097/00004583-199707000-00021 9204677

[36] American Psychiatric Association . Diagnostic and statistical manual of mental disorders. 5th ed. Washington, DC, 2013.

[37] Kaufman J , Birmaher B , Axelson D . Schedule for affective and disorders and schizophrenia for school aged children (6–18 years): Kiddie-SADS-lifetime version (K-SADS-PL DSM 5). Pittsburgh, PA: Western Psychiatric Institute and Clinic, 2016.

[38] National Institute on Drug Abuse . Audit, 2013.

[39] Posner K , Brown GK , Stanley B , et al . The Columbia-Suicide severity rating scale: initial validity and internal consistency findings from three multisite studies with adolescents and adults. Am J Psychiatry 2011;168:1266–77. 10.1176/appi.ajp.2011.10111704 22193671 PMC3893686

[40] Brown L , Sherbenou RJ , Johnsen SK . Test of nonverbal intelligence: TONI-4: Pro-ed. Austin, TX, 2010.

[41] Cohen S , Williamson G . Perceived stress in a probability sample of the United States. In: Spacapan S , Oskamp S , eds. The social psychology of health: Claremont Symposium on applied social psychology. Newbury Park, CA: Sage, 1988: 31–67.

[42] Klein EM , Brähler E , Dreier M , et al . The German version of the perceived stress scale – psychometric characteristics in a representative German community sample. BMC Psychiatry 2016;16:1–10. 10.1186/s12888-016-0875-9 27216151 PMC4877813

[43] Musial F , Büssing A , Heusser P , et al . Mindfulness-based stress reduction for integrative cancer care – a summary of evidence. Complement Med Res 2011;18:192–202. 10.1159/000330714 21934319

[44] Chaaya M , Osman H , Naassan G , et al . Validation of the Arabic version of the Cohen perceived stress scale (PSS-10) among pregnant and postpartum women. BMC Psychiatry 2010;10:111. 10.1186/1471-244X-10-111 21159169 PMC3016315

[45] Weiss DS , Marmar CR . The impact of event scale-revised. assessing psychological trauma and PTSD. New York, NY, US: The Guilford Press, 1997: 399–411.

[46] Weiss DS . The impact of event scale-revised. assessing psychological trauma and PTSD. 2nd ed. New York, NY, US: The Guilford Press, 2004: 168–89.

[47] Maercker A , Schützwohl M . Erfassung von psychischen Belastungsfolgen: die impact of event Skala-revidierte version (IES-R). Diagnostica 1998.

[48] Davey C , Heard R , Lennings C . Development of the Arabic versions of the impact of events Scale‐Revised and the posttraumatic growth inventory to assess trauma and growth in middle Eastern refugees in Australia. Clin Psychol 2015;19:131–9. 10.1111/cp.12043

[49] Sachser C , Berliner L , Holt T , et al . International development and psychometric properties of the child and adolescent trauma screen (cats). J Affect Disord 2017;210:189–95. 10.1016/j.jad.2016.12.040 28049104

[50] Meiser-Stedman R , Smith P , Bryant R , et al . Development and validation of the child post-traumatic cognitions inventory (CPTCI). J Child Psychol Psychiatry 2009;50:432–40. 10.1111/j.1469-7610.2008.01995.x 19338628

[51] de Haan A , Petermann F , Meiser-Stedman R , et al . Psychometric properties of the German version of the child post-traumatic cognitions inventory (CPTCI-GER). Child Psychiatry Hum Dev 2016;47:151–8. 10.1007/s10578-015-0552-0 25990307

[52] Meiser-Stedman R . Post-traumatic Cognitions Inventory - Child Version (Arabic version), n. d.. Available: https://www.childrenandwar.org/wp-content/uploads/2019/06/CPTCI_arabisch.pdf

[53] Krakow B . Nightmare complaints in treatment-seeking patients in clinical sleep medicine settings: diagnostic and treatment implications. Sleep 2006;29:1313–9. 10.1093/sleep/29.10.1313 17068985

[54] Krakow B , Haynes PL , Warner TD , et al . Nightmares, insomnia, and sleep-disordered breathing in fire evacuees seeking treatment for posttraumatic sleep disturbance. J Trauma Stress 2004;17:257–68. 10.1023/B:JOTS.0000029269.29098.67 15253098

[55] Victor SE , Klonsky ED . Validation of a brief version of the difficulties in emotion regulation scale (DERS-18) in five samples. J Psychopathol Behav Assess 2016;38:582–9. 10.1007/s10862-016-9547-9 PMC4882111 27239096

[56] Goodman R . Psychometric properties of the strengths and difficulties questionnaire. J Am Acad Child Adolesc Psychiatry 2001;40:1337–45. 10.1097/00004583-200111000-00015 11699809

[57] et al Idzelis M , Ali AS , Gaaddasaar M . n. d.. Available: https://www.sdqinfo.org/py/sdqinfo/b3.py?language=Somali

[58] Beck AT , Steer RA , Brown GK . Bdi-ii manual, 1996.

[59] Selmo P , Koch T , Brand J , et al . Psychometric properties of the online Arabic versions of BDI-II, HSCL-25, and PDS. European Journal of Psychological Assessment 2019;35:46–54. 10.1027/1015-5759/a000367

[60] Zentrum Überleben . n. d.. Available: https://www.ueberleben.org/

[61] Refugio Munich . Refugio Munich, n. d.. Available: https://www.refugio-muenchen.de/

[62] Pfaltz MC , Michael T , Meyer AH , et al . Reexperiencing symptoms, dissociation, and avoidance behaviors in daily life of patients with PTSD and patients with panic disorder with agoraphobia. J Trauma Stress 2013;26:443–50. 10.1002/jts.21822 23893375

[63] Price M , Ruggiero KJ , Ferguson PL , et al . A feasibility pilot study on the use of text messages to track PTSD symptoms after a traumatic injury. Gen Hosp Psychiatry 2014;36:249–54. 10.1016/j.genhosppsych.2014.02.004 24636721 PMC4090249

[64] Ruscio AM , Gentes EL , Jones JD , et al . Rumination predicts heightened responding to stressful life events in major depressive disorder and generalized anxiety disorder. J Abnorm Psychol 2015;124:17–26. 10.1037/abn0000025 25688429 PMC4332541

[65] movisens GmbH, 2021. Available: https://www.movisens.com/de/

[66] Short SJ , Stalder T , Marceau K , et al . Correspondence between hair cortisol concentrations and 30-day integrated daily salivary and weekly urinary cortisol measures. Psychoneuroendocrinology 2016;71:12–18. 10.1016/j.psyneuen.2016.05.007 27235635 PMC4955743

[67] World Medical Association . World Medical association Declaration of Helsinki: ethical principles for medical research involving human subjects. JAMA 2013;310:2191–4. 10.1001/jama.2013.281053 24141714

[68] General Data Protection Regulation GDPR . n. d. Available: https://gdpr-info.eu/

[69] Prasad L , Varrey A , Sisti G . Medical students' stress levels and sense of well being after six weeks of yoga and meditation. Evid Based Complement Alternat Med 2016;2016:1–7. 10.1155/2016/9251849 PMC5174168 28053644

[70] Butollo W , Karl R , König J , et al . A randomized controlled clinical trial of dialogical exposure therapy versus cognitive processing therapy for adult outpatients suffering from PTSD after type I trauma in adulthood. Psychother Psychosom 2016;85:16–26. 10.1159/000440726 26610167

[71] Raudenbush SW , Bryk AS . Hierarchical linear models: applications and data analysis methods. Sage Publications, 2002.

