# Supplementary material for: START adolescents: study protocol of a randomised controlled trial to investigate the efficacy of a low-threshold group treatment programme in traumatised adolescent refugees
Source: BMJ Open. 2021 Dec 28;11(12):e057968. doi: 10.1136/bmjopen-2021-057968 (PMC8719222; doi:10.1136/bmjopen-2021-057968)
Supplement: Supplementary data [file bmjopen-2021-057968supp002.pdf]

Psychometric instruments

If not available psychometric properties for internal consistency and reliability, criterion validity and convergent validity will be calculated within our sample.

Inclusion and exclusion criteria

Essen Trauma Inventory for Children and Adolescents

The Essen Trauma Inventory for Children and Adolescents (ETI-CA; (1, 2)) comprises a list of traumatic events and assesses symptoms of acute adjustment disorder and PTSD according to DSM-IV criteria (3) within the last four weeks, in relation to the most disturbing traumatic event if more than one was experienced. It further assesses the degree of impairment in six areas of daily living (e.g., school, peers) due to the traumatic event. It is available as a self-report questionnaire and an interview version, with the latter being used in this study. ETI-CA has been designed for the age group 12-17 years and is available in German and English. If necessary, we will translate the ETI-CA interview into Arabic, Dari, and Somali.

Clinical Global Impression Scale – Severity of illness

The Clinical Global Impression Scale is an internationally used instrument and yields the three different measures: 1. Severity of illness (CGI-S), 2. Global improvement (CGI-I), and 3. Efficacy index (4). In our study, we will apply the CGI-S, which is a 7-point scale on which the clinician rates the severity of the patient's illness at the time of assessment relative to the clinician's past experience with patients with the same diagnosis. Possible ratings range from 1=not ill at all to 7=among the most extremely ill patients. The German version of the scale (4) is clinically rated by study staff.

Kiddie-Schedule for Affective Disorders and Schizophrenia, Present and Lifetime

Sobanski E, *et al. BMJ Open* 2021; 11:e057968. doi: 10.1136/bmjopen-2021-057968

The Schedule for Affective Disorders and Schizophrenia for School-Age Children-Present and Lifetime version (K-SADS-PL) is a widely used, validated, semi-structured diagnostic interview for the standardized assessment of mental disorders according to DSM-5 in children and adolescents aged 6-18 years (5-7). For the purpose of this study we will use the K-SASDS-PL diagnostic screening interview with subsequent diagnostic interviews if indicated, and the summary lifetime diagnosis checklist. The diagnostic screening interview reviews the most severe current and past major symptoms of all diagnoses assessed with the K-SADS-PL. If the participant displays indicative symptoms of a specific disorder according to the screening, the referring diagnostic interviews will be applied. The K-SADS-PL comprises the following diagnostic interviews: depressive and bipolar-related disorders, psychotic disorders, anxiety disorders, obsessive-compulsive disorders, trauma-related disorders including PTSD, externalizing disorders, eating disorders, and substance abuse disorders. The summary lifetime diagnosis checklist then summarizes all mental diagnoses assessed by the K-SADS-PL. The K-SADS-PL is available in German and English for the purpose of the study If needed, the interview is conducted with the assistance of an interpreter.

Alcohol Use Disorders Identification Test

The Alcohol Use Disorders Identification Test (AUDIT) is an internationally used, validated, self-report questionnaire approved by the World Health Organization to screen individuals for hazardous or harmful alcohol consumption and alcohol dependence. It can be applied from age 14 years onwards. Items are rated on a 5-point Likert scale (0-4). Total possible AUDIT scores range from 0-40. Scores of 8-10 or more are indicative of hazardous, harmful alcohol use or possible alcohol dependence. The AUDIT is freely available in many languages, including Arabic,

English and German (8). For the purpose of our study, we translated the questionnaire into Dari and Somali.

Columbia-Suicide Severity Rating Scale

The Columbia-Suicide Severity Rating Scale (C-SSRS) assesses suicidal ideation and behavior. Questions are phrased for use in an interview format. The C-SSRS measures the following constructs: severity of suicidal ideation, intensity of suicidal ideation, suicidal and/or self-harm behavior, and lethality of suicide attempts. It is provided in a lifetime and recent/since last visit version (9). We use the lifetime version for the first assessment at the screening visit and the recent/since last visit version for all other study visits. In addition to the original validated English version, the C-SSRS is available in a German version and has been translated into Arabic, Dari, and Somali.

Test of Nonverbal Intelligence, fourth edition

The Test of Nonverbal Intelligence, Fourth Edition (TONI 4) is a language-free, validated intelligence test that requires no reading, writing or speaking on the examinee's part. It can be used from the age of six years onwards. Test items present a variety of problem-solving tasks in ascending order of difficulty. The abstract, figural content of the test items reduces the cultural loading of the test and the result is not influenced by insufficient language skills (10).

Questionnaire to Assess Endangerment to Others

The standardized Questionnaire to Assess Endangerment to Others is a self-designed, self-rated questionnaire in accordance with the wording of the C-SSRS (9), which assesses thoughts or behaviors within the last six months displaying endangerment or threat to others. The questionnaire comprises six questions that are answered in a yes/no structure and is provided in Arabic, Dari, English, German and Somali.

Sobanski E, *et al. BMJ Open* 2021; 11:e057968. doi: 10.1136/bmjopen-2021-057968

Primary endpoints

Perceived Stress Scale, 10-item version

The Perceived Stress Scale, 10-item version (PSS-10), is a validated self-report questionnaire that assesses psychological distress during the preceding month. It measures the intensity with which individuals appraise their daily life as stressful, unpredictable, uncontrollable, and overloaded. The PSS-10 was designed for use in participants aged 14 years or older. Items are rated on a 6-point scale from 0=never to 5=often. In addition to the original English version (11), a validated German version (12) and an Arabic version are available (13). For the purpose of this study, we translated the PSS-10 into Dari and Somali.

Impact of Event Scale-Revised

The Impact of Event Scale-Revised (IES-R) is a validated, self-report questionnaire assessing traumatic symptoms on three subscales: intrusion (intrusive thoughts, nightmares, intrusive feelings and imagery, dissociative-like re-experiencing), avoidance (numbing of responsiveness, avoidance of feelings, situations and ideas), hyperarousal (anger, irritability, hypervigilance, difficulty concentrating, heightened startle), and an overall measure of traumatic distress. Items are rated on a 5-point Likert scale from 0=not at all to 4=extreme. In addition to the original English version (14, 15), a validated German version (16) and an Arabic version (17) are available. For the purpose of this study, we translated the IES-R into Dari and Somali.

Secondary endpoints

Trauma symptoms

Child and Adolescent Trauma Screen-2

The Child and Adolescent Trauma Screen-2 (CATS-2) is a validated, freely accessible self-report questionnaire based on the DSM-5 PTSD criteria. It assesses DSM-5

criteria A (experience of a traumatic event ever), B (intrusion, re-experiencing), C (avoidance), D (negative alterations in cognitions and mood), and E (hyperarousal). Criteria B-D are rated on a 4-point scale (0=never, 3=almost always). Trauma-related psychosocial impairment (peers, family, school/work, hobbies, well-being) is assessed with yes/no items. For the purpose of this study, we use the validated English and German CATS-2 self-report versions (18) and translated the scale into Arabic, Dari and Somali.

Posttraumatic Cognitions Inventory - child version

The Posttraumatic Cognitions Inventory - child version (cPTCI) is a validated, self-report questionnaire assessing trauma-related cognitions on the subscales 'permanent and disturbing change', 'fragile person in a scary world' and on an overall score. Items are rated on a 5-point Likert-type scale and are summed to form a total score and the two subscales scores. For the purpose of this study, we use the validated English version (19), a validated German (20) and an Arabic cPTCI version (21) and translated the cPTCI into Dari and Somali.

Disturbing Dream and Nightmare Severity Index

The Disturbing Dream and Nightmare Severity Index (DDNSI) is a validated, self-report questionnaire and assesses number of nights with nightmares per week (0-7 nights), total number of nightmares per week, severity of nightmares, intensity of nightmares (Likert-type scale: 0=no problem to 6=extremely severe problem), how often nightmares result in awakenings (0=never/rarely to 4=always). The scale provides five sub-scores and a total score (22, 23). For the purpose of this study, the DDNSI was translated into Arabic, Dari, German, and Somali.

Emotion Regulation

Difficulties in Emotion Regulation Scale

Sobanski E, *et al. BMJ Open* 2021; 11:e057968. doi: 10.1136/bmjopen-2021-057968

The 18-item version of the Difficulties in Emotion Regulation Scale (DERS-18; (24)) is a validated, self-report questionnaire assessing the following six components of emotion dysregulation on a 5-point Likert-type scale: 1) Nonacceptance of emotional responses, 2) Difficulties engaging in goal-directed behavior, 3) Impulse control difficulties, 4) Lack of emotional awareness, 5) Limited access to emotion regulation strategies, 6) Lack of emotional clarity. It can be used from the age of 13 years onwards and yields a total score as well as subscale scores. For the purpose of the study, we use the 18 items from the unpublished German validation study of the DERS-36 (25, 26) and translated the English version of the DERS-18 (24) into Arabic, Dari and Somali.

General Psychopathology

Strengths and Difficulties Questionnaire

The Strengths and Difficulties Questionnaire (SDQ) is a widely used, validated parent-/caregiver-rated and/or self-report screening questionnaire for children and adolescents that is freely available online in an English version (27), German version (28), Arabic version (29), and Somali parent/caregiver version (30). The SDQ screens for difficulties in four areas: 1) emotional symptoms, 2) conduct problems, 3) hyperactivity/inattention, and 4) peer relationship problems. It additionally assesses strengths in prosocial behavior and provides a total score that sums up all subscale scores. For the purpose of this study, we use the available Arabic, English, German, self-report, and caregiver SDQ as well as the Somali caregiver version, and translated the SDQ caregiver and self-report version into Dari and the self-report version into Somali.

Beck Depression Inventory, second edition

The Beck Depression Inventory, second edition (BDI-II) is a widely used, validated, self-report questionnaire that assesses the severity of depression according to DSM-

Sobanski E, *et al. BMJ Open* 2021; 11:e057968. doi: 10.1136/bmjopen-2021-057968

IV criteria on a 4-point Likert-like scale. It can be used in adolescents from age 13 years onwards. The BDI-II is available in a validated English (31), validated German (32) and a validated Arabic version (33), which we use in this study as well as a translated Dari and Somali version

References

1. Graham FS, W, Tagay S. ETI-CA Essen Trauma-Inventory for Children and Adolescents - Interview Essen: LVR-Klinikum Essen, Universität Duisburg; 2012.
2. Tagay S, Düllmann S, Hermans E, Repic N, Hiller R, Senf W. Das Essener Trauma-Inventar für Kinder und Jugendliche (ETI-KJ). Zeitschrift für Kinder-und Jugendpsychiatrie und Psychotherapie. 2011;39:323-40.
3. American Psychiatric Association. Diagnostic and Statistical Manual of Mental Disorders (DSM-IV) Fourth Edition. Washington, DC: American Psychiatric Association; 1994.
4. Guy W. Clinical global impression. Assessment manual for Psychopharmacology. 1976:217-22.
5. Kaufman J, Birmaher B, Brent D, Rao U, Flynn C, Moreci P, et al. Schedule for affective disorders and schizophrenia for school-age children-present and lifetime version (K-SADS-PL): initial reliability and validity data. Journal of the American Academy of Child & Adolescent Psychiatry. 1997;36(7):980-8.
6. American Psychiatric Association. Diagnostic and Statistical Manual of Mental Disorders. 5th ed. Washington, DC2013.
7. Kaufman J, Birmaher B, Axelson D, Perepletchikova F, Brent D, Ryan N. Schedule for affective and disorders and schizophrenia for school aged children (6–18 years): Kiddie-SADS-lifetime version (K-SADS-PL DSM 5). Pittsburgh, PA: Western Psychiatric Institute and Clinic; 2016.
8. National Institute on Drug Abuse. Audit. 2013.
9. Posner K, Brown GK, Stanley B, Brent DA, Yershova KV, Oquendo MA, et al. The Columbia–Suicide Severity Rating Scale: initial validity and internal consistency findings from three multisite studies with adolescents and adults. American journal of psychiatry. 2011;168(12):1266-77.
10. Brown L, Sherbenou RJ, Johnsen SK. Test of nonverbal intelligence: TONI-4: Pro-ed Austin, TX; 2010.
11. Cohen S, Williamson G. Perceived stress in a probability sample of the United States. In: Spacapan S, & Oskamp, S., editor. The Social Psychology of Health: Claremont Symposium on Applied Social Psychology Newbury Park, CA: Sage; 1988. p. 31-67.
12. Klein EM, Brähler E, Dreier M, Reinecke L, Müller KW, Schmutzer G, et al. The German version of the Perceived Stress Scale–psychometric characteristics in a representative German community sample. BMC psychiatry. 2016;16(1):1-10.
13. Chaaya M, Osman H, Naassan G, Mahfoud Z. Validation of the Arabic version of the Cohen Perceived Stress Scale (PSS-10) among pregnant and postpartum women. BMC psychiatry. 2010;10(1):111.
14. Weiss DS, Marmar CR. The Impact of Event Scale—Revised. Assessing psychological trauma and PTSD. New York, NY, US: The Guilford Press; 1997. p. 399-411.
15. Weiss DS. The Impact of Event Scale-Revised. Assessing psychological trauma and PTSD, 2nd ed. New York, NY, US: The Guilford Press; 2004. p. 168-89.
16. Maercker A, Schützwohl M. Erfassung von psychischen Belastungsfolgen: Die Impact of Event Skala-revidierte Version (IES-R). Diagnostica. 1998.
17. Davey C, Heard R, Lennings C. Development of the A rabic versions of the I mpact of E vents S cale-R evised and the P osttraumatic G rowth I nventory to assess trauma and growth in M iddle E astern refugees in A ustralia. Clinical Psychologist. 2015;19(3):131-9.

18. Sachser C, Berliner L, Holt T, Jensen TK, Jungbluth N, Risch E, et al. International development and psychometric properties of the Child and Adolescent Trauma Screen (CATS). Journal of affective disorders. 2017;210:189-95.
19. Meiser-Stedman R, Smith P, Bryant R, Salmon K, Yule W, Dalgleish T, et al. Development and validation of the child post-traumatic cognitions inventory (CPTCI). Journal of Child Psychology and Psychiatry. 2009;50(4):432-40.
20. de Haan A, Petermann F, Meiser-Stedman R, Goldbeck L. Psychometric properties of the German version of the child post-traumatic cognitions inventory (CPTCI-GER). Child Psychiatry & Human Development. 2016;47(1):151-8.
21. Meiser-Stedman R. Post-traumatic Cognitions Inventory - Child Version (Arabic version) n. d. [Available from: https://www.childrenandwar.org/wp-content/uploads/2019/06/CPTCI_arabisch.pdf.
22. Krakow B. Nightmare Complaints in Treatment-Seeking Patients in Clinical Sleep Medicine Settings: Diagnostic and Treatment Implications. Sleep. 2006;29:1313-9.
23. Krakow B, Haynes PL, Warner TD, Santana E, Melendrez D, Johnston L, et al. Nightmares, insomnia, and sleep-disordered breathing in fire evacuees seeking treatment for posttraumatic sleep disturbance. Journal of Traumatic Stress: Official Publication of The International Society for Traumatic Stress Studies. 2004;17(3):257-68.
24. Victor SE, Klonsky ED. Validation of a brief version of the difficulties in emotion regulation scale (DERS-18) in five samples. Journal of psychopathology and Behavioral Assessment. 2016;38(4):582-9.
25. Ehring T, Svaldi J, Tuschen-Caffier B, Berking M. Validierung der Difficulties in Emotion Regulation Scale–deutsche Version (DERS-D). Unveröffentlichtes Manuskript, Universität Münster. 2013.
26. Gutzweiler R, In-Albon T. Überprüfung der Gütekriterien der deutschen Version der Difficulties in Emotion Regulation Scale in einer klinischen und einer Schülerstichprobe Jugendlicher. Zeitschrift für Klinische Psychologie und Psychotherapie. 2019;47:274-86.
27. Goodman R. Psychometric properties of the strengths and difficulties questionnaire. Journal of the American Academy of Child & Adolescent Psychiatry. 2001;40(11):1337-45.
28. Klasen H, Woerner W, Rothenberger A, Goodman R. Die deutsche Fassung des strengths and difficulties questionnaire (SDQ-Deu)-Übersicht und Bewertung erster Validierungs-und Normierungsbefunde. 2003.
29. Emam MM, Hilal MMA, Kazem AM, Alkharousi SJ. Psychometric properties of the Arabic self-report version of the strengths and difficulties questionnaire. Research in developmental disabilities. 2016;59:211-20.
30. Idzelis M, Ali AS, Gaaddasaar M, Bogoslaw LH, Ulstad K, Tanaka A. n. d. [Available from: https://www.sdqinfo.org/py/sdqinfo/b3.py?language=Somali.
31. Beck AT, Steer RA, Brown GK. Bdi-ii manual. 1996.
32. Hautzinger M, Keller F, Kühner C. Beck depressions-inventar (BDI-II): Harcourt Test Services; 2006.
33. Selmo P, Koch T, Brand J, Wagner B, Knaevelsrud C. Psychometric properties of the online Arabic versions of BDI-II, HSCL-25, and PDS. European Journal of Psychological Assessment. 2016;35:46-54.
