# Supplementary material for: START adolescents: study protocol of a randomised controlled trial to investigate the efficacy of a low-threshold group treatment programme in traumatised adolescent refugees
Source: BMJ Open. 2021 Dec 28;11(12):e057968. doi: 10.1136/bmjopen-2021-057968 (PMC8719222; doi:10.1136/bmjopen-2021-057968)
Supplement: Author's manuscript [file bmjopen-2021-057968.draft_revisions.pdf]

BMJ Open is committed to open peer review. As part of this commitment we make the peer review history of every article we publish publicly available.

When an article is published we post the peer reviewers' comments and the authors' responses online. We also post the versions of the paper that were used during peer review. These are the versions that the peer review comments apply to.

The versions of the paper that follow are the versions that were submitted during the peer review process. They are not the versions of record or the final published versions. They should not be cited or distributed as the published version of this manuscript.

BMJ Open is an open access journal and the full, final, typeset and author-corrected version of record of the manuscript is available on our site with no access controls, subscription charges or pay-per-view fees (http://bmjopen.bmj.com).

If you have any questions on BMJ Open's open peer review process please email info.bmjopen@bmj.com

BMJ Open

START Adolescents: Study protocol of a randomized controlled trial to investigate the efficacy of a low-threshold group treatment program in traumatized adolescent refugees

Journal:	*BMJ Open*
Manuscript ID	bmjopen-2021-057968
Article Type:	Protocol
Date Submitted by the Author:	02-Oct-2021

Complete List of Authors:	Sobanski, Esther; University Medical Centre of the Johannes Gutenberg University Mainz, Department of Child and Adolescent Psychiatry and Psychotherapy; Central Institute of Mental Health, Psychiatry and Psychotherapy<br>Hammerle, Florian; University Medical Centre of the Johannes Gutenberg University Mainz, Department of Child and Adolescent Psychiatry and Psychotherapy<br>Dixius, Andrea; SHG Saarland Hospital Group, Department of Child and Adolescent Psychiatry<br>Möhler, Eva; SHG Saarland Hospital Group, Child and Adolescent Psychiatry<br>Koudela-Hamila, Susanne ; Karlsruhe Institute of Technology, Department of Applied Psychology, Mental mHealth Lab<br>Ebner-Priemer, Ulrich; Karlsruhe Institute of Technology, Department of Applied Psychology, Mental mHealth Lab; Central Institute of Mental Health<br>Merz, Christian; Ruhr University Bochum, Department of Cognitive Psychology, Institute of Cognitive Neuroscience<br>In-albon, Tina; University of Koblenz Landau - Campus Landau, Department of Child and Adolescent Psychology and Psychotherapy<br>Pollitt, Brigitte; Johanniter Clinics, Child and Adolescent Psychiatry and Psychotherapy<br>Christiansen, Hanna; University of Marburg, Department of Psychology, Clinical Child and Adolescent Psychology<br>Kolar, David; University Medical Centre of the Johannes Gutenberg University Mainz, Department of Child and Adolescent Psychiatry<br>Ocker, Susanne; University Medical Centre of the Johannes Gutenberg University Mainz, Child and Adolescent Psychiatry and Pschotherapy<br>Fischer, Nicole; University Medical Centre of the Johannes Gutenberg University Mainz, Child and Adolescent Psychiatry and Pschotherapy<br>Burghaus, Ina; University of Heidelberg, Coordination Centre for Clinical Trials<br>Huss, Michael; University Medical Centre of the Johannes Gutenberg University Mainz, Department of Child and Adolescent Psychiatry
**Primary Subject Heading**:	

Secondary Subject Heading:	
Keywords:	Child & adolescent psychiatry < PSYCHIATRY, Protocols & guidelines < HEALTH SERVICES ADMINISTRATION & MANAGEMENT, PSYCHIATRY

SCHOLARONE™
Manuscripts

START Adolescents: Study protocol of a randomized controlled trial to investigate the efficacy of a low-threshold group treatment program in traumatized adolescent refugees

*Esther Sobanski[1,2] (0000-0001-7824-3959), *Florian Hammerle[1](0000-0002-2659-6266), Andrea Dixius[3], Eva Möhler[4], Susanne Koudela-Hamila[5], Ulrich Ebner-Priemer[2,5], Christian J. Merz[6], Tina In-Albon[7], Brigitte Pollitt[8], Hanna Christiansen[9], David R. Kolar[1], Susanne Ocker[1], Nicole Fischer[1], Ina Burghaus[10], Michael Huss[1]

***The first two authors contributed equally to the article**

[1]Department of Child and Adolescent Psychiatry and Psychotherapy, University Medical Center, Johannes Gutenberg University Mainz, Mainz, Germany; E-Mail: esther.sobanski@unimedizin-mainz.de; florian.hammerle@unimedizin-mainz.de; david.kolar@med.uni-muenchen.de; susanne.ocker@unimedizin-mainz.de; nicole.fischer@unimedizin-mainz.de; michael.huss@unimedizin-mainz.de;

[2]Department of Psychiatry and Psychotherapy, Central Institute of Mental Health, Medical Faculty Mannheim/Heidelberg University, Mannheim, Germany;

[3]Department of Child and Adolescent Psychiatry, SHG Clinics, 55743 Idar-Oberstein, Germany; E-Mail: a.dixius@io.shg-kliniken.de; [4]Department of Child and Adolescent Psychiatry, SHG Clinics, 66271 Kleinblittersdorf, Germany; E-Mail: e.moehler@sb.shg-kliniken.de; [5]Department of Applied Psychology, Mental mHealth Lab, Karlsruhe Institute of Technology, 76049 Karlsruhe, Germany; E-Mail: susanne.koudela@kit.edu; ulrich.ebner-priemer@kit.edu; [6]Department of Cognitive Psychology, Institute of Cognitive Neuroscience, Faculty of Psychology, Ruhr University Bochum, 44801 Bochum, Germany; E-Mail: Christian.J.Merz@ruhr-uni-bochum.de; [7]Department of Child and Adolescent Psychology and Psychotherapy, University of Koblenz-Landau, Campus Landau, 76829 Landau, Germany; E-Mail: in-albon@uni-landau.de; [8]Department of Child and Adolescent Psychiatry, Johanniter Clinics Neuwied, 56564 Neuwied, Germany; E-Mail: brigitte.pollitt@nr.johanniter-kliniken.de; [9]Department of Psychology, Clinical Child and Adolescent Psychology, University of Marburg, 35032 Marburg, Germany; E-Mail: hanna.christiansen@staff.uni-marburg.de; [10]Coordination Centre for Clinical Trials, 69120 Heidelberg, Germany; E-Mail: ina.burghaus@med.uni-heidelberg.de;

Corresponding author: Esther Sobanski; E-mail: esther.sobanski@unimedizin-mainz.de; Address: Department of Child and Adolescent Psychiatry and Psychotherapy, University Medical Center, Johannes Gutenberg University Mainz, Langenbeckstr. 1, 55131 Mainz, Germany; Phone: +49 6131 17-3285; fax: +49 6131 17-5580

For peer review only

Abstract

Introduction: No evaluated therapeutic approaches, that can efficiently be established in routine mental health care, are currently available for traumatized adolescent refugees in Germany. This study evaluates the efficacy of the **S**tress-**T**raumasymptoms-**A**rousal-**R**egulation-**T**reatment (**START**) program to reduce trauma-related symptoms and psychological distress in traumatized adolescent refugees based in Germany.

Methods and analysis: This randomized, waiting-list-controlled, multi-center trial with a 12-week follow-up will include 174 refugee minors with partial or full posttraumatic stress disorder who are fluent in either Arabic, Dari, English, German or Somali. Eligible refugee minors will be randomized to the START or waiting-list control groups. The manualized 8-weeks START program is based on techniques of dialectical behavior therapy, fosters adaptive coping with emotional distress and traumatic symptoms and comprises eight therapy modules and a booster session. Study assessments are planned at baseline, post-treatment (i. e., after program participation or waiting time), booster session at week 12 or 12-week waiting time, and at the 12-week follow-up. Primary and co-primary outcomes are changes in psychological distress and traumatic symptoms at post-treatment and will be analyzed as response variables in linear mixed regression models. Secondary outcomes are changes in further trauma-related and other psychopathological symptoms, emotion regulation, and intermediate effects of the program at follow-up. We will also assess effects of the program with ecological momentary assessments and on neuroendocrine stress parameters using hair cortisol.

Ethics and dissemination: This study has been approved by the lead ethics committee of Rhineland-Palatinate and the ethics committees of participating sites.

For peer review only - http://bmjopen.bmj.com/site/about/guidelines.xhtml

The study results will be disseminated through peer-reviewed publications and scientific conferences.

Discussion: If found to be effective, the START program will provide a low-threshold psychotherapeutic group intervention that can be applied to traumatized adolescent refugees in a variety of settings.

Registration details: German Clinical Trials Register (reference number: DRKS00020771), 21 April 2020

Strengths and limitations of this study

- Randomized waiting-list controlled multicenter trial with follow-up.
- Manualized DBT-based psychotherapeutic program with patient material available in Arabic, Dari, English, German, and Somali.
- Multimodal assessment approach of intervention effects on symptoms, functioning and daily living outcomes by psychometric evaluations, electronic ambulatory assessments (e-diaries), and neuroendocrine stress parameters.
- Restriction of study participation to refugee minors with language skills in Arabic, Dari, English, German, and Somali.

For peer review only

For peer review only - http://bmjopen.bmj.com/site/about/guidelines.xhtml

Background and rationale

Among the forcibly displaced persons worldwide there is a high number of children and adolescents under the age of 18 years (1). At the peak of the refugee influx in Germany in 2016, 261.386 of the 745.545 asylum applications recorded in total were filed from refugees under the age of 18 of which 49.786 applications were filed from unaccompanied refugee minors in the care of youth welfare services and without the company of parents or persons with the right of custody. The high percentage of asylum applications filed by refugee minors has remained stable since then though due to political changes the total number of refugees entering Germany has steadily decreased (2, 3). Of all refugees, children and adolescents, and especially unaccompanied refugee minors, are the most vulnerable group. They are at particular risk of traumatization before, during, and after their flight as well as for mental health problems (4-6). Children and adolescents seeking asylum in Germany report an average of eight potentially traumatic experiences, with 98% of unaccompanied refugee minors reporting traumatic events (5-7). They are exposed to stress due to insecure legal status and adaptation to new environments, cultural demands, and language, the loss of stability provided by their cultural background of origin as well as provided by their families and caregivers if unaccompanied (8). In addition to their often prevalent traumatization, they are also confronted with age-related developmental tasks like peer group integration, identity formation, education, professional training, and finding their role in the host society. Cross-sectional studies and reviews assessing mental health problems in refugee minors report prevalence rates of 17-71% for posttraumatic stress disorder (PTSD), 12-44% for depressive disorders, 18-38% for anxiety disorders, and 33-72% for behavior problems (9, 10). Longitudinal studies suggest a high risk of chronic mental health problems and indicate that baseline symptom severity, sex, being unaccompanied and post-migration factors like asylum

For peer review only - http://bmjopen.bmj.com/site/about/guidelines.xhtml

status and access to mental health care are predictors of future mental health status (6, 11). Studies evaluating trauma-related therapy programs in refugee minors have mostly reported significant effects on trauma and/or additional mental health symptoms. However, conclusive interpretation of the findings is limited by heterogeneous therapy approaches, lack of manualized programs and methodological shortcomings like lack of power analyses, randomization and control groups, small sample sizes or heterogeneous study settings such as clinical, school or community settings in high-income countries, but also refugee camps in war regions (12-17). Evidence from rigorous methodological approaches like randomized controlled trials (RCT) is needed to cross-validate the available results in terms of validity, reliability, and generalizability of the programs.

Study objectives

The manualized Stress-Traumasymptoms-Arousal-Regulation-Treatment (START) program was developed from clinical work with traumatized unaccompanied refugee minors in Germany to promote coping with traumatic distress and emotional irritability (18-20). An uncontrolled pilot study in 22 traumatized refugee minors showed positive effects on emotion regulation, adaptive strategies, self-control, distress, and good feasibility (18). A subsequent study confirmed the positive effects on adaptive emotion regulation strategies (21).

The primary aim of this RCT is to evaluate the efficacy of the START program in reducing psychological distress and trauma symptoms in traumatized adolescent refugees compared to a waiting-list control group (WL). Main secondary aims are to assess whether treatment effects will remain stable for at least 12 weeks after program termination (follow-up at week 24) and to assess whether the participants of the START groups improve emotion regulation strategies and mental health compared to WL. As

For peer review only - http://bmjopen.bmj.com/site/about/guidelines.xhtml

For peer review only

stress and emotions are context-dependent and highly dynamic (22), intervention effects are assessed by psychometric instruments and with electronic ambulatory assessment (e-diaries). Ambulatory assessment aims to reduce retrospective biases while gathering ecologically valid data from everyday life near real-time. It has been shown as superior to retrospective psychometric assessment in terms of predicting symptom and treatment outcome (23-26) and assessing reliably everyday functioning (27). We also evaluate intervention effects on the neuroendocrine stress system by assessing hair cortisol, which reflects the cumulative cortisol release over the past months and has been shown to capture treatment effects in PTSD (28).

The study is part of the START research consortium, which evaluates distress-reducing psychotherapeutic group and preventive family interventions in traumatized toddler, adolescent, and young adult refugees and aims to contribute to improved evidence-based therapeutic interventions for refugees within a stepped mental health care approach. The consortium comprises the START Adolescent Study, the START Childcare Study (29), the START Young Adults Study, and the smartphone-based experience sampling study tracking symptoms and daily living functioning with the use of e-diaries across the START Adolescents and Childcare studies. For further information regarding the consortium, please see www.mentalhealth4refugees.de. The presented paper reports on the START Adolescents protocol (Version 4.0, March 16th, 2020) and has been conceived according to the SPIRIT guidelines (30, 31). Please also refer to SPIRIT checklist, additional information, and to table 1.

Methods and analysis

Patient and public involvement

The original START program was developed from clinical work with highly traumatized unaccompanied refugee minors of different countries of origin, who had recently

For peer review only - http://bmjopen.bmj.com/site/about/guidelines.xhtml

arrived in Germany (18-20). The adapted and extended version of the program, that is evaluated in this RCT, was redesigned according to the participants' feedback of the pilot study groups, who were also traumatized refugee minors from different countries of origin. The public was not involved when designing the study.

Trial design and setting

The study is conducted as a 12-weeks, two-arm, randomized waiting-list-controlled, multi-center trial with a 12-week follow-up and six participating outpatient departments in Germany, all of which are experienced in treating traumatized adolescent refugees. The study intervention comprises an adapted version of the START program (18-20). Languages used in the study for the intervention and the assessments are Arabic, Dari, English, German or Somali. Interpreters are involved if needed. The study is not blinded for the treatment condition, which will be evident to participants, caregivers, and study therapists. Key stopping rules for patients are a withdrawal of informed consent, unwillingness to further participate in the trial, any factors affecting the patient's or others' well-being, e.g. acute suicidality or acute endangerment to others, onset of other acute severe mental disorders, alcohol or substance abuse, inpatient treatment of over two days, start of concurrent psychotherapy and more than two psychotherapeutic crisis interventions. Key stopping rules for participating centers are non-adherence with the ICH-GCP (32) or the study protocol, insufficient recruitment of participants, or insufficient data quality. The key stopping rule of the trial is a change in the overall risk-benefit ratio.

Participants

We will allocate 174 participants to the trial and expect that we will have to screen 240 adolescent refugees for eligibility. Inclusion criteria are: informed consent by adolescents and caregivers, flight background, age between 13,0 and 17,11 years,

For peer review only - http://bmjopen.bmj.com/site/about/guidelines.xhtml

verbal communication skills and reading comprehension in one of the study languages, non-verbal IQ ≥ 70, partial or full PTSD, PTSD symptoms are the clinically most impairing condition with at least moderate clinical symptom severity. We kept the exclusion criteria to a minimum, to foster the applicability of the program in routine care as much as possible. Exclusion criteria are: current substance use disorder and/or harmful alcohol abuse, primary severe mental disorders other than PTSD, acute suicidality or danger to others, unstable psychotropic medication (change of psychotropic medication within the last two weeks before baseline assessment), current ongoing psychotherapy, inpatient status or study participation in another clinical trial, unaccompanied refugee minor without a legal representative, known pregnancy. Please see for detailed information about the assessment of eligibility criteria also the sections “screening” and “psychometric instruments”. The participants’ study flow is provided in figure 1.

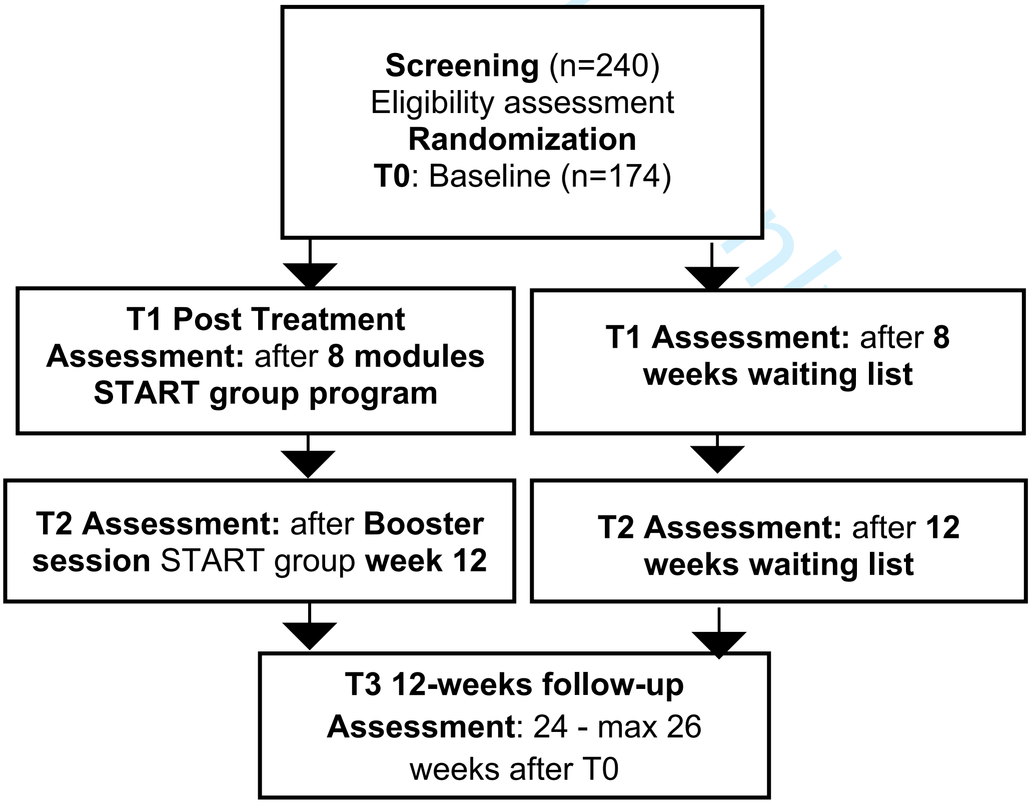

Figure 1: Participants‘ study flow

For peer review only - http://bmjopen.bmj.com/site/about/guidelines.xhtml

Study conduct

Recruitment

For recruitment, study information is provided to schools, child and adolescent psychotherapists/psychiatrists, social workers, youth welfare providers, at conferences, professional working group meetings, in newspaper articles or interviews, on the institutions' homepages, and on social media. All participants' information about the study is provided in a caregiver and adolescents' version in all study languages.

Trial flow

For a summary of trial flow and assessment visits please see the SPIRIT flow diagram, table 1. All assessments are provided by trained study staff and are performed at the screening visit, baseline (T0), post-treatment (after 8-week START program or 8-week waiting time; T1), after the booster session or 12-week waiting time (T2), and after 24 weeks at the 12-week follow-up (T3). The maximum time allowed between T0 and T3 is 26 weeks, and the total time needed to complete the study is approximately 48 months. After termination of the study, all participants are informed about their current diagnostic status and available interventions provided by child and adolescent psychiatric and psychotherapeutic routine care. Participants of the WL are given the opportunity to take part in independent START groups.

Screening visit: Assessment of informed consent for study participation, inclusion and exclusion criteria, demographic and medical data. Inclusion of eligible participants, randomization to the intervention (START) or WL using a web-based randomization system with permuted blocks stratified by center (http://randomizer.at).

Baseline (T0), post-treatment (T1), booster session (T2), 12-week follow-up (T3) assessments: T0 takes place between one and two weeks after randomization (WL) or before the first program session (START), T1 within one week after the 8-week

For peer review only - http://bmjopen.bmj.com/site/about/guidelines.xhtml

program (START) or within 8-9 weeks after T0 (WL), T2 within one week after the booster session (START) or 4-5 weeks after T1 (WL) and T3 follow-up visits within 12 weeks after T2 (START/WL). At T0 only, the Post-Migration Living Difficulties Questionnaire (PMLD) and Questionnaire on Therapy Expectation are assessed. At the T0(T1)-T3 visits primary endpoints, trauma symptoms, emotion regulation, general psychopathology, overall clinical severity of mental disorders, suicidality and endangerment to others are assessed and one week e-diaries are applied. Hair cortisol is assessed at T0, T2 and T3. Adverse events are assessed at T0-T3 visits and weekly until T1.

	STUDY PERIOD				
**TIME POINT**	**Screening**	**T0 Study visit Baseline** One week after random. (WL) or before START Program	**T1 Study visit** 8 weeks after T0 (WL) or after START Program	**T2 Study visit** 4 weeks after T1 (WL) or at booster session (START)	**T3 Study visit Follow-up** 12 weeks after T2
**ENROLMENT**					
Eligibility screen	X				
Informed consent	X				
Randomization	X				
**ASSESSMENTS**					
Demographic data	x				
Medical history	x				
Medication/ psychotherapy	x	x	x	x	x
Saliva drug screening	x				
TONI-4	x				
ETI-CA	x		x	x	x
K-SADS-PL	x				
CGI-S	x		x	x	x
C-SSRS	x		x	x	x
REB[1]		**Weekly**			
AUDIT	x				
Endangerm. to others	x		x	x	x
PSS-10	x	x	x	x	x
IES-R	x	x	x	x	x
SDQ self		x	x	x	x
SDQ caregiver	x		x	x	x
BDI-II		x	x	x	x

For peer review only - http://bmjopen.bmj.com/site/about/guidelines.xhtml

DERS18		X	X	X	X
cPTCI		X	X	X	X
CATS-2		X	X	X	X
DDNSI		X	X	X	X
PMLD		X			
Therapy expectation		X			
E-Diaries		X	X	X	X
Hair cortisol		X		X	X
AE assessments		X	X	X	X
**INTERVENTION**					
START program		→	→		
Booster session				◆	

Table 1: SPIRIT Flow Diagram

[1]First assessment at T0 then weekly until T3; AE: Adverse events; WL: Waiting-list group. Instruments: Alcohol Use Disorders Identification Test (AUDIT); Beck Depression Inventory, second edition (BDI-II); Child and Adolescent Trauma Screen-2 (CATS-2); Clinical Global Impression Scale, severity of illness subscale (CGI-S); Columbia-Suicide Severity Rating Scale (C-SSRS); Disturbing Dream and Nightmare Severity Index (DDNSI); Difficulties in Emotion Regulation Scale, 18-item version (DERS-18); Essen Trauma Inventory for Children and Adolescents (ETI-CA); Impact of Event Scale-Revised (IES-R); Perceived Stress Scale, 10-item version (PSS-10); Posttraumatic Cognitions Inventory - child version (cPTCI); Schedule for Affective Disorders and Schizophrenia for School-Age Children-Present and Lifetime version (K-SADS-PL); Strengths and Difficulties Questionnaire (SDQ); Test of Nonverbal Intelligence, Fourth Edition (TONI 4)

Intervention

Details of the START Adolescents program are provided in table 2. START Adolescents study groups comprise three to eight participants and are restricted to study participants, who have been randomized to the intervention group. The manualized START group program is based on techniques of dialectical behavioral therapy with emphasis on coping with emotional distress and traumatic symptoms, resilience, adaptive functioning, and self-efficacy, but not on confrontation with traumatic memories. The group therapy is provided by two therapists through twice-weekly 60-minute sessions and interactions are based on validation and acceptance techniques. Written participant program materials are available in all study languages and if necessary, translators will attend the group therapies.

For peer review only - http://bmjopen.bmj.com/site/about/guidelines.xhtml

The original START Adolescents program comprised five modules with psychoeducation on trauma, stress, distressing emotions and crises, mindfulness training, arousal regulation, stress reduction techniques, and handling nightmares. For the current study, the START manual (18-20) was extended by the three additional modules 6-8 and a booster session. The additional modules provide an intensified training of stress reduction skills, mindfulness techniques, and additional psychoeducation about emotions and interpersonal effectiveness. Each session follows a defined structure and comprises, as fixed repeated elements, mindfulness training, monitoring one's own inner tension/stress ("stress signal light"), reviewing experiences when applying the program skills, new contents/psychoeducation, and skills training. The final session in module 8 is dedicated to a farewell ceremony and to validating and rewarding the participants' efforts and achievements. During the booster session, which is scheduled four weeks after program termination, participants review their experiences when applying the acquired skills and techniques, their open needs, and provide an outlook on their personal future wishes and goals. If standardized risk assessments or participants' personal information reveal acute danger to the self or others, additional psychotherapeutic crisis interventions will be offered to the participant. Participants with ongoing acute suicidal tendencies or ongoing danger to others after two subsequent crisis interventions must be excluded from study participation and transferred to specific intense psychiatric care.

For peer review only

**Before beginning of START**: Written patient information. Handout.<br>**Every session**: Mindfulness practice and stress signal light. Discussing between-session skills practice. Written patient material.<br>**Between the sessions:** Participants are asked to train skills and techniques.		
	**Key interventions**	**Practice / Training techniques**
**Module 1**<br>Week 1<br>Sessions 1 & 2	***Welcome.*** Introduction to START.<br>Introduction of participants.<br>***Psychoeducation.*** Explanation of the concepts stress, high stress and stress-regulation skills.	High-stress skills.<br>Progressive muscle relaxation.

	Teaching techniques. Skills for reducing distress and high distress. Relaxation techniques.	
**Module 2**<br>Week 2<br>Sessions 3 & 4	***Consolidation:*** Available skills.<br>***Teaching techniques.*** Stress signal light for stress / inner tension monitoring and awareness. Emotion regulation strategies: mindful senses (hearing, feeling, tasting, watching, smelling). Skills box.	Mindful sensual experiences.<br>Individual stress signal lights.<br>Individual stimuli for distress/ euthymia.<br>High-stress skills.<br>Individual skills boxes.
**Module 3**<br>Week 3<br>Sessions 5 & 6	***Consolidation.*** Individual skills boxes.<br>***Teaching techniques.*** Skills chains. Individual red light. Self-empowerment / resilience.	Skills chains<br>Self-empowerment
**Module 4**<br>Week 4<br>Sessions 7 & 8	***Consolidation.*** Available skills.<br>***Psychoeducation.*** Explanation of the concept of crisis and management of crisis.<br>***Teaching techniques.*** Managing crisis: acceptance / skills / safe place. Managing flashbacks. Safety and emergency card. How to recognize change. How to change the moment?	Recognition and managing of crisis.<br>Individual safe place.<br>Skills that change the moment.
**Module 5**<br>**Week 5**<br>Sessions 9 & 10	***Consolidation.*** Available skills.<br>***Psychoeducation.*** Sleep disturbances and nightmares.<br>***Teaching techniques.*** Managing nightmares.	Telling / writing nightmares.<br>Skills for managing nightmares.
**Module 6**<br>Week 6<br>Sessions 11 & 12	***Consolidation.*** Available skills.<br>***Psychoeducation.*** Emotions and emotion awareness.<br>***Teaching techniques***. Emotion surfing "Letting go of emotional suffering"	Recognizing and naming emotions (emotion cards).<br>Individual pleasant and unpleasant emotions.<br>Emotion surfing.
**Module 7**<br>Week 7<br>Sessions 13 & 14	***Consolidation.*** Available skills, emotion recognition and understanding.<br>***Psychoeducation.*** Emotional network: Interdependence of emotions, thinking, behavior and prompting stimuli. Skills for emotion regulation.	Role-play/ competition: Recognizing emotions and thoughts.<br>Understand, name and regulate emotions and related behavior and thoughts.
**Module 8**<br>Week 8<br>Sessions 15 & 16	**Review and consolidation:** Available skills, current difficulties.<br>**Crisis plan.**<br>**Close-Out.** Farewell celebration.<br>**Awarding the participants*.* Certificates**: "You did your very best."<br>**Outlook to booster session.**	Experiencing acquired / activated resources (balloon game).<br>Discussing individual skills lists, open needs.<br>Discussing crisis plan / designing emergency cards.
**Booster Session**<br>Week 12<br>Session 17	**Review and consolidation:** Helpful skills, difficulties with skills practice within the last four weeks, current open needs.<br>**Small symbolic gift,** e.g. little gemstone	Outlook for future / participants' wishes / practicing how "to turn the tide".

Table 2: START Adolescents Program, modules 1-8 and booster session

For peer review only - http://bmjopen.bmj.com/site/about/guidelines.xhtml

Assessments

Psychometric instruments

For details of the instruments including psychometric properties please see additional information. All self-report assessments are applied in the study languages, all interviews are applied with the help of interpreters if indicated.

***Inclusion and exclusion criteria**:* In- and exclusion criteria are assessed with the Essen Trauma Inventory for Children and Adolescents (ETI-CA; (33)), Clinical Global Impression Scale, severity of illness subscale (CGI-S; (34)), the Kiddie-Schedule for Affective Disorders and Schizophrenia for School-Age Children-Present and Lifetime version (K-SADS-PL; (35-37)), the Alcohol Use Disorders Identification Test (AUDIT; (38)), the Columbia-Suicide Severity Rating Scale (C-SSRS; (39)), the Test of Nonverbal Intelligence, Fourth Edition (TONI 4; (40)), and the standardized Questionnaire to Assess Endangerment to Others (39).

Primary endpoints: Psychological distress is assessed with the Perceived Stress Scale, 10-item version (PSS-10), a validated self-report questionnaire, that measures the intensity with which individuals appraise their daily life as stressful, unpredictable, uncontrollable, and overloaded. Reliability and validity for the Arabic, English and German version have been shown to be sufficient or good (41-44). Traumatic symptoms are assessed with the Impact of Event Scale-Revised (IES-R), a validated, self-report questionnaire evaluating traumatic symptoms on the subscales intrusion, avoidance, hyperarousal and on an overall measure of traumatic distress. Reliability and validity for the Arabic, English, and German version have been shown to be good (45-48).

Secondary endpoints: We assess trauma-related psychosocial impairment and DSM-5 PTSD criteria with the Child and Adolescent Trauma Screen-2 (CATS-2; (49)), trauma-related cognitions with the Posttraumatic Cognitions Inventory - child version

For peer review only - http://bmjopen.bmj.com/site/about/guidelines.xhtml

(cPTCI; (50-52)) and frequency and intensity of nightmares with the Disturbing Dream and Nightmare Severity Index (DDNSI; (53, 54)). Emotion regulation is assessed with the 18-item version of the Difficulties in Emotion Regulation Scale (DERS-18; (55)), general psychopathology with the Strengths and Difficulties Questionnaire (SDQ; (56, 57)), and the Beck Depression Inventory, second edition (BDI-II; (58, 59)). Overall severity of illness is assessed with the CGI-S (34).

Additional psychometric assessments: We assess adverse life experiences related to migration with the adapted German version of the self-rated Post-Migration Living Difficulties Questionnaire (PMLD; (60, 61)), and therapy expectations with a self-designed questionnaire comprising three questions that are answered on a 5-point Likert-type scale.

Ambulatory momentary assessment: electronic diaries

We use smartphone-based electronic (e)-diaries to assess aversive inner tension, emotional intensity, trauma symptoms (intrusions, hypervigilance), cognitive and behavioral avoidance of triggering situations in daily environments, sleep quality, stressfulness, content and number of nightmares, self-efficacy, self-esteem, rumination and somatization (62-64). Filling in the answers takes a few minutes and items are available in all study languages. Rumination about cultural differences, social support and conflicts, discrimination experiences, and personally important events at school will be used as context variables to determine their associations with emotional distress and trauma symptoms. Participants receive a comprehensive explanation of the use of e-diaries as a spoken PowerPoint presentation and a written document in each participant's respective language. After the presentation, the participant is asked to do a test trial on the smartphone. Smartphones are programmed with the e-diary

For peer review only - http://bmjopen.bmj.com/site/about/guidelines.xhtml

app movisensXS (65) and employ an hourly one-week time-based e-diary design. Prompts are pseudo-randomized within a time frame of 50-70 minutes to avoid expectancy effects. At the beginning of the study, participants are asked to enter their school schedules and determine their first prompt in the morning. On the weekends, e-diary assessments start at 10 a.m., no data are assessed during school hours. The prompts end at about 9 p.m. on school days and about 10 p.m. if there is no school the next day.

Hair cortisol

If additional informed consent for hair cortisol collection is provided by study participants and their legal representatives, we collect thin hair strands from the posterior vertex of the head cut as close to the scalp as possible. Hair strands are tied together, sealed, and stored in a dry and dark place. Prior to analyses, they will be cut into a 2cm segment proximal to the scalp, representing cumulative cortisol secretion of the last two months (66).

Quality assurance and monitoring

The study procedures are monitored by the Coordination Center for Clinical Trials (KKS) Heidelberg according to the ICH-GCP (32) with respect to a risk-based quality management strategy and ensure that the trial is conducted according to protocol and regulatory requirements. All data of the ongoing study are reviewed by an independent data monitoring committee once a year with special focus on safety issues.

Manual adherence across different therapists and participating centers is ensured by training all therapists on the START Adolescents program and regular supervision with standardized assessments of manual adherence. We require a certain level of

For peer review only

professional training from the therapists, of whom at least one must be a graduated psychologist, pedagogue, or physician.

The quality of psychometric assessments, tests, and interviews is ensured by training the raters on the study assessments. Raters must have a Bachelor's or Master's degree in psychology or a comparable standard. Raters with a Bachelor's degree in psychology will administer questionnaires and psychological tests, raters with a Master's degree in psychology will administer the study interviews.

Data management

The KKS Heidelberg is responsible for data management and analysis as well as security as well as data transfer processes. All procedures are implemented in accordance with ICH-GCP guidelines and the Declaration of Helsinki (32, 67). The protection of private data is ensured by a pseudonymization procedure, and all private data are handled with respect to the European General Data Protection Regulation (68).

Data are assessed by an eCRF and paper-based self-report questionnaires. The latter are transferred in copy to the KKS Heidelberg for double entry into the eCRF. E-diary data are transferred to the Mental mHealth lab, Karlsruhe Institute of Technology (KIT), analyzed there and saved in encrypted form. The smartphones for e-diaries are provided by the research facility; no private smartphones are used for study purposes.

- **Data availability**

The research data generated during this study will be available upon reasonable request by the study coordinating center at the Department of Child and Adolescent Psychiatry, University Medical Center, Mainz, Germany. Anonymized data use by other researchers not involved in the study may be done with prior agreement.

For peer review only - http://bmjopen.bmj.com/site/about/guidelines.xhtml

Statistics

Sample size calculation

The sample size calculation is based on the co-primary outcomes of emotional distress (PSS-10) and traumatic symptoms (IES-R) at T1. Based on available studies (69, 70) we assume in our study an effect size of *d*=0.63 for the PSS-10 and of *d*=0.56 for the IES-R score at T1. This results in a required sample size *of n*=84 (PSS-10) and *n*=104 (IES-R) for a one-sided test at an *α*=.025 level with a power of 80% to detect such differences. The START pilot trial showed a drop-out rate <10% and was conducted as a one-center single-arm naturalistic trial with mostly inpatients (20). We, therefore, calculated the sample size on the assumption of 40% drop-outs between T0 and T1 to account for the multi-center, waiting-list-controlled approach and unstable living conditions of the included population. This amounts to *n*=174 participants who need to be randomized in our trial.

Analysis population and analysis

All randomized participants will be included in the full-analysis set as allocated. All randomized participants, who finish the study according to the study protocol until T2 as planned with no missing values for T1 (primary endpoint) will be included in the per-protocol set for analysis as allocated. All randomized participants will be included in the safety population according to the applied intervention.

Baseline characteristics will be analyzed descriptively for the safety, full-analysis, and per-protocol set in the intervention and control group. The primary and co-primary endpoints will be analyzed in the full-analysis set. Missing values will be imputed. Endpoints will be assessed as response variables in linear mixed regression models with site and psychotherapeutic group as random effects, intervention group, sex, and region of origin as fixed effects, and age, PSS-10, and IES-R baseline scores,

For peer review only - http://bmjopen.bmj.com/site/about/guidelines.xhtml

respectively, as linear effects with a hierarchical testing procedure that maintains the overall significance level of α=.025. The primary analysis will be repeated in the per-protocol population as a sensitivity analysis. Since the different timing of the T0 baseline examination in the study groups after randomization could cause biases in the effect estimates, that cannot be estimated or modeled, additional sensitivity analyses will be performed, such as repeating the primary analysis using the screening value instead of the baseline value. Secondary outcomes will be analyzed in the full-analysis set. Missing values will not be imputed. The stability of treatment effects up to T3 will be assessed by calculating confidence intervals on the T1-T3 and T2-T3 difference for the PSS-10 and IES-R total scores in the intervention group. Psychometric scales used for assessment of secondary outcomes and hair cortisol concentrations will be analyzed according to primary endpoints or descriptively. For the analysis of e-diary data, multilevel models will be used to characterize momentary mechanisms and context-dependent affective experiences, which allow a nested data structure (momentary experiences within participants), and enable different numbers of ratings per participant to be handled quite well (71). Safety variables will be analyzed in the safety and full-analysis population.

Ethics and dissemination

The study protocol, patient recruitment procedures, patients' information, and informed consent material have been approved by the lead ethics committee of Rhineland-Palatinate (ID 2019-14709) and the ethics committees of participating sites. For every substantial protocol modification approval of all ethics committees is required. The study will be conducted according to ICH-GCP (32) and the Declaration of Helsinki (67) and has been registered at the German Clinical Trials Register (ID:

For peer review only - http://bmjopen.bmj.com/site/about/guidelines.xhtml

DRKS00020771). Study results will be published through peer-reviewed publications and presented at scientific and clinical conferences.

Trial status

Recruitment started 1 October 2020.

Discussion

Evidence-based, evaluated, low-threshold, and culture-sensitive psychotherapeutic treatment programs, that reduce distress caused by traumatic experiences that can be efficiently established in routine mental health care, would represent valuable therapeutic interventions to support refugee minors with trauma-related symptoms and emotion regulation difficulties. However, such interventions are currently lacking. The primary aim of this study is to evaluate the clinical efficacy and intermediate outcome of the distress-reducing psychotherapeutic START program and to contribute to improved evidence-based therapeutic interventions for adolescent refugees within a stepped-care mental health approach in Germany. If shown to be effective, the first rigorously evaluated manualized intervention program for traumatized adolescent refugees will be available, which will have widespread implications for clinical practice. Some limitations for the application of the program in mental health routine care in host countries may be that the therapeutic study groups are limited only to traumatized refugees, which may not reflect clinical reality. Another limitation is the restriction of study participation to refugee minors with language skills in Arabic, Dari, English, German, and Somali. Thus, the study will not provide information on the effectiveness of the program if applied to refugee minors with a different cultural or language background. However, since all program materials are available in Arabic, Dari, English, German, and Somali, the START program will provide a treatment option not

For peer review only

only in German- or English-speaking host countries that care for refugee minors but also in refugee camps based in Arabic-, Dari- or Somali-speaking countries, where is a huge need for easy-to-apply psychotherapeutic treatment options.

Beyond effects on the symptom level, we will analyze intervention effects on psychosocial functioning and everyday behavior through the use of e-diaries and on the neuroendocrine stress system by analyzing hair cortisol. The multimodal assessment approach of symptoms, functioning, daily living outcomes, context variables, and neuroendocrine effects, as well as the follow-up assessment, will enable us to analyze immediate and intermediate treatment effects not only on the phenomenological clinical level but also on the biological and daily living level. This will enable us to gain a greater understanding of intervention effects, influencing factors and the course of trauma-related mental health problems in refugee minors.

Contributors

ES obtained the funding for the START adolescent project and the Consortium coordination, designed the study design for the trial and the structure of START Consortium. ES and FH drafted the first version of the manuscript and revised the manuscript several times. FH organizes the START Adolescents study. AD and EM developed the START intervention, were involved in the adaptation of the program for this study and revised the manuscript. AD heads the Idar-Oberstein site. EM heads the Kleinblittersdorf site. SKH and UEP designed the smartphone-based experience sampling study and critically revised the manuscript. UEP obtained funding for the experience sampling study and is the head Mental mHealth lab, KIT. CJM designed the protocol for the assessment of neuroendocrine parameters and critically revised the manuscript. TIA is the head of the Landau site and critically revised the manuscript. BP is the head of the Koblenz site and critically revised the manuscript. HC is the head of the Marburg site and critically revised the manuscript. DK was, SO and NF are involved in the coordination of this study and DK, SO and NF critically revised the manuscript. IB is responsible for biostatistics and critically revised the manuscript. MH coordinates the START consortium and was involved in critically revising the manuscript. All authors read and approved the final manuscript.

Funding and sponsor: With the exception of the hair cortisol assessments, which will be self-funded by the Department of Child and Adolescent Psychiatry and Psychotherapy, University Medical Center Mainz, Germany, the START Adolescents study is funded by the German Federal Ministry of Education and Research (BMBF); funding code: 01EF1807A. The trial sponsor is the University Medical Center Mainz, Langenbeckstraße 1 55131 Mainz, Germany, www.unimedizin-mainz.de. The sponsor played no part in the study design and writing this report.

For peer review only - http://bmjopen.bmj.com/site/about/guidelines.xhtml

Competing interests: All authors have completed the ICMJE uniform disclosure form. The first two authors, Esther Sobanski & Florian Hammerle, received article processing fees and travel grants for conferences from the Federal Ministry of Education and Research, Germany and are consultants of the Federal Ministry of Rhineland Palatinate for refugee issues. Esther Sobanski recieved funding for the START Adolescents study from the Federal Ministry of Education and Research, Germany. Tina In-Albon received a research grant from the Federal Ministry of Education and Research for research of non-suicidal self-injury in adolescents and is president of the German Society of Cognitive Behavioral Psychotherapy for Children and Adolescents (KJPVT e.V.), unpaid position. David R. Kolar received travel grants for conferences from the Federal Ministry of Education and Research, Germany. Michael Huss recieved funding for the START Adolescents study from the Federal Ministry of Education and Research, Germany. All other authors declare that they have no competing interests.

Provenance and peer review: Study protocol was externally peer reviewed.

Patient consent for publication: Not applicable.

Acknowledgements: We would like to acknowledge Sarah Mannion, MA (Cantab) for reviewing the English, Bianca Brückner for the help with the recruitment, and Christa Hellmann for the technical help when preparing the manuscript.

References

1. United Nations High Commissioner for Refugees. Global Trends Forced Displacement in 2019. Denmark: UNHCR; 2020.
2. Office for Migration and Refugees. Schlüsselzahlen Asyl 2019. Nürnberg: Bundesamt für Migration und Flüchtlinge; 2020.
3. Office for Migration and Refugees. Flüchtlinge (2019): Aktuelle Zahlen. 2019.
4. Höhne E, Swantje van der Meer A, Kamp-Becker I, Christiansen H. A systematic review of risk and protective factors of mental health in unaccompanied minor refugees. Eur Child Adolesc Psychiatry. 2020.
5. Müller LRF, Gossmann K, Hartmann F, Büter KP, Rosner R, Unterhitzenberger J. 1-year follow-up of the mental health and stress factors in asylum-seeking children and adolescents resettled in Germany. BMC public health. 2019;19(908):1-11.
6. Müller LRF, Büter KP, Rosner R, Unterhitzenberger J. Mental health and associated stress factors in accompanied and unaccompanied refugee minors resettled in Germany: a cross-sectional study. Child and adolescent psychiatry and mental health. 2019;13(1):1-13.
7. Walg M, Fink E, Großmeier M, Temprano M, Hapfelmeier G. Häufigkeit psychischer Störungen bei unbegleiteten minderjährigen Flüchtlingen in Deutschland. Zeitschrift für Kinder- und Jugendpsychiatrie und Psychotherapie. 2016;45:58-68.
8. Anders M, & Christiansen, H. . Die Versorgung unbegleiteter minderjähriger Flüchtlinge – eine systematische Literaturübersicht zu psychologischen Interventionen. Kindheit und Entwicklung. 2016;25(4):216-30.
9. Sierau S, Schneider E, Nesterko Y, von Klitzing K, Glaesmer H. Psychische Belastung bei unbegleiteten jungen Geflüchteten in Jugendhilfeeinrichtungen. Psychiatrische Praxis. 2019;46(03):135-40.
10. Kien C, Sommer I, Faustmann A, Gibson L, Schneider M, Krczal E, et al. Prevalence of mental disorders in young refugees and asylum seekers in European Countries: a systematic review. European child & adolescent psychiatry. 2019;28(10):1295-310.
11. Tam SY, Houlihan S, Melendez-Torres G. A systematic review of longitudinal risk and protective factors and correlates for posttraumatic stress and its natural history in forcibly displaced children. Trauma, Violence, & Abuse. 2017;18(4):377-95.
12. Barron IG, Abdallah G, Smith P. Randomized control trial of a CBT trauma recovery program in Palestinian schools. Journal of Loss and Trauma. 2013;18(4):306-21.
13. Tyrer RA, Fazel M. School and community-based interventions for refugee and asylum seeking children: a systematic review. PloS one. 2014;9(2):e89359.

For peer review only - http://bmjopen.bmj.com/site/about/guidelines.xhtml

14. Pfeiffer E, Goldbeck L. Evaluation of a trauma-focused group intervention for unaccompanied young refugees: A pilot study. Journal of Traumatic Stress. 2017;30(5):531-6.
15. Gormez V, Kılıç HN, Orengul AC, Demir MN, Mert EB, Makhlouta B, et al. Evaluation of a school-based, teacher-delivered psychological intervention group program for trauma-affected Syrian refugee children in Istanbul, Turkey. Psychiatry and Clinical Psychopharmacology. 2017;27(2):125-31.
16. Nocon A, Eberle-Sejari R, Unterhitzenberger J, Rosner R. The effectiveness of psychosocial interventions in war-traumatized refugee and internally displaced minors: systematic review and meta-analysis. European journal of psychotraumatology. 2017;8(sup2):1388709.
17. Demazure G, Gaultier S, Pinsault N. Dealing with difference: a scoping review of psychotherapeutic interventions with unaccompanied refugee minors. European child & adolescent psychiatry. 2018;27(4):447-66.
18. Dixius A, Möhler E. START–Entwicklung einer Intervention zur Erststabilisierung und Arousal-Modulation für stark belastete minderjährige Flüchtlinge. Praxis der Kinderpsychologie und Kinderpsychiatrie. 2017;66(4):277-86.
19. Dixius A, Möhler E. Ein neues Therapie-Konzept validiert die besonderen Bedürfnisse geflüchteter Kinder und Jugendlicher: START. Psychotherapie Forum. 2017;22(3):76-85.
20. Dixius A, Stevens A, Moehler E. A pilot evaluation study of an intercultural treatment program for stabilization and arousal modulation for intensely stressed children and adolescents and minor refugees, called START (Stress-Traumasymptoms-Arousal-Regulation-Treatment). ARC Journal of Psychiatry. 2017;2(2):7-24.
21. Dixius A, Möhler E. Feasibility and Effectiveness of a New Short-Term Psychotherapy Concept for Adolescents With Emotional Dysregulation. Frontiers in Psychiatry. 2021;11:1630.
22. Ebner-Priemer UW, Eid M, Kleindienst N, Stabenow S, Trull TJ. Analytic strategies for understanding affective (in) stability and other dynamic processes in psychopathology. Journal of abnormal psychology. 2009;118(1):195.
23. Forbes EE, Stepp SD, Dahl RE, Ryan ND, Whalen D, Axelson DA, et al. Real-world affect and social context as predictors of treatment response in child and adolescent depression and anxiety: an ecological momentary assessment study. Journal of Child and Adolescent Psychopharmacology. 2012;22(1):37-47.
24. Peeters F, Berkhof J, Rottenberg J, Nicolson NA. Ambulatory emotional reactivity to negative daily life events predicts remission from major depressive disorder. Behaviour research and therapy. 2010;48(8):754-60.
25. Wichers M, Lothmann C, Simons CJ, Nicolson NA, Peeters F. The dynamic interplay between negative and positive emotions in daily life predicts response to treatment in depression: a momentary assessment study. British Journal of Clinical Psychology. 2012;51(2):206-22.
26. Wichers M, Peeters F, Geschwind N, Jacobs N, Simons C, Derom C, et al. Unveiling patterns of affective responses in daily life may improve outcome prediction in depression: a momentary assessment study. Journal of affective disorders. 2010;124(1-2):191-5.
27. Santangelo PS, Reinhard I, Koudela-Hamila S, Bohus M, Holtmann J, Eid M, et al. The temporal interplay of self-esteem instability and affective instability in borderline personality disorder patients' everyday lives. Journal of abnormal psychology. 2017;126(8):1057-65.
28. Steudte-Schmiedgen S, Kirschbaum C, Alexander N, Stalder T. An integrative model linking traumatization, cortisol dysregulation and posttraumatic stress disorder: Insight from recent hair cortisol findings. Neuroscience & Biobehavioral Reviews. 2016;69:124-35.
29. Mayer A, Taubner S, Bark C, Holl J. Herausforderungen in der frühpädagogischen Arbeit mit geflüchteten Familien mentalisierungsbasiert begegnen. Praxis der Kinderpsychologie und Kinderpsychiatrie. 2019;68(8):711-27.
30. Chan A-W, Tetzlaff JM, Altman DG, Dickersin K, Moher D. SPIRIT 2013: new guidance for content of clinical trial protocols. Lancet. 2013;381(9861).
31. Chan A-W, Tetzlaff JM, Altman DG, Laupacis A, Gøtzsche PC, Krleža-Jerić K, et al. SPIRIT 2013 statement: defining standard protocol items for clinical trials. Annals of internal medicine. 2013;158(3):200-7.

For peer review only - http://bmjopen.bmj.com/site/about/guidelines.xhtml

32. ICH Expert Working Group. ICH Harmonised Tripartite Guideline for good clinical practice E6 (R1). ICH Harmon Tripart Guidel. 1996;1996(4).

33. Graham FS, W, Tagay S. ETI-CA Essen Trauma-Inventory for Children and Adolescents - Interview Essen: LVR-Klinikum Essen, Universität Duisburg; 2012.

34. Guy W. Clinical global impression. Assessment manual for Psychopharmacology. 1976:217-22.

35. Kaufman J, Birmaher B, Brent D, Rao U, Flynn C, Moreci P, et al. Schedule for affective disorders and schizophrenia for school-age children-present and lifetime version (K-SADS-PL): initial reliability and validity data. Journal of the American Academy of Child & Adolescent Psychiatry. 1997;36(7):980-8.

36. American Psychiatric Association. Diagnostic and Statistical Manual of Mental Disorders. 5th ed. Washington, DC2013.

37. Kaufman J, Birmaher B, Axelson D, Perepletchikova F, Brent D, Ryan N. Schedule for affective and disorders and schizophrenia for school aged children (6–18 years): Kiddie-SADS-lifetime version (K-SADS-PL DSM 5). Pittsburgh, PA: Western Psychiatric Institute and Clinic; 2016.

38. National Institute on Drug Abuse. Audit. 2013.

39. Posner K, Brown GK, Stanley B, Brent DA, Yershova KV, Oquendo MA, et al. The Columbia–Suicide Severity Rating Scale: initial validity and internal consistency findings from three multisite studies with adolescents and adults. American journal of psychiatry. 2011;168(12):1266-77.

40. Brown L, Sherbenou RJ, Johnsen SK. Test of nonverbal intelligence: TONI-4: Pro-ed Austin, TX; 2010.

41. Cohen S, Williamson G. Perceived stress in a probability sample of the United States. In: Spacapan S, & Oskamp, S., editor. The Social Psychology of Health: Claremont Symposium on Applied Social Psychology Newbury Park, CA: Sage; 1988. p. 31-67.

42. Klein EM, Brähler E, Dreier M, Reinecke L, Müller KW, Schmutzer G, et al. The German version of the Perceived Stress Scale–psychometric characteristics in a representative German community sample. BMC psychiatry. 2016;16(1):1-10.

43. Musial F, Büssing A, Heusser P, Choi K-E, Ostermann T. Mindfulness-based stress reduction for integrative cancer care–a summary of evidence. Complementary Medicine Research. 2011;18(4):192-202.

44. Chaaya M, Osman H, Naassan G, Mahfoud Z. Validation of the Arabic version of the Cohen Perceived Stress Scale (PSS-10) among pregnant and postpartum women. BMC psychiatry. 2010;10(1):111.

45. Weiss DS, Marmar CR. The Impact of Event Scale-Revised. Assessing psychological trauma and PTSD. New York, NY, US: The Guilford Press; 1997. p. 399-411.

46. Weiss DS. The Impact of Event Scale-Revised. Assessing psychological trauma and PTSD, 2nd ed. New York, NY, US: The Guilford Press; 2004. p. 168-89.

47. Maercker A, Schützwohl M. Erfassung von psychischen Belastungsfolgen: Die Impact of Event Skala-revidierte Version (IES-R). Diagnostica. 1998.

48. Davey C, Heard R, Lennings C. Development of the Arabic versions of the Impact of Events Scale-Revised and the Posttraumatic Growth Inventory to assess trauma and growth in Middle Eastern refugees in Australia. Clinical Psychologist. 2015;19(3):131-9.

49. Sachser C, Berliner L, Holt T, Jensen TK, Jungbluth N, Risch E, et al. International development and psychometric properties of the Child and Adolescent Trauma Screen (CATS). Journal of affective disorders. 2017;210:189-95.

50. Meiser-Stedman R, Smith P, Bryant R, Salmon K, Yule W, Dalgleish T, et al. Development and validation of the child post-traumatic cognitions inventory (CPTCI). Journal of Child Psychology and Psychiatry. 2009;50(4):432-40.

51. de Haan A, Petermann F, Meiser-Stedman R, Goldbeck L. Psychometric properties of the German version of the child post-traumatic cognitions inventory (CPTCI-GER). Child Psychiatry & Human Development. 2016;47(1):151-8.

For peer review only

For peer review only - http://bmjopen.bmj.com/site/about/guidelines.xhtml

52. Meiser-Stedman R. Post-traumatic Cognitions Inventory - Child Version (Arabic version) n. d. [Available from: https://www.childrenandwar.org/wp-content/uploads/2019/06/CPTCI_arabisch.pdf].
53. Krakow B. Nightmare Complaints in Treatment-Seeking Patients in Clinical Sleep Medicine Settings: Diagnostic and Treatment Implications. Sleep. 2006;29:1313-9.
54. Krakow B, Haynes PL, Warner TD, Santana E, Melendrez D, Johnston L, et al. Nightmares, insomnia, and sleep-disordered breathing in fire evacuees seeking treatment for posttraumatic sleep disturbance. Journal of Traumatic Stress: Official Publication of The International Society for Traumatic Stress Studies. 2004;17(3):257-68.
55. Victor SE, Klonsky ED. Validation of a brief version of the difficulties in emotion regulation scale (DERS-18) in five samples. Journal of psychopathology and Behavioral Assessment. 2016;38(4):582-9.
56. Goodman R. Psychometric properties of the strengths and difficulties questionnaire. Journal of the American Academy of Child & Adolescent Psychiatry. 2001;40(11):1337-45.
57. Idzelis M, Ali AS, Gaaddasaar M, Bogoslaw LH, Ulstad K, Tanaka A. n. d. [Available from: https://www.sdqinfo.org/py/sdqinfo/b3.py?language=Somali].
58. Beck AT, Steer RA, Brown GK. Bdi-ii manual. 1996.
59. Selmo P, Koch T, Brand J, Wagner B, Knaevelsrud C. Psychometric properties of the online Arabic versions of BDI-II, HSCL-25, and PDS. European Journal of Psychological Assessment. 2016;35:46-54.
60. Zentrum Überleben. n. d. [Available from: https://www.ueberleben.org/].
61. Refugio Munich. n. d. [Available from: https://www.refugio-muenchen.de/].
62. Pfaltz MC, Michael T, Meyer AH, Wilhelm FH. Reexperiencing symptoms, dissociation, and avoidance behaviors in daily life of patients with PTSD and patients with panic disorder with agoraphobia. Journal of traumatic stress. 2013;26(4):443-50.
63. Price M, Ruggiero KJ, Ferguson PL, Patel SK, Treiber F, Couillard D, et al. A feasibility pilot study on the use of text messages to track PTSD symptoms after a traumatic injury. General Hospital Psychiatry. 2014;36(3):249-54.
64. Ruscio AM, Gentes EL, Jones JD, Hallion LS, Coleman ES, Swendsen J. Rumination predicts heightened responding to stressful life events in major depressive disorder and generalized anxiety disorder. Journal of Abnormal Psychology. 2015;124(1):17-26.
65. movisens GmbH. 2021 [Available from: https://www.movisens.com/de/].
66. Short SJ, Stalder T, Marceau K, Entringer S, Moog NK, Shirtcliff EA, et al. Correspondence between hair cortisol concentrations and 30-day integrated daily salivary and weekly urinary cortisol measures. Psychoneuroendocrinology. 2016;71:12-8.
67. World Medical Association. Declaration of Helsinki: Ethical Principles for Medical Research Involving Human Subjects. JAMA. 2013;310(20):2191-4.
68. General Data Protection Regulation GDPR. n. d. [Available from: https://gdpr-info.eu/].
69. Prasad L, Varrey A, Sisti G. Medical students' stress levels and sense of well being after six weeks of yoga and meditation. Evidence-Based Complementary and Alternative Medicine. 2016;2016.
70. Butollo W, Karl R, König J, Rosner R. A randomized controlled clinical trial of dialogical exposure therapy versus cognitive processing therapy for adult outpatients suffering from PTSD after type I trauma in adulthood. Psychotherapy and Psychosomatics. 2016;85(1):16-26.
71. Raudenbush SW, Bryk AS. Hierarchical linear models: Applications and data analysis methods: Sage Publications; 2002.

For peer review only - http://bmjopen.bmj.com/site/about/guidelines.xhtml

Reporting checklist for protocol of a clinical trial.

SPIRIT 2013 Checklist: Recommended items to address in a clinical trial protocol and related documents

		Reporting Item	Page Number
**Administrative information**			
Title	#1	Descriptive title identifying the study design, population, interventions, and, if applicable, trial acronym	1
Trial registration	#2a	Trial identifier and registry name. If not yet registered, name of intended registry	3
Trial registration: data set	#2b	All items from the World Health Organization Trial Registration Data Set	3
Protocol version	#3	Date and version identifier	6
Funding	#4	Sources and types of financial, material, and other support	21
Roles and responsibilities: contributorship	#5a	Names, affiliations, and roles of protocol contributors	1, 21
Roles and responsibilities:	#5b	Name and contact information for the trial sponsor	21

For peer review only

sponsor contact information			
Roles and responsibilities: sponsor and funder	#5c	Role of study sponsor and funders, if any, in study design; collection, management, analysis, and interpretation of data; writing of the report; and the decision to submit the report for publication, including whether they will have ultimate authority over any of these activities	21
Roles and responsibilities: committees	#5d	Composition, roles, and responsibilities of the coordinating centre, steering committee, endpoint adjudication committee, data management team, and other individuals or groups overseeing the trial, if applicable (see Item 21a for data monitoring committee)	16ff, 21
**Introduction**			
Background and rationale	#6a	Description of research question and justification for undertaking the trial, including summary of relevant studies (published and unpublished) examining benefits and harms for each intervention	4ff
Background and rationale: choice of comparators	#6b	Explanation for choice of comparators	---
Objectives	#7	Specific objectives or hypotheses	5,6

For peer review only

Trial design	#8	Description of trial design including type of trial (eg, parallel group, crossover, factorial, single group), allocation ratio, and framework (eg, superiority, equivalence, non-inferiority, exploratory)	7
**Methods: Participants, interventions, and outcomes**			
Study setting	#9	Description of study settings (eg, community clinic, academic hospital) and list of countries where data will be collected. Reference to where list of study sites can be obtained	7
Eligibility criteria	#10	Inclusion and exclusion criteria for participants. If applicable, eligibility criteria for study centres and individuals who will perform the interventions (eg, surgeons, psychotherapists)	7,8,16
Interventions: description	#11a	Interventions for each group with sufficient detail to allow replication, including how and when they will be administered	11ff., table 2
Interventions: modifications	#11b	Criteria for discontinuing or modifying allocated interventions for a given trial participant (eg, drug dose change in response to harms, participant request, or improving / worsening disease)	12

For peer review only

Interventions: adherance	#11c	Strategies to improve adherence to intervention protocols, and any procedures for monitoring adherence (eg, drug tablet return; laboratory tests)	16
Interventions: concomitant care	#11d	Relevant concomitant care and interventions that are permitted or prohibited during the trial	8
Outcomes	#12	Primary, secondary, and other outcomes, including the specific measurement variable (eg, systolic blood pressure), analysis metric (eg, change from baseline, final value, time to event), method of aggregation (eg, median, proportion), and time point for each outcome. Explanation of the clinical relevance of chosen efficacy and harm outcomes is strongly recommended	5,6, 14-16- 18ff, Figure 1, Table 1
Participant timeline	#13	Time schedule of enrolment, interventions (including any run-ins and washouts), assessments, and visits for participants. A schematic diagram is highly recommended (see Figure)	9-11, table 1
Sample size	#14	Estimated number of participants needed to achieve study objectives and how it was determined, including clinical and statistical assumptions supporting any sample size calculations	18
Recruitment	#15	Strategies for achieving adequate participant enrolment to reach target sample size	9

For peer review only

**Methods: Assignment of interventions (for controlled trials)**			
Allocation: sequence generation	#16a	Method of generating the allocation sequence (eg, computer-generated random numbers), and list of any factors for stratification. To reduce predictability of a random sequence, details of any planned restriction (eg, blocking) should be provided in a separate document that is unavailable to those who enrol participants or assign interventions	14
Allocation concealment mechanism	#16b	Mechanism of implementing the allocation sequence (eg, central telephone; sequentially numbered, opaque, sealed envelopes), describing any steps to conceal the sequence until interventions are assigned	14
Allocation: implementation	#16c	Who will generate the allocation sequence, who will enrol participants, and who will assign participants to interventions	14
Blinding (masking)	#17a	Who will be blinded after assignment to interventions (eg, trial participants, care providers, outcome assessors, data analysts), and how	10
Blinding (masking): emergency unblinding	#17b	If blinded, circumstances under which unblinding is permissible, and procedure for revealing a participant's allocated intervention during the trial	----

For peer review only

Methods: Data collection, management, and analysis

Data collection plan	#18a	Plans for assessment and collection of outcome, baseline, and other trial data, including any related processes to promote data quality (eg, duplicate measurements, training of assessors) and a description of study instruments (eg, questionnaires, laboratory tests) along with their reliability and validity, if known. Reference to where data collection forms can be found, if not in the protocol	10, 13ff, 16ff
Data collection plan: retention	#18b	Plans to promote participant retention and complete follow-up, including list of any outcome data to be collected for participants who discontinue or deviate from intervention protocols	11, 29ff
Data management	#19	Plans for data entry, coding, security, and storage, including any related processes to promote data quality (eg, double data entry; range checks for data values). Reference to where details of data management procedures can be found, if not in the protocol	27
Statistics: outcomes	#20a	Statistical methods for analysing primary and secondary outcomes. Reference to where other details of the	29ff

For peer review only

		statistical analysis plan can be found, if not in the protocol	
Statistics: additional analyses	#20b	Methods for any additional analyses (eg, subgroup and adjusted analyses)	29ff
Statistics: analysis population and missing data	#20c	Definition of analysis population relating to protocol non-adherence (eg, as randomised analysis), and any statistical methods to handle missing data (eg, multiple imputation)	29

Methods: Monitoring

Data monitoring: formal committee	#21a	Composition of data monitoring committee (DMC); summary of its role and reporting structure; statement of whether it is independent from the sponsor and competing interests; and reference to where further details about its charter can be found, if not in the protocol. Alternatively, an explanation of why a DMC is not needed	27
Data monitoring: interim analysis	#21b	Description of any interim analyses and stopping guidelines, including who will have access to these interim results and make the final decision to terminate the trial	29, 31
Harms	#22	Plans for collecting, assessing, reporting, and managing solicited and spontaneously reported adverse events and other unintended effects of trial interventions or trial conduct	15, 31, table 2

For peer review only

Auditing	#23	Frequency and procedures for auditing trial conduct, if any, and whether the process will be independent from investigators and the sponsor	26
**Ethics and dissemination**			
Research ethics approval	#24	Plans for seeking research ethics committee / institutional review board (REC / IRB) approval	27, 34
Protocol amendments	#25	Plans for communicating important protocol modifications (eg, changes to eligibility criteria, outcomes, analyses) to relevant parties (eg, investigators, REC / IRBs, trial participants, trial registries, journals, regulators)	27
Consent or assent	#26a	Who will obtain informed consent or assent from potential trial participants or authorised surrogates, and how (see Item 32)	11, 13
Consent or assent: ancillary studies	#26b	Additional consent provisions for collection and use of participant data and biological specimens in studies, if applicable	----
Confidentiality	#27	How personal information about potential and enrolled participants will be collected, shared, and maintained in order to protect confidentiality before, during, and after the trial	27ff

For peer review only

Declaration of interests	#28	Financial and other competing interests for principal investigators for the overall trial and each study site	35
Data access	#29	Statement of who will have access to the final trial dataset, and disclosure of contractual agreements that limit such access for investigators	34
Ancillary and post trial care	#30	Provisions, if any, for ancillary and post-trial care, and for compensation to those who suffer harm from trial participation	10, 11, 13
Dissemination policy: trial results	#31a	Plans for investigators and sponsor to communicate trial results to participants, healthcare professionals, the public, and other relevant groups (eg, via publication, reporting in results databases, or other data sharing arrangements), including any publication restrictions	27
Dissemination policy: authorship	#31b	Authorship eligibility guidelines and any intended use of professional writers	--
Dissemination policy: reproducible research	#31c	Plans, if any, for granting public access to the full protocol, participant-level dataset, and statistical code	--

Appendices

Informed consent materials	#32	Model consent form and other related documentation given to participants and authorised surrogates	--
Biological specimens	#33	Plans for collection, laboratory evaluation, and storage of biological specimens for genetic or molecular analysis in	--

For peer review only

the current trial and for future use in ancillary studies, if applicable

None The SPIRIT Explanation and Elaboration paper is distributed under the terms of the Creative Commons Attribution License CC-BY-NC. This checklist can be completed online using https://www.goodreports.org/, a tool made by the EQUATOR Network in collaboration with Penelope.ai

For peer review only

Psychometric instruments

If not available psychometric properties for internal consistency and reliability, criterion validity and convergent validity will be calculated within our sample.

Inclusion and exclusion criteria

Essen Trauma Inventory for Children and Adolescents

The Essen Trauma Inventory for Children and Adolescents (ETI-CA; (1, 2)) comprises a list of traumatic events and assesses symptoms of acute adjustment disorder and PTSD according to DSM-IV criteria (3) within the last four weeks, in relation to the most disturbing traumatic event if more than one was experienced. It further assesses the degree of impairment in six areas of daily living (e.g., school, peers) due to the traumatic event. It is available as a self-report questionnaire and an interview version, with the latter being used in this study. ETI-CA has been designed for the age group 12-17 years and is available in German and English. If necessary, we will translate the ETI-CA interview into Arabic, Dari, and Somali.

Clinical Global Impression Scale – Severity of illness

The Clinical Global Impression Scale is an internationally used instrument and yields the three different measures: 1. Severity of illness (CGI-S), 2. Global improvement (CGI-I), and 3. Efficacy index (4). In our study, we will apply the CGI-S, which is a 7-point scale on which the clinician rates the severity of the patient's illness at the time of assessment relative to the clinician's past experience with patients with the same diagnosis. Possible ratings range from 1=not ill at all to 7=among the most extremely ill patients. The German version of the scale (4) is clinically rated by study staff.

Kiddie-Schedule for Affective Disorders and Schizophrenia, Present and Lifetime

For peer review only - http://bmjopen.bmj.com/site/about/guidelines.xhtml

The Schedule for Affective Disorders and Schizophrenia for School-Age Children-Present and Lifetime version (K-SADS-PL) is a widely used, validated, semi-structured diagnostic interview for the standardized assessment of mental disorders according to DSM-5 in children and adolescents aged 6-18 years (5-7). For the purpose of this study we will use the K-SASDS-PL diagnostic screening interview with subsequent diagnostic interviews if indicated, and the summary lifetime diagnosis checklist. The diagnostic screening interview reviews the most severe current and past major symptoms of all diagnoses assessed with the K-SADS-PL. If the participant displays indicative symptoms of a specific disorder according to the screening, the referring diagnostic interviews will be applied. The K-SADS-PL comprises the following diagnostic interviews: depressive and bipolar-related disorders, psychotic disorders, anxiety disorders, obsessive-compulsive disorders, trauma-related disorders including PTSD, externalizing disorders, eating disorders, and substance abuse disorders. The summary lifetime diagnosis checklist then summarizes all mental diagnoses assessed by the K-SADS-PL. The K-SADS-PL is available in German and English for the purpose of the study If needed, the interview is conducted with the assistance of an interpreter.

Alcohol Use Disorders Identification Test

The Alcohol Use Disorders Identification Test (AUDIT) is an internationally used, validated, self-report questionnaire approved by the World Health Organization to screen individuals for hazardous or harmful alcohol consumption and alcohol dependence. It can be applied from age 14 years onwards. Items are rated on a 5-point Likert scale (0-4). Total possible AUDIT scores range from 0-40. Scores of 8-10 or more are indicative of hazardous, harmful alcohol use or possible alcohol dependence. The AUDIT is freely available in many languages, including Arabic,

For peer review only - http://bmjopen.bmj.com/site/about/guidelines.xhtml

English and German (8). For the purpose of our study, we translated the questionnaire into Dari and Somali.

Columbia-Suicide Severity Rating Scale

The Columbia-Suicide Severity Rating Scale (C-SSRS) assesses suicidal ideation and behavior. Questions are phrased for use in an interview format. The C-SSRS measures the following constructs: severity of suicidal ideation, intensity of suicidal ideation, suicidal and/or self-harm behavior, and lethality of suicide attempts. It is provided in a lifetime and recent/since last visit version (9). We use the lifetime version for the first assessment at the screening visit and the recent/since last visit version for all other study visits. In addition to the original validated English version, the C-SSRS is available in a German version and has been translated into Arabic, Dari, and Somali.

Test of Nonverbal Intelligence, fourth edition

The Test of Nonverbal Intelligence, Fourth Edition (TONI 4) is a language-free, validated intelligence test that requires no reading, writing or speaking on the examinee's part. It can be used from the age of six years onwards. Test items present a variety of problem-solving tasks in ascending order of difficulty. The abstract, figural content of the test items reduces the cultural loading of the test and the result is not influenced by insufficient language skills (10).

Questionnaire to Assess Endangerment to Others

The standardized Questionnaire to Assess Endangerment to Others is a self-designed, self-rated questionnaire in accordance with the wording of the C-SSRS (9), which assesses thoughts or behaviors within the last six months displaying endangerment or threat to others. The questionnaire comprises six questions that are answered in a yes/no structure and is provided in Arabic, Dari, English, German and Somali.

For peer review only - http://bmjopen.bmj.com/site/about/guidelines.xhtml

Primary endpoints

Perceived Stress Scale, 10-item version

The Perceived Stress Scale, 10-item version (PSS-10), is a validated self-report questionnaire that assesses psychological distress during the preceding month. It measures the intensity with which individuals appraise their daily life as stressful, unpredictable, uncontrollable, and overloaded. The PSS-10 was designed for use in participants aged 14 years or older. Items are rated on a 6-point scale from 0=never to 5=often. In addition to the original English version (11), a validated German version (12) and an Arabic version are available (13). For the purpose of this study, we translated the PSS-10 into Dari and Somali.

Impact of Event Scale-Revised

The Impact of Event Scale-Revised (IES-R) is a validated, self-report questionnaire assessing traumatic symptoms on three subscales: intrusion (intrusive thoughts, nightmares, intrusive feelings and imagery, dissociative-like re-experiencing), avoidance (numbing of responsiveness, avoidance of feelings, situations and ideas), hyperarousal (anger, irritability, hypervigilance, difficulty concentrating, heightened startle), and an overall measure of traumatic distress. Items are rated on a 5-point Likert scale from 0=not at all to 4=extreme. In addition to the original English version (14, 15), a validated German version (16) and an Arabic version (17) are available. For the purpose of this study, we translated the IES-R into Dari and Somali.

Secondary endpoints

Trauma symptoms

Child and Adolescent Trauma Screen-2

The Child and Adolescent Trauma Screen-2 (CATS-2) is a validated, freely accessible self-report questionnaire based on the DSM-5 PTSD criteria. It assesses DSM-5

For peer review only - http://bmjopen.bmj.com/site/about/guidelines.xhtml

criteria A (experience of a traumatic event ever), B (intrusion, re-experiencing), C (avoidance), D (negative alterations in cognitions and mood), and E (hyperarousal). Criteria B-D are rated on a 4-point scale (0=never, 3=almost always). Trauma-related psychosocial impairment (peers, family, school/work, hobbies, well-being) is assessed with yes/no items. For the purpose of this study, we use the validated English and German CATS-2 self-report versions (18) and translated the scale into Arabic, Dari and Somali.

Posttraumatic Cognitions Inventory - child version

The Posttraumatic Cognitions Inventory - child version (cPTCI) is a validated, self-report questionnaire assessing trauma-related cognitions on the subscales 'permanent and disturbing change', 'fragile person in a scary world' and on an overall score. Items are rated on a 5-point Likert-type scale and are summed to form a total score and the two subscales scores. For the purpose of this study, we use the validated English version (19), a validated German (20) and an Arabic cPTCI version (21) and translated the cPTCI into Dari and Somali.

Disturbing Dream and Nightmare Severity Index

The Disturbing Dream and Nightmare Severity Index (DDNSI) is a validated, self-report questionnaire and assesses number of nights with nightmares per week (0-7 nights), total number of nightmares per week, severity of nightmares, intensity of nightmares (Likert-type scale: 0=no problem to 6=extremely severe problem), how often nightmares result in awakenings (0=never/rarely to 4=always). The scale provides five sub-scores and a total score (22, 23). For the purpose of this study, the DDNSI was translated into Arabic, Dari, German, and Somali.

Emotion Regulation

Difficulties in Emotion Regulation Scale

For peer review only - http://bmjopen.bmj.com/site/about/guidelines.xhtml

The 18-item version of the Difficulties in Emotion Regulation Scale (DERS-18; (24)) is a validated, self-report questionnaire assessing the following six components of emotion dysregulation on a 5-point Likert-type scale: 1) Nonacceptance of emotional responses, 2) Difficulties engaging in goal-directed behavior, 3) Impulse control difficulties, 4) Lack of emotional awareness, 5) Limited access to emotion regulation strategies, 6) Lack of emotional clarity. It can be used from the age of 13 years onwards and yields a total score as well as subscale scores. For the purpose of the study, we use the 18 items from the unpublished German validation study of the DERS-36 (25, 26) and translated the English version of the DERS-18 (24) into Arabic, Dari and Somali.

General Psychopathology

Strengths and Difficulties Questionnaire

The Strengths and Difficulties Questionnaire (SDQ) is a widely used, validated parent-/caregiver-rated and/or self-report screening questionnaire for children and adolescents that is freely available online in an English version (27), German version (28), Arabic version (29), and Somali parent/caregiver version (30). The SDQ screens for difficulties in four areas: 1) emotional symptoms, 2) conduct problems, 3) hyperactivity/inattention, and 4) peer relationship problems. It additionally assesses strengths in prosocial behavior and provides a total score that sums up all subscale scores. For the purpose of this study, we use the available Arabic, English, German, self-report, and caregiver SDQ as well as the Somali caregiver version, and translated the SDQ caregiver and self-report version into Dari and the self-report version into Somali.

Beck Depression Inventory, second edition

The Beck Depression Inventory, second edition (BDI-II) is a widely used, validated, self-report questionnaire that assesses the severity of depression according to DSM-

For peer review only - http://bmjopen.bmj.com/site/about/guidelines.xhtml

IV criteria on a 4-point Likert-like scale. It can be used in adolescents from age 13 years onwards. The BDI-II is available in a validated English (31), validated German (32) and a validated Arabic version (33), which we use in this study as well as a translated Dari and Somali version

References

1. Graham FS, W, Tagay S. ETI-CA Essen Trauma-Inventory for Children and Adolescents - Interview Essen: LVR-Klinikum Essen, Universität Duisburg; 2012.
2. Tagay S, Düllmann S, Hermans E, Repic N, Hiller R, Senf W. Das Essener Trauma-Inventar für Kinder und Jugendliche (ETI-KJ). Zeitschrift für Kinder-und Jugendpsychiatrie und Psychotherapie. 2011;39:323-40.
3. American Psychiatric Association. Diagnostic and Statistical Manual of Mental Disorders (DSM-IV) Fourth Edition. Washington, DC: American Psychiatric Association; 1994.
4. Guy W. Clinical global impression. Assessment manual for Psychopharmacology. 1976:217-22.
5. Kaufman J, Birmaher B, Brent D, Rao U, Flynn C, Moreci P, et al. Schedule for affective disorders and schizophrenia for school-age children-present and lifetime version (K-SADS-PL): initial reliability and validity data. Journal of the American Academy of Child & Adolescent Psychiatry. 1997;36(7):980-8.
6. American Psychiatric Association. Diagnostic and Statistical Manual of Mental Disorders. 5th ed. Washington, DC2013.
7. Kaufman J, Birmaher B, Axelson D, Perepletchikova F, Brent D, Ryan N. Schedule for affective and disorders and schizophrenia for school aged children (6–18 years): Kiddie-SADS-lifetime version (K-SADS-PL DSM 5). Pittsburgh, PA: Western Psychiatric Institute and Clinic; 2016.
8. National Institute on Drug Abuse. Audit. 2013.
9. Posner K, Brown GK, Stanley B, Brent DA, Yershova KV, Oquendo MA, et al. The Columbia–Suicide Severity Rating Scale: initial validity and internal consistency findings from three multisite studies with adolescents and adults. American journal of psychiatry. 2011;168(12):1266-77.
10. Brown L, Sherbenou RJ, Johnsen SK. Test of nonverbal intelligence: TONI-4: Pro-ed Austin, TX; 2010.
11. Cohen S, Williamson G. Perceived stress in a probability sample of the United States. In: Spacapan S, & Oskamp, S., editor. The Social Psychology of Health: Claremont Symposium on Applied Social Psychology Newbury Park, CA: Sage; 1988. p. 31-67.
12. Klein EM, Brähler E, Dreier M, Reinecke L, Müller KW, Schmutzer G, et al. The German version of the Perceived Stress Scale–psychometric characteristics in a representative German community sample. BMC psychiatry. 2016;16(1):1-10.
13. Chaaya M, Osman H, Naassan G, Mahfoud Z. Validation of the Arabic version of the Cohen Perceived Stress Scale (PSS-10) among pregnant and postpartum women. BMC psychiatry. 2010;10(1):111.
14. Weiss DS, Marmar CR. The Impact of Event Scale—Revised. Assessing psychological trauma and PTSD. New York, NY, US: The Guilford Press; 1997. p. 399-411.
15. Weiss DS. The Impact of Event Scale-Revised. Assessing psychological trauma and PTSD, 2nd ed. New York, NY, US: The Guilford Press; 2004. p. 168-89.
16. Maercker A, Schützwohl M. Erfassung von psychischen Belastungsfolgen: Die Impact of Event Skala-revidierte Version (IES-R). Diagnostica. 1998.
17. Davey C, Heard R, Lennings C. Development of the A rabic versions of the I mpact of E vents S cale-R evised and the P osttraumatic G rowth I nventory to assess trauma and growth in M iddle E astern refugees in A ustralia. Clinical Psychologist. 2015;19(3):131-9.

For peer review only - http://bmjopen.bmj.com/site/about/guidelines.xhtml

18. Sachser C, Berliner L, Holt T, Jensen TK, Jungbluth N, Risch E, et al. International development and psychometric properties of the Child and Adolescent Trauma Screen (CATS). Journal of affective disorders. 2017;210:189-95.
19. Meiser-Stedman R, Smith P, Bryant R, Salmon K, Yule W, Dalgleish T, et al. Development and validation of the child post-traumatic cognitions inventory (CPTCI). Journal of Child Psychology and Psychiatry. 2009;50(4):432-40.
20. de Haan A, Petermann F, Meiser-Stedman R, Goldbeck L. Psychometric properties of the German version of the child post-traumatic cognitions inventory (CPTCI-GER). Child Psychiatry & Human Development. 2016;47(1):151-8.
21. Meiser-Stedman R. Post-traumatic Cognitions Inventory - Child Version (Arabic version) n. d. [Available from: https://www.childrenandwar.org/wp-content/uploads/2019/06/CPTCI_arabisch.pdf.
22. Krakow B. Nightmare Complaints in Treatment-Seeking Patients in Clinical Sleep Medicine Settings: Diagnostic and Treatment Implications. Sleep. 2006;29:1313-9.
23. Krakow B, Haynes PL, Warner TD, Santana E, Melendrez D, Johnston L, et al. Nightmares, insomnia, and sleep-disordered breathing in fire evacuees seeking treatment for posttraumatic sleep disturbance. Journal of Traumatic Stress: Official Publication of The International Society for Traumatic Stress Studies. 2004;17(3):257-68.
24. Victor SE, Klonsky ED. Validation of a brief version of the difficulties in emotion regulation scale (DERS-18) in five samples. Journal of psychopathology and Behavioral Assessment. 2016;38(4):582-9.
25. Ehring T, Svaldi J, Tuschen-Caffier B, Berking M. Validierung der Difficulties in Emotion Regulation Scale–deutsche Version (DERS-D). Unveröffentlichtes Manuskript, Universität Münster. 2013.
26. Gutzweiler R, In-Albon T. Überprüfung der Gütekriterien der deutschen Version der Difficulties in Emotion Regulation Scale in einer klinischen und einer Schülerstichprobe Jugendlicher. Zeitschrift für Klinische Psychologie und Psychotherapie. 2019;47:274-86.
27. Goodman R. Psychometric properties of the strengths and difficulties questionnaire. Journal of the American Academy of Child & Adolescent Psychiatry. 2001;40(11):1337-45.
28. Klasen H, Woerner W, Rothenberger A, Goodman R. Die deutsche Fassung des strengths and difficulties questionnaire (SDQ-Deu)-Übersicht und Bewertung erster Validierungs-und Normierungsbefunde. 2003.
29. Emam MM, Hilal MMA, Kazem AM, Alkharousi SJ. Psychometric properties of the Arabic self-report version of the strengths and difficulties questionnaire. Research in developmental disabilities. 2016;59:211-20.
30. Idzelis M, Ali AS, Gaaddasaar M, Bogoslaw LH, Ulstad K, Tanaka A. n. d. [Available from: https://www.sdqinfo.org/py/sdqinfo/b3.py?language=Somali.
31. Beck AT, Steer RA, Brown GK. Bdi-ii manual. 1996.
32. Hautzinger M, Keller F, Kühner C. Beck depressions-inventar (BDI-II): Harcourt Test Services; 2006.
33. Selmo P, Koch T, Brand J, Wagner B, Knaevelsrud C. Psychometric properties of the online Arabic versions of BDI-II, HSCL-25, and PDS. European Journal of Psychological Assessment. 2016;35:46-54.

For peer review only

BMJ Open

START Adolescents: Study protocol of a randomized controlled trial to investigate the efficacy of a low-threshold group treatment program in traumatized adolescent refugees

Journal:	*BMJ Open*
Manuscript ID	bmjopen-2021-057968.R1
Article Type:	Protocol
Date Submitted by the Author:	24-Nov-2021
Complete List of Authors:	Sobanski, Esther; University Medical Centre of the Johannes Gutenberg University Mainz, Department of Child and Adolescent Psychiatry and Psychotherapy; Central Institute of Mental Health, Psychiatry and Psychotherapy<br>Hammerle, Florian; University Medical Centre of the Johannes Gutenberg University Mainz, Department of Child and Adolescent Psychiatry and Psychotherapy<br>Dixius, Andrea; SHG Saarland Hospital Group, Department of Child and Adolescent Psychiatry<br>Möhler, Eva; SHG Saarland Hospital Group, Child and Adolescent Psychiatry<br>Koudela-Hamila, Susanne ; Karlsruhe Institute of Technology, Department of Applied Psychology, Mental mHealth Lab<br>Ebner-Priemer, Ulrich; Karlsruhe Institute of Technology, Department of Applied Psychology, Mental mHealth Lab; Central Institute of Mental Health<br>Merz, Christian; Ruhr University Bochum, Department of Cognitive Psychology, Institute of Cognitive Neuroscience<br>In-albon, Tina; University of Koblenz Landau - Campus Landau, Department of Child and Adolescent Psychology and Psychotherapy<br>Pollitt, Brigitte; Johanniter Clinics, Child and Adolescent Psychiatry and Psychotherapy<br>Christiansen, Hanna; University of Marburg, Department of Psychology, Clinical Child and Adolescent Psychology<br>Kolar, David; University Medical Centre of the Johannes Gutenberg University Mainz, Department of Child and Adolescent Psychiatry<br>Ocker, Susanne; University Medical Centre of the Johannes Gutenberg University Mainz, Child and Adolescent Psychiatry and Pschotherapy<br>Fischer, Nicole; University Medical Centre of the Johannes Gutenberg University Mainz, Child and Adolescent Psychiatry and Pschotherapy<br>Burghaus, Ina; University of Heidelberg, Coordination Centre for Clinical Trials<br>Huss, Michael; University Medical Centre of the Johannes Gutenberg University Mainz, Department of Child and Adolescent Psychiatry
**Primary Subject Heading**:	Mental health

Secondary Subject Heading:	Mental health
Keywords:	Child & adolescent psychiatry < PSYCHIATRY, Protocols & guidelines < HEALTH SERVICES ADMINISTRATION & MANAGEMENT, PSYCHIATRY

SCHOLARONE™ Manuscripts

START Adolescents: Study protocol of a randomized controlled trial to investigate the efficacy of a low-threshold group treatment program in traumatized adolescent refugees

*Esther Sobanski[1,2] (0000-0001-7824-3959), *Florian Hammerle[1](0000-0002-2659-6266), Andrea Dixius[3], Eva Möhler[4], Susanne Koudela-Hamila[5], Ulrich Ebner-Priemer[2,5], Christian J. Merz[6], Tina In-Albon[7], Brigitte Pollitt[8], Hanna Christiansen[9], David R. Kolar[1], Susanne Ocker[1], Nicole Fischer[1], Ina Burghaus[10], Michael Huss[1]

***The first two authors contributed equally to the article**

[1]Department of Child and Adolescent Psychiatry and Psychotherapy, University Medical Center, Johannes Gutenberg University Mainz, Mainz, Germany; E-Mail: esther.sobanski@unimedizin-mainz.de; florian.hammerle@unimedizin-mainz.de; david.kolar@med.uni-muenchen.de; susanne.ocker@unimedizin-mainz.de; nicole.fischer@unimedizin-mainz.de; michael.huss@unimedizin-mainz.de;

[2]Department of Psychiatry and Psychotherapy, Central Institute of Mental Health, Medical Faculty Mannheim/Heidelberg University, Mannheim, Germany;

[3]Department of Child and Adolescent Psychiatry, SHG Clinics, 55743 Idar-Oberstein, Germany; E-Mail: a.dixius@io.shg-kliniken.de; [4]Department of Child and Adolescent Psychiatry, SHG Clinics, 66271 Kleinblittersdorf, Germany; E-Mail: e.moehler@sb.shg-kliniken.de; [5]Department of Applied Psychology, Mental mHealth Lab, Karlsruhe Institute of Technology, 76049 Karlsruhe, Germany; E-Mail: susanne.koudela@kit.edu; ulrich.ebner-priemer@kit.edu; [6]Department of Cognitive Psychology, Institute of Cognitive Neuroscience, Faculty of Psychology, Ruhr University Bochum, 44801 Bochum, Germany; E-Mail: Christian.J.Merz@ruhr-uni-bochum.de; [7]Department of Child and Adolescent Psychology and Psychotherapy, University of Koblenz-Landau, Campus Landau, 76829 Landau, Germany; E-Mail: in-albon@uni-landau.de; [8]Department of Child and Adolescent Psychiatry, Johanniter Clinics Neuwied, 56564 Neuwied, Germany; E-Mail: brigitte.pollitt@nr.johanniter-kliniken.de; [9]Department of Psychology, Clinical Child and Adolescent Psychology, University of Marburg, 35032 Marburg, Germany; E-Mail: hanna.christiansen@staff.uni-marburg.de; [10]Coordination Centre for Clinical Trials, 69120 Heidelberg, Germany; E-Mail: ina.burghaus@med.uni-heidelberg.de;

Corresponding author: Esther Sobanski; E-mail: esther.sobanski@unimedizin-mainz.de; Address: Department of Child and Adolescent Psychiatry and Psychotherapy, University Medical Center, Johannes Gutenberg University Mainz, Langenbeckstr. 1, 55131 Mainz, Germany; Phone: +49 6131 17-3285; fax: +49 6131 17-5580

For peer review only

Abstract

Introduction: No evaluated therapeutic approaches, that can efficiently be established in routine mental health care, are currently available for traumatized adolescent refugees in Germany. This study evaluates the efficacy of the **S**tress-**T**raumasymptoms-**A**rousal-**R**egulation-**T**reatment (**START**) program to reduce trauma-related symptoms and psychological distress in traumatized adolescent refugees based in Germany.

Methods and analysis: This randomized, waiting-list-controlled, multi-center trial with a 12-week follow-up will include 174 refugee minors with partial or full posttraumatic stress disorder who are fluent in either Arabic, Dari, English, German or Somali. Eligible refugee minors will be randomized to the START or waiting-list control groups. The manualized 8-weeks START program is based on techniques of dialectical behavior therapy, fosters adaptive coping with emotional distress and traumatic symptoms and comprises eight therapy modules and a booster session. Study assessments are planned at baseline, post-treatment (i. e., after program participation or waiting time), booster session at week 12 or 12-week waiting time, and at the 12-week follow-up. Primary and co-primary outcomes are changes in psychological distress and traumatic symptoms at post-treatment and will be analyzed as response variables in linear mixed regression models. Secondary outcomes are changes in further trauma-related and other psychopathological symptoms, emotion regulation, and intermediate effects of the program at follow-up. We will also assess effects of the program with ecological momentary assessments and on neuroendocrine stress parameters using hair cortisol.

Ethics and dissemination: This study has been approved by the lead ethics committee of Rhineland-Palatinate and the ethics committees of participating sites.

For peer review only

For peer review only - http://bmjopen.bmj.com/site/about/guidelines.xhtml

The study results will be disseminated through peer-reviewed publications and scientific conferences.

Registration details: German Clinical Trials Register (reference number: DRKS00020771), 21 April 2020

Strengths and limitations of this study

- Randomized waiting-list controlled multicenter trial with follow-up.
- Manualized DBT-based psychotherapeutic program with patient material available in Arabic, Dari, English, German, and Somali.
- Multimodal assessment approach of intervention effects on symptoms, functioning and daily living outcomes by psychometric evaluations, electronic ambulatory assessments (e-diaries), and neuroendocrine stress parameters.
- Restriction of study participation to refugee minors with language skills in Arabic, Dari, English, German, and Somali.

For peer review only

Background and rationale

Among the forcibly displaced persons worldwide there is a high number of children and adolescents under the age of 18 years (1). At the peak of the refugee influx in Germany in 2016, 261.386 of the 745.545 asylum applications recorded in total were filed from refugees under the age of 18 of which 49.786 applications were filed from unaccompanied refugee minors in the care of youth welfare services and without the company of parents or persons with the right of custody. The high percentage of asylum applications filed by refugee minors has remained stable since then though due to political changes the total number of refugees entering Germany has steadily decreased (2, 3). Of all refugees, children and adolescents, and especially unaccompanied refugee minors, are the most vulnerable group. They are at particular risk of traumatization before, during, and after their flight as well as for mental health problems (4-6). Children and adolescents seeking asylum in Germany report an average of eight potentially traumatic experiences, with 98% of unaccompanied refugee minors reporting traumatic events (5-7). They are exposed to stress due to insecure legal status and adaptation to new environments, cultural demands, and language, the loss of stability provided by their cultural background of origin as well as provided by their families and caregivers if unaccompanied (8). In addition to their often prevalent traumatization, they are also confronted with age-related developmental tasks like peer group integration, identity formation, education, professional training, and finding their role in the host society. Cross-sectional studies and reviews assessing mental health problems in refugee minors report prevalence rates of 17-71% for posttraumatic stress disorder (PTSD), 12-44% for depressive disorders, 18-38% for anxiety disorders, and 33-72% for behavior problems (9, 10). Longitudinal studies suggest a high risk of chronic mental health problems and indicate that baseline symptom severity, sex, being unaccompanied and post-migration factors like asylum

For peer review only - http://bmjopen.bmj.com/site/about/guidelines.xhtml

status and access to mental health care are predictors of future mental health status (6, 11). Studies evaluating trauma-related therapy programs in refugee minors have mostly reported significant effects on trauma and/or additional mental health symptoms. However, conclusive interpretation of the findings is limited by heterogeneous therapy approaches, lack of manualized programs and methodological shortcomings like lack of power analyses, randomization and control groups, small sample sizes or heterogeneous study settings such as clinical, school or community settings in high-income countries, but also refugee camps in war regions (12-17). Evidence from rigorous methodological approaches like randomized controlled trials (RCT) is needed to cross-validate the available results in terms of validity, reliability, and generalizability of the programs.

Study objectives

The manualized Stress-Traumasymptoms-Arousal-Regulation-Treatment (START) program was developed from clinical work with traumatized unaccompanied refugee minors in Germany to promote coping with traumatic distress and emotional irritability (18-20). An uncontrolled pilot study in 22 traumatized refugee minors showed positive effects on emotion regulation, adaptive strategies, self-control, distress, and good feasibility (18). A subsequent study confirmed the positive effects on adaptive emotion regulation strategies (21).

The primary aim of this RCT is to evaluate the efficacy of the START program in reducing psychological distress and trauma symptoms in traumatized adolescent refugees compared to a waiting-list control group (WL). Main secondary aims are to assess whether treatment effects will remain stable for at least 12 weeks after program termination (follow-up at week 24) and to assess whether the participants of the START groups improve emotion regulation strategies and mental health compared to WL. As

For peer review only - http://bmjopen.bmj.com/site/about/guidelines.xhtml

stress and emotions are context-dependent and highly dynamic (22), intervention effects are assessed by psychometric instruments and with electronic ambulatory assessment (e-diaries). Ambulatory assessment aims to reduce retrospective biases while gathering ecologically valid data from everyday life near real-time. It has been shown as superior to retrospective psychometric assessment in terms of predicting symptom and treatment outcome (23-26) and assessing reliably everyday functioning (27). We also evaluate intervention effects on the neuroendocrine stress system by assessing hair cortisol, which reflects the cumulative cortisol release over the past months and has been shown to capture treatment effects in PTSD (28).

The study is part of the START research consortium, which evaluates distress-reducing psychotherapeutic group and preventive family interventions in traumatized toddler, adolescent, and young adult refugees and aims to contribute to improved evidence-based therapeutic interventions for refugees within a stepped mental health care approach. The consortium comprises the START Adolescent Study, the START Childcare Study (29), the START Young Adults Study, and the smartphone-based experience sampling study tracking symptoms and daily living functioning with the use of e-diaries across the START Adolescents and Childcare studies. For further information regarding the consortium, please see www.mentalhealth4refugees.de. The presented paper reports on the START Adolescents protocol (Version 4.0, March 16th, 2020) and has been conceived according to the SPIRIT guidelines (30, 31). Please also refer to SPIRIT checklist, supplementary file 1, and to table 1.

Methods and analysis

Patient and public involvement

The original START program was developed from clinical work with highly traumatized unaccompanied refugee minors of different countries of origin, who had recently

For peer review only - http://bmjopen.bmj.com/site/about/guidelines.xhtml

arrived in Germany (18-20). The adapted and extended version of the program, that is evaluated in this RCT, was redesigned according to the participants' feedback of the pilot study groups, who were also traumatized refugee minors from different countries of origin. The public was not involved when designing the study.

Trial design and setting

The study is conducted as a 12-weeks, two-arm, randomized, waiting-list-controlled, multi-center trial with a 12-week follow-up and six participating outpatient departments in Germany, all of which are experienced in treating traumatized adolescent refugees. The study is coordinated by the Department of Child and Adolescent Psychiatry and Psychotherapy, University Medical Center, Johannes Gutenberg University, Mainz, Germany. The study intervention comprises an adapted version of the START program (18-20). Languages used in the study for the intervention and the assessments are Arabic, Dari, English, German or Somali. Interpreters are involved if needed. The study is not blinded for the treatment condition, which will be evident to participants, caregivers, and study therapists. Recruitment started 1 October 2020. We expect that the last participants will complete the study end of October 2022. Data base lock and analysis of primary outcomes are planned until end of December 2022.

Key stopping rules for patients are a withdrawal of informed consent, unwillingness to further participate in the trial, any factors affecting the patient's or others' well-being, e.g. acute suicidality or acute endangerment to others, onset of other acute severe mental disorders, alcohol or substance abuse, inpatient treatment of over two days, start of concurrent psychotherapy and more than two psychotherapeutic crisis interventions. Key stopping rules for participating centers are non-adherence with the ICH-GCP (32) or the study protocol, insufficient recruitment of participants, or

For peer review only - http://bmjopen.bmj.com/site/about/guidelines.xhtml

insufficient data quality. The key stopping rule of the trial is a change in the overall risk-benefit ratio.

Participants

We will allocate 174 participants to the trial and expect that we will have to screen 240 adolescent refugees for eligibility. Inclusion criteria are: informed consent by adolescents and caregivers, flight background, age between 13;00 and 17;11 years, verbal communication skills and reading comprehension in one of the study languages, non-verbal IQ ≥ 70, partial or full PTSD, PTSD symptoms are the clinically most impairing condition with at least moderate clinical symptom severity. We kept the exclusion criteria to a minimum, to foster the applicability of the program in routine care as much as possible. Exclusion criteria are**:** current substance use disorder and/or harmful alcohol abuse, primary severe mental disorders other than PTSD, acute suicidality or danger to others, unstable psychotropic medication (change of psychotropic medication within the last two weeks before baseline assessment), current ongoing psychotherapy, inpatient status or study participation in another clinical trial, unaccompanied refugee minor without a legal representative, known pregnancy. Participants, who attend all assessment visits, will be given a study compensation. Participants will be insured by a clinical trial insurance during the time of study participation. Please see for detailed information about the assessment of eligibility criteria also the sections “screening” and “psychometric instruments”. The participants’ study flow is provided in figure 1.

Insert „*Figure 1: Participants‘ study flow*“ here please

Study conduct

For peer review only - http://bmjopen.bmj.com/site/about/guidelines.xhtml

Recruitment

For recruitment, study information is provided to schools, child and adolescent psychotherapists/psychiatrists, social workers, youth welfare providers, at conferences, professional working group meetings, in newspaper articles or interviews, on the institutions' homepages, and on social media. All participants' information about the study is provided in a caregiver and adolescents' version in all study languages.

Trial flow

For a summary of trial flow and assessment visits please see the SPIRIT flow diagram, table 1. All assessments are provided by trained study staff and are performed at the screening visit, baseline (T0), post-treatment (after 8-week START program or 8-week waiting time; T1), after the booster session or 12-week waiting time (T2), and after 24 weeks at the 12-week follow-up (T3). The maximum time allowed between T0 and T3 is 26 weeks. After termination of the study, all participants are informed about their current diagnostic status and available interventions provided by child and adolescent psychiatric and psychotherapeutic routine care. Participants of the WL are given the opportunity to take part in independent START groups.

Screening visit: Assessment of informed consent for study participation, inclusion and exclusion criteria, demographic and medical data. Inclusion of eligible participants, randomization to the intervention (START) or WL using a web-based randomization system with permuted blocks stratified by center (http://randomizer.at).

Baseline (T0), post-treatment (T1), booster session (T2), 12-week follow-up (T3) assessments: T0 takes place between one and two weeks after randomization (WL) or before the first program session (START), T1 within one week after the 8-week program (START) or within 8-9 weeks after T0 (WL), T2 within one week after the booster session (START) or 4-5 weeks after T1 (WL) and T3 follow-up visits within 12 weeks after T2 (START/WL). At T0 only, the Post-Migration Living Difficulties

For peer review only - http://bmjopen.bmj.com/site/about/guidelines.xhtml

Questionnaire (PMLD) and Questionnaire on Therapy Expectation are assessed. At the T0(T1)-T3 visits primary endpoints, trauma symptoms, emotion regulation, general psychopathology, overall clinical severity of mental disorders, suicidality and endangerment to others are assessed and one week e-diaries are applied. Hair cortisol is assessed at T0, T2 and T3. Adverse events are assessed at T0-T3 visits and weekly until T1.

	STUDY PERIOD				
**TIME POINT**	**Screening**	**T0**<br>**Study visit**<br>**Baseline**<br>One week after random. (WL) or before START Program	**T1**<br>**Study visit**<br>8 weeks after T0 (WL) or after START Program	**T2**<br>**Study visit**<br>4 weeks after T1 (WL) or at booster session (START)	**T3**<br>**Study visit**<br>**Follow-up**<br>12 weeks after T2
**ENROLMENT**					
Eligibility screen	X				
Informed consent	X				
Randomization	X				
**ASSESSMENTS**					
Demographic data	X				
Medical history	X				
Medication/ psychotherapy	X	X	X	X	X
Saliva drug screening	X				
TONI-4	X				
ETI-CA	X		X	X	X
K-SADS-PL	X				
CGI-S	X		X	X	X
C-SSRS	X		X	X	X
REB[1]		**Weekly**			
AUDIT	X				
Endangerm. to others	X		X	X	X
PSS-10	X	X	X	X	X
IES-R	X	X	X	X	X
SDQ self		X	X	X	X
SDQ caregiver	X		X	X	X
BDI-II		X	X	X	X
DERS18		X	X	X	X
cPTCI		X	X	X	X
CATS-2		X	X	X	X
DDNSI		X	X	X	X
PMLD		X			
Therapy expectation		X			

For peer review only - http://bmjopen.bmj.com/site/about/guidelines.xhtml

E-Diaries		X	X	X	X
Hair cortisol		X		X	X
AE assessments		X	X	X	X
**INTERVENTION**					
START program		➡	➡		
Booster session				◆	

Table 1: SPIRIT Flow Diagram

[1]First assessment at T0 then weekly until T3; AE: Adverse events; WL: Waiting-list group. Instruments: Alcohol Use Disorders Identification Test (AUDIT); Beck Depression Inventory, second edition (BDI-II); Child and Adolescent Trauma Screen-2 (CATS-2); Clinical Global Impression Scale, severity of illness subscale (CGI-S); Columbia-Suicide Severity Rating Scale (C-SSRS); Disturbing Dream and Nightmare Severity Index (DDNSI); Difficulties in Emotion Regulation Scale, 18-item version (DERS-18); Essen Trauma Inventory for Children and Adolescents (ETI-CA); Impact of Event Scale-Revised (IES-R); Perceived Stress Scale, 10-item version (PSS-10); Posttraumatic Cognitions Inventory - child version (cPTCI); Schedule for Affective Disorders and Schizophrenia for School-Age Children-Present and Lifetime version (K-SADS-PL); Strengths and Difficulties Questionnaire (SDQ); Test of Nonverbal Intelligence, Fourth Edition (TONI 4)

Intervention

Details of the START Adolescents program are provided in table 2. START Adolescents study groups comprise three to eight participants and are restricted to study participants, who have been randomized to the intervention group. The manualized START group program is based on techniques of dialectical behavioral therapy with emphasis on coping with emotional distress and traumatic symptoms, resilience, adaptive functioning, and self-efficacy, but not on confrontation with traumatic memories. The group therapy is provided by two therapists through twice-weekly 60-minute sessions and interactions are based on validation and acceptance techniques. Written participant program materials are available in all study languages and if necessary, translators will attend the group therapies.

The original START Adolescents program comprised five modules with psychoeducation on trauma, stress, distressing emotions and crises, mindfulness training, arousal regulation, stress reduction techniques, and handling nightmares. For the current study, the START manual (18-20) was extended by the three additional

For peer review only

modules 6-8 and a booster session. The additional modules provide an intensified training of stress reduction skills, mindfulness techniques, and additional psychoeducation about emotions and interpersonal effectiveness. Each session follows a defined structure and comprises, as fixed repeated elements, mindfulness training, monitoring one's own inner tension/stress ("stress signal light"), reviewing experiences when applying the program skills, new contents/psychoeducation, and skills training. The final session in module 8 is dedicated to a farewell ceremony and to validating and rewarding the participants' efforts and achievements. During the booster session, which is scheduled four weeks after program termination, participants review their experiences when applying the acquired skills and techniques, their open needs, and provide an outlook on their personal future wishes and goals. If standardized risk assessments or participants' personal information reveal acute danger to the self or others, additional psychotherapeutic crisis interventions will be offered to the participant. Participants with ongoing acute suicidal tendencies or ongoing danger to others after two subsequent crisis interventions must be excluded from study participation and transferred to specific intense psychiatric care.

**Before beginning of START**: Written patient information. Handout.<br>**Every session**: Mindfulness practice and stress signal light. Discussing between-session skills practice. Written patient material.<br>**Between the sessions:** Participants are asked to train skills and techniques.		
	**Key interventions**	**Practice / Training techniques**
**Module 1**<br>Week 1<br>Sessions 1 & 2	***Welcome.*** Introduction to START.<br>Introduction of participants.<br>***Psychoeducation.*** Explanation of the concepts stress, high stress and stress-regulation skills.<br>***Teaching techniques.*** Skills for reducing distress and high distress. Relaxation techniques.	High-stress skills.<br>Progressive muscle relaxation.
**Module 2**<br>Week 2<br>Sessions 3 & 4	***Consolidation:*** Available skills.<br>***Teaching techniques.*** Stress signal light for stress / inner tension monitoring and awareness. Emotion regulation strategies: mindful senses (hearing, feeling, tasting, watching, smelling). Skills box.	Mindful sensual experiences.<br>Individual stress signal lights.<br>Individual stimuli for distress/ euthymia.<br>High-stress skills.<br>Individual skills boxes.
**Module 3**	***Consolidation.*** Individual skills boxes.	Skills chains

For peer review only - http://bmjopen.bmj.com/site/about/guidelines.xhtml

Week 3<br>Sessions 5 & 6	***Teaching techniques.*** Skills chains. Individual red light. Self-empowerment / resilience.	Self-empowerment
**Module 4**<br>Week 4<br>Sessions 7 & 8	***Consolidation.*** Available skills.<br>***Psychoeducation.*** Explanation of the concept of crisis and management of crisis.<br>***Teaching techniques.*** Managing crisis: acceptance / skills / safe place. Managing flashbacks. Safety and emergency card. How to recognize change. How to change the moment?	Recognition and managing of crisis.<br>Individual safe place.<br>Skills that change the moment.
**Module 5**<br>**Week 5**<br>Sessions 9 & 10	***Consolidation.*** Available skills.<br>***Psychoeducation.*** Sleep disturbances and nightmares.<br>***Teaching techniques.*** Managing nightmares.	Telling / writing nightmares.<br>Skills for managing nightmares.
**Module 6**<br>Week 6<br>Sessions 11 & 12	***Consolidation.*** Available skills.<br>***Psychoeducation.*** Emotions and emotion awareness.<br>***Teaching techniques***. Emotion surfing "Letting go of emotional suffering"	Recognizing and naming emotions (emotion cards).<br>Individual pleasant and unpleasant emotions.<br>Emotion surfing.
**Module 7**<br>Week 7<br>Sessions 13 & 14	***Consolidation.*** Available skills, emotion recognition and understanding.<br>***Psychoeducation.*** Emotional network: Interdependence of emotions, thinking, behavior and prompting stimuli. Skills for emotion regulation.	Role-play/ competition: Recognizing emotions and thoughts.<br>Understand, name and regulate emotions and related behavior and thoughts.
**Module 8**<br>Week 8<br>Sessions 15 & 16	**Review and consolidation:** Available skills, current difficulties.<br>**Crisis plan.**<br>**Close-Out.** Farewell celebration.<br>**Awarding the participants. Certificates**: "You did your very best."<br>**Outlook to booster session.**	Experiencing acquired / activated resources (balloon game).<br>Discussing individual skills lists, open needs.<br>Discussing crisis plan / designing emergency cards.
**Booster Session**<br>Week 12<br>Session 17	**Review and consolidation:** Helpful skills, difficulties with skills practice within the last four weeks, current open needs.<br>**Small symbolic gift,** e.g. little gemstone	Outlook for future / participants' wishes / practicing how "to turn the tide".

Table 2: START Adolescents Program, modules 1-8 and booster session

Assessments

Psychometric instruments

For details of the instruments including psychometric properties please see supplementary file 2. All self-report assessments are applied in the study languages, all interviews are applied with the help of interpreters if indicated.

For peer review only - http://bmjopen.bmj.com/site/about/guidelines.xhtml

Inclusion and exclusion criteria: In- and exclusion criteria are assessed with the Essen Trauma Inventory for Children and Adolescents (ETI-CA; (33)), Clinical Global Impression Scale, severity of illness subscale (CGI-S; (34)), the Kiddie-Schedule for Affective Disorders and Schizophrenia for School-Age Children-Present and Lifetime version (K-SADS-PL; (35-37)), the Alcohol Use Disorders Identification Test (AUDIT; (38)), the Columbia-Suicide Severity Rating Scale (C-SSRS; (39)), the Test of Nonverbal Intelligence, Fourth Edition (TONI 4; (40)), and the standardized Questionnaire to Assess Endangerment to Others (39).

Primary endpoints: Psychological distress is assessed with the Perceived Stress Scale, 10-item version (PSS-10), a validated self-report questionnaire, that measures the intensity with which individuals appraise their daily life as stressful, unpredictable, uncontrollable, and overloaded. Reliability and validity for the Arabic, English and German version have been shown to be sufficient or good (41-44). Traumatic symptoms are assessed with the Impact of Event Scale-Revised (IES-R), a validated, self-report questionnaire evaluating traumatic symptoms on the subscales intrusion, avoidance, hyperarousal and on an overall measure of traumatic distress. Reliability and validity for the Arabic, English, and German version have been shown to be good (45-48).

Secondary endpoints: We assess trauma-related psychosocial impairment and DSM-5 PTSD criteria with the Child and Adolescent Trauma Screen-2 (CATS-2; (49)), trauma-related cognitions with the Posttraumatic Cognitions Inventory - child version (cPTCI; (50-52)) and frequency and intensity of nightmares with the Disturbing Dream and Nightmare Severity Index (DDNSI; (53, 54)). Emotion regulation is assessed with the 18-item version of the Difficulties in Emotion Regulation Scale (DERS-18; (55)), general psychopathology with the Strengths and Difficulties Questionnaire (SDQ; (56,

For peer review only - http://bmjopen.bmj.com/site/about/guidelines.xhtml

57)), and the Beck Depression Inventory, second edition (BDI-II; (58, 59)). Overall severity of illness is assessed with the CGI-S (34).

Additional psychometric assessments: We assess adverse life experiences related to migration with the adapted German version of the self-rated Post-Migration Living Difficulties Questionnaire (PMLD; (60, 61)), and therapy expectations with a self-designed questionnaire comprising three questions that are answered on a 5-point Likert-type scale.

Ambulatory momentary assessment: electronic diaries

We use smartphone-based electronic (e)-diaries to assess aversive inner tension, emotional intensity, trauma symptoms (intrusions, hypervigilance), cognitive and behavioral avoidance of triggering situations in daily environments, sleep quality, stressfulness, content and number of nightmares, self-efficacy, self-esteem, rumination and somatization (62-64). Filling in the answers takes a few minutes and items are available in all study languages. Rumination about cultural differences, social support and conflicts, discrimination experiences, and personally important events at school will be used as context variables to determine their associations with emotional distress and trauma symptoms. Participants receive a comprehensive explanation of the use of e-diaries as a spoken PowerPoint presentation and a written document in each participant's respective language. After the presentation, the participant is asked to do a test trial on the smartphone. Smartphones are programmed with the e-diary app movisensXS (65) and employ an hourly one-week time-based e-diary design. Prompts are pseudo-randomized within a time frame of 50-70 minutes to avoid expectancy effects. At the beginning of the study, participants are asked to enter their school schedules and determine their first prompt in the morning. On the weekends,

For peer review only
For peer review only - http://bmjopen.bmj.com/site/about/guidelines.xhtml

e-diary assessments start at 10 a.m., no data are assessed during school hours. The prompts end at about 9 p.m. on school days and about 10 p.m. if there is no school the next day.

Hair cortisol

If additional informed consent for hair cortisol collection is provided by study participants and their legal representatives, we collect thin hair strands from the posterior vertex of the head cut as close to the scalp as possible. Hair strands are tied together, sealed, and stored in a dry and dark place. Prior to analyses, they will be cut into a 2cm segment proximal to the scalp, representing cumulative cortisol secretion of the last two months (66).

Quality assurance and monitoring

The study procedures are monitored by the Coordination Center for Clinical Trials (KKS) Heidelberg according to the ICH-GCP (32) with respect to a risk-based quality management strategy and ensure that the trial is conducted according to protocol and regulatory requirements. All data of the ongoing study are reviewed by an independent data monitoring committee once a year with special focus on safety issues.

Manual adherence across different therapists and participating centers is ensured by training all therapists on the START Adolescents program and regular supervision with standardized assessments of manual adherence. We require a certain level of professional training from the therapists, of whom at least one per group must be a graduated psychologist, pedagogue, or physician.

The quality of psychometric assessments, tests, and interviews is ensured by training the raters on the study assessments. Raters must have a Bachelor's or Master's degree in psychology or a comparable standard. Raters with a Bachelor's degree in

For peer review only - http://bmjopen.bmj.com/site/about/guidelines.xhtml

psychology will administer questionnaires and psychological tests, raters with a Master's degree in psychology will administer the study interviews.

Data management

The KKS Heidelberg is responsible for data management and analysis as well as data security and data transfer processes. All procedures are implemented in accordance with ICH-GCP guidelines and the Declaration of Helsinki (32, 67). The protection of private data is ensured by a pseudonymization procedure, and all private data are handled with respect to the European General Data Protection Regulation (68). Data are assessed by an eCRF and paper-based self-report questionnaires. The latter are transferred in copy to the KKS Heidelberg for double entry into the eCRF. E-diary data are transferred to the Mental mHealth lab, Karlsruhe Institute of Technology (KIT), analyzed there and saved in encrypted form. The smartphones for e-diaries are provided by the research facility; no private smartphones are used for study purposes.

• Data availability

The research data generated during this study will be available upon reasonable request by the study coordinating center at the Department of Child and Adolescent Psychiatry, University Medical Center, Mainz, Germany. Anonymized data use by other researchers not involved in the study may be done with prior agreement.

Statistics

Sample size calculation

The sample size calculation is based on the co-primary outcomes of emotional distress (PSS-10) and traumatic symptoms (IES-R) at T1. Based on available studies (69, 70) we assume in our study an effect size of d=0.63 for the PSS-10 and of d=0.56 for the

For peer review only - http://bmjopen.bmj.com/site/about/guidelines.xhtml

IES-R score at T1. This results in a required sample size *of n*=84 (PSS-10) and *n*=104 (IES-R) for a one-sided test at an *α*=.025 level with a power of 80% to detect such differences. The START pilot trial showed a drop-out rate <10% and was conducted as a one-center single-arm naturalistic trial with mostly inpatients (20). We, therefore, calculated the sample size on the assumption of 40% drop-outs between T0 and T1 to account for the multi-center, waiting-list-controlled approach and unstable living conditions of the included population. This amounts to *n*=174 participants who need to be randomized in our trial.

Analysis population and analysis

All randomized participants will be included in the full-analysis set as allocated. All randomized participants, who finish the study according to the study protocol until T2 as planned with no missing values for T1 (primary endpoint) will be included in the per-protocol set for analysis as allocated. All randomized participants will be included in the safety population according to the applied intervention.

Baseline characteristics will be analyzed descriptively for the safety, full-analysis, and per-protocol set in the intervention and control group. The primary and co-primary endpoints will be analyzed in the full-analysis set. Missing values will be imputed. Endpoints will be assessed as response variables in linear mixed regression models with site and psychotherapeutic group as random effects, intervention group, sex, and region of origin as fixed effects, and age, PSS-10, and IES-R baseline scores, respectively, as linear effects with a hierarchical testing procedure that maintains the overall significance level of *α*=.025. The primary analysis will be repeated in the per-protocol population as a sensitivity analysis. Since the different timing of the T0 baseline examination in the study groups after randomization could cause biases in the effect estimates, that cannot be estimated or modeled, additional sensitivity

For peer review only - http://bmjopen.bmj.com/site/about/guidelines.xhtml

analyses will be performed, such as repeating the primary analysis using the screening value instead of the baseline value. Secondary outcomes will be analyzed in the full-analysis set. Missing values will not be imputed. The stability of treatment effects up to T3 will be assessed by calculating confidence intervals on the T1-T3 and T2-T3 difference for the PSS-10 and IES-R total scores in the intervention group. Psychometric scales used for assessment of secondary outcomes and hair cortisol concentrations will be analyzed according to primary endpoints or descriptively. For the analysis of e-diary data, multilevel models will be used to characterize momentary mechanisms and context-dependent affective experiences, which allow a nested data structure (momentary experiences within participants), and enable different numbers of ratings per participant to be handled quite well (71). Safety variables will be analyzed in the safety and full-analysis population.

Ethics and dissemination

The study protocol, patient recruitment procedures, patients' information, and informed consent material have been approved by the lead ethics committee of Rhineland-Palatinate (ID 2019-14709) and the ethics committees of participating sites. For every substantial protocol modification approval of all ethics committees is required. The study will be conducted according to ICH-GCP (32) and the Declaration of Helsinki (67) and has been registered at the German Clinical Trials Register (ID: DRKS00020771). Study results will be published through peer-reviewed publications and presented at scientific and clinical conferences.

Trial status

Recruitment started 1 October 2020.

For peer review only - http://bmjopen.bmj.com/site/about/guidelines.xhtml

Discussion

Evidence-based, evaluated, low-threshold, and culture-sensitive psychotherapeutic treatment programs, that reduce distress caused by traumatic experiences that can be efficiently established in routine mental health care, would represent valuable therapeutic interventions to support refugee minors with trauma-related symptoms and emotion regulation difficulties. However, such interventions are currently lacking. The primary aim of this study is to evaluate the clinical efficacy and intermediate outcome of the distress-reducing psychotherapeutic START program and to contribute to improved evidence-based therapeutic interventions for adolescent refugees within a stepped-care mental health approach in Germany. If shown to be effective, the first rigorously evaluated manualized intervention program for traumatized adolescent refugees will be available, which will have widespread implications for clinical practice. Some limitations for the application of the program in mental health routine care in host countries may be that the therapeutic study groups are limited only to traumatized refugees, which may not reflect clinical reality. Another limitation is the restriction of study participation to refugee minors with language skills in Arabic, Dari, English, German, and Somali. Thus, the study will not provide information on the effectiveness of the program if applied to refugee minors with a different cultural or language background. However, since all program materials are available in Arabic, Dari, English, German, and Somali, the START program will provide a treatment option not only in German- or English-speaking host countries that care for refugee minors but also in refugee camps based in Arabic-, Dari- or Somali-speaking countries, where is a huge need for easy-to-apply psychotherapeutic treatment options.

Beyond effects on the symptom level, we will analyze intervention effects on psychosocial functioning and everyday behavior through the use of e-diaries and on the neuroendocrine stress system by analyzing hair cortisol. The multimodal

For peer review only - http://bmjopen.bmj.com/site/about/guidelines.xhtml

assessment approach of symptoms, functioning, daily living outcomes, context variables, and neuroendocrine effects, as well as the follow-up assessment, will enable us to analyze immediate and intermediate treatment effects not only on the phenomenological clinical level but also on the biological and daily living level. This will extend our understanding of intervention effects, influencing factors and the course of trauma-related mental health problems in refugee minors.

Contributors

ES obtained the funding for the START adolescent project and the Consortium coordination, designed the study design for the trial and the structure of START Consortium. ES and FH drafted the first version of the manuscript and revised the manuscript several times. FH organizes the START Adolescents study. AD and EM developed the START intervention, were involved in the adaptation of the program for this study and revised the manuscript. AD heads the Idar-Oberstein site. EM heads the Kleinblittersdorf site. SKH and UEP designed the smartphone-based experience sampling study and critically revised the manuscript. UEP obtained funding for the experience sampling study and is the head of the Mental mHealth lab, KIT. CJM designed the protocol for the assessment of neuroendocrine parameters and critically revised the manuscript. TIA is the head of the Landau site and critically revised the manuscript. BP is the head of the Koblenz site and critically revised the manuscript. HC is the head of the Marburg site and critically revised the manuscript. DK was, SO and NF are involved in the coordination of this study and DK, SO and NF critically revised the manuscript. IB is responsible for biostatistics and critically revised the manuscript. MH coordinates the START consortium and was involved in critically revising the manuscript. All authors read and approved the final manuscript.

Funding and sponsor: With the exception of the hair cortisol assessments, which will be self-funded by the Department of Child and Adolescent Psychiatry and Psychotherapy, University Medical Center Mainz, Germany, the START Adolescents study is funded by the German Federal Ministry of Education and Research (BMBF); funding code: 01EF1807A. The trial sponsor is the University Medical Center Mainz, Langenbeckstraße 1 55131 Mainz, Germany, www.unimedizin-mainz.de. The sponsor played no part in the study design and writing this report.

Competing interests: All authors have completed the ICMJE uniform disclosure form. The first two authors, Esther Sobanski & Florian Hammerle, received article processing fees and travel grants for conferences from the Federal Ministry of Education and Research, Germany and are consultants of the Federal Ministry of Rhineland Palatinate for refugee issues. Esther Sobanski recieved funding for the START Adolescents study from the Federal Ministry of Education and Research, Germany. Tina In-Albon received a research grant from the Federal Ministry of Education and Research for research of non-suicidal self-injury in adolescents and is president of the German Society of Cognitive Behavioral Psychotherapy for Children and Adolescents (KJPVT e.V.), unpaid position. David R. Kolar received travel grants for conferences from the Federal Ministry of Education and Research, Germany. Michael Huss recieved funding for the START Adolescents study from the Federal Ministry of Education and Research, Germany. All other authors declare that they have no competing interests.

Provenance and peer review: Study protocol was externally peer reviewed.

For peer review only - http://bmjopen.bmj.com/site/about/guidelines.xhtml

Patient consent for publication: Not applicable.

Acknowledgements: We would like to acknowledge Sarah Mannion, MA (Cantab) for reviewing the English, Bianca Brückner for the help with the recruitment, and Christa Hellmann for the technical help when preparing the manuscript.

References

1. United Nations High Commissioner for Refugees. Global Trends Forced Displacement in 2019. Denmark: UNHCR; 2020.
2. Office for Migration and Refugees. Schlüsselzahlen Asyl 2019. Nürnberg: Bundesamt für Migration und Flüchtlinge; 2020.
3. Office for Migration and Refugees. Flüchtlinge (2019): Aktuelle Zahlen. 2019.
4. Höhne E, Swantje van der Meer A, Kamp-Becker I, Christiansen H. A systematic review of risk and protective factors of mental health in unaccompanied minor refugees. Eur Child Adolesc Psychiatry. 2020.
5. Müller LRF, Gossmann K, Hartmann F, Büter KP, Rosner R, Unterhitzenberger J. 1-year follow-up of the mental health and stress factors in asylum-seeking children and adolescents resettled in Germany. BMC public health. 2019;19(908):1-11.
6. Müller LRF, Büter KP, Rosner R, Unterhitzenberger J. Mental health and associated stress factors in accompanied and unaccompanied refugee minors resettled in Germany: a cross-sectional study. Child and adolescent psychiatry and mental health. 2019;13(1):1-13.
7. Walg M, Fink E, Großmeier M, Temprano M, Hapfelmeier G. Häufigkeit psychischer Störungen bei unbegleiteten minderjährigen Flüchtlingen in Deutschland. Zeitschrift für Kinder- und Jugendpsychiatrie und Psychotherapie. 2016;45:58-68.
8. Anders M, & Christiansen, H. . Die Versorgung unbegleiteter minderjähriger Flüchtlinge – eine systematische Literaturübersicht zu psychologischen Interventionen. Kindheit und Entwicklung. 2016;25(4):216-30.
9. Sierau S, Schneider E, Nesterko Y, von Klitzing K, Glaesmer H. Psychische Belastung bei unbegleiteten jungen Geflüchteten in Jugendhilfeeinrichtungen. Psychiatrische Praxis. 2019;46(03):135-40.
10. Kien C, Sommer I, Faustmann A, Gibson L, Schneider M, Krczal E, et al. Prevalence of mental disorders in young refugees and asylum seekers in European Countries: a systematic review. European child & adolescent psychiatry. 2019;28(10):1295-310.
11. Tam SY, Houlihan S, Melendez-Torres G. A systematic review of longitudinal risk and protective factors and correlates for posttraumatic stress and its natural history in forcibly displaced children. Trauma, Violence, & Abuse. 2017;18(4):377-95.
12. Barron IG, Abdallah G, Smith P. Randomized control trial of a CBT trauma recovery program in Palestinian schools. Journal of Loss and Trauma. 2013;18(4):306-21.
13. Tyrer RA, Fazel M. School and community-based interventions for refugee and asylum seeking children: a systematic review. PloS one. 2014;9(2):e89359.
14. Pfeiffer E, Goldbeck L. Evaluation of a trauma-focused group intervention for unaccompanied young refugees: A pilot study. Journal of Traumatic Stress. 2017;30(5):531-6.
15. Gormez V, Kılıç HN, Orengul AC, Demir MN, Mert EB, Makhlouta B, et al. Evaluation of a school-based, teacher-delivered psychological intervention group program for trauma-affected Syrian refugee children in Istanbul, Turkey. Psychiatry and Clinical Psychopharmacology. 2017;27(2):125-31.
16. Nocon A, Eberle-Sejari R, Unterhitzenberger J, Rosner R. The effectiveness of psychosocial interventions in war-traumatized refugee and internally displaced minors: systematic review and meta-analysis. European journal of psychotraumatology. 2017;8(sup2):1388709.

For peer review only - http://bmjopen.bmj.com/site/about/guidelines.xhtml

17. Demazure G, Gaultier S, Pinsault N. Dealing with difference: a scoping review of psychotherapeutic interventions with unaccompanied refugee minors. European child & adolescent psychiatry. 2018;27(4):447-66.
18. Dixius A, Möhler E. START–Entwicklung einer Intervention zur Erststabilisierung und Arousal-Modulation für stark belastete minderjährige Flüchtlinge. Praxis der Kinderpsychologie und Kinderpsychiatrie. 2017;66(4):277-86.
19. Dixius A, Möhler E. Ein neues Therapie-Konzept validiert die besonderen Bedürfnisse geflüchteter Kinder und Jugendlicher: START. Psychotherapie Forum. 2017;22(3):76-85.
20. Dixius A, Stevens A, Moehler E. A pilot evaluation study of an intercultural treatment program for stabilization and arousal modulation for intensely stressed children and adolescents and minor refugees, called START (Stress-Traumasymptoms-Arousal-Regulation-Treatment). ARC Journal of Psychiatry. 2017;2(2):7-24.
21. Dixius A, Möhler E. Feasibility and Effectiveness of a New Short-Term Psychotherapy Concept for Adolescents With Emotional Dysregulation. Frontiers in Psychiatry. 2021;11:1630.
22. Ebner-Priemer UW, Eid M, Kleindienst N, Stabenow S, Trull TJ. Analytic strategies for understanding affective (in) stability and other dynamic processes in psychopathology. Journal of abnormal psychology. 2009;118(1):195.
23. Forbes EE, Stepp SD, Dahl RE, Ryan ND, Whalen D, Axelson DA, et al. Real-world affect and social context as predictors of treatment response in child and adolescent depression and anxiety: an ecological momentary assessment study. Journal of Child and Adolescent Psychopharmacology. 2012;22(1):37-47.
24. Peeters F, Berkhof J, Rottenberg J, Nicolson NA. Ambulatory emotional reactivity to negative daily life events predicts remission from major depressive disorder. Behaviour research and therapy. 2010;48(8):754-60.
25. Wichers M, Lothmann C, Simons CJ, Nicolson NA, Peeters F. The dynamic interplay between negative and positive emotions in daily life predicts response to treatment in depression: a momentary assessment study. British Journal of Clinical Psychology. 2012;51(2):206-22.
26. Wichers M, Peeters F, Geschwind N, Jacobs N, Simons C, Derom C, et al. Unveiling patterns of affective responses in daily life may improve outcome prediction in depression: a momentary assessment study. Journal of affective disorders. 2010;124(1-2):191-5.
27. Santangelo PS, Reinhard I, Koudela-Hamila S, Bohus M, Holtmann J, Eid M, et al. The temporal interplay of self-esteem instability and affective instability in borderline personality disorder patients' everyday lives. Journal of abnormal psychology. 2017;126(8):1057-65.
28. Steudte-Schmiedgen S, Kirschbaum C, Alexander N, Stalder T. An integrative model linking traumatization, cortisol dysregulation and posttraumatic stress disorder: Insight from recent hair cortisol findings. Neuroscience & Biobehavioral Reviews. 2016;69:124-35.
29. Mayer A, Taubner S, Bark C, Holl J. Herausforderungen in der frühpädagogischen Arbeit mit geflüchteten Familien mentalisierungsbasiert begegnen. Praxis der Kinderpsychologie und Kinderpsychiatrie. 2019;68(8):711-27.
30. Chan A-W, Tetzlaff JM, Altman DG, Dickersin K, Moher D. SPIRIT 2013: new guidance for content of clinical trial protocols. Lancet. 2013;381(9861).
31. Chan A-W, Tetzlaff JM, Altman DG, Laupacis A, Gøtzsche PC, Krleža-Jerić K, et al. SPIRIT 2013 statement: defining standard protocol items for clinical trials. Annals of internal medicine. 2013;158(3):200-7.
32. ICH Expert Working Group. ICH Harmonised Tripartite Guideline for good clinical practice E6 (R1). ICH Harmon Tripart Guidel. 1996;1996(4).
33. Graham FS, W, Tagay S. ETI-CA Essen Trauma-Inventory for Children and Adolescents - Interview Essen: LVR-Klinikum Essen, Universität Duisburg; 2012.
34. Guy W. Clinical global impression. Assessment manual for Psychopharmacology. 1976:217-22.
35. Kaufman J, Birmaher B, Brent D, Rao U, Flynn C, Moreci P, et al. Schedule for affective disorders and schizophrenia for school-age children-present and lifetime version (K-SADS-PL): initial reliability and validity data. Journal of the American Academy of Child & Adolescent Psychiatry. 1997;36(7):980-8.

For peer review only

For peer review only - http://bmjopen.bmj.com/site/about/guidelines.xhtml

36. American Psychiatric Association. Diagnostic and Statistical Manual of Mental Disorders. 5th ed. Washington, DC2013.
37. Kaufman J, Birmaher B, Axelson D, Perepletchikova F, Brent D, Ryan N. Schedule for affective and disorders and schizophrenia for school aged children (6–18 years): Kiddie-SADS-lifetime version (K-SADS-PL DSM 5). Pittsburgh, PA: Western Psychiatric Institute and Clinic; 2016.
38. National Institute on Drug Abuse. Audit. 2013.
39. Posner K, Brown GK, Stanley B, Brent DA, Yershova KV, Oquendo MA, et al. The Columbia–Suicide Severity Rating Scale: initial validity and internal consistency findings from three multisite studies with adolescents and adults. American journal of psychiatry. 2011;168(12):1266-77.
40. Brown L, Sherbenou RJ, Johnsen SK. Test of nonverbal intelligence: TONI-4: Pro-ed Austin, TX; 2010.
41. Cohen S, Williamson G. Perceived stress in a probability sample of the United States. In: Spacapan S, & Oskamp, S., editor. The Social Psychology of Health: Claremont Symposium on Applied Social Psychology Newbury Park, CA: Sage; 1988. p. 31-67.
42. Klein EM, Brähler E, Dreier M, Reinecke L, Müller KW, Schmutzer G, et al. The German version of the Perceived Stress Scale–psychometric characteristics in a representative German community sample. BMC psychiatry. 2016;16(1):1-10.
43. Musial F, Büssing A, Heusser P, Choi K-E, Ostermann T. Mindfulness-based stress reduction for integrative cancer care–a summary of evidence. Complementary Medicine Research. 2011;18(4):192-202.
44. Chaaya M, Osman H, Naassan G, Mahfoud Z. Validation of the Arabic version of the Cohen Perceived Stress Scale (PSS-10) among pregnant and postpartum women. BMC psychiatry. 2010;10(1):111.
45. Weiss DS, Marmar CR. The Impact of Event Scale-Revised. Assessing psychological trauma and PTSD. New York, NY, US: The Guilford Press; 1997. p. 399-411.
46. Weiss DS. The Impact of Event Scale-Revised. Assessing psychological trauma and PTSD, 2nd ed. New York, NY, US: The Guilford Press; 2004. p. 168-89.
47. Maercker A, Schützwohl M. Erfassung von psychischen Belastungsfolgen: Die Impact of Event Skala-revidierte Version (IES-R). Diagnostica. 1998.
48. Davey C, Heard R, Lennings C. Development of the Arabic versions of the Impact of Events Scale-Revised and the Posttraumatic Growth Inventory to assess trauma and growth in Middle Eastern refugees in Australia. Clinical Psychologist. 2015;19(3):131-9.
49. Sachser C, Berliner L, Holt T, Jensen TK, Jungbluth N, Risch E, et al. International development and psychometric properties of the Child and Adolescent Trauma Screen (CATS). Journal of affective disorders. 2017;210:189-95.
50. Meiser-Stedman R, Smith P, Bryant R, Salmon K, Yule W, Dalgleish T, et al. Development and validation of the child post-traumatic cognitions inventory (CPTCI). Journal of Child Psychology and Psychiatry. 2009;50(4):432-40.
51. de Haan A, Petermann F, Meiser-Stedman R, Goldbeck L. Psychometric properties of the German version of the child post-traumatic cognitions inventory (CPTCI-GER). Child Psychiatry & Human Development. 2016;47(1):151-8.
52. Meiser-Stedman R. Post-traumatic Cognitions Inventory - Child Version (Arabic version) n. d. [Available from: https://www.childrenandwar.org/wp-content/uploads/2019/06/CPTCI_arabisch.pdf].
53. Krakow B. Nightmare Complaints in Treatment-Seeking Patients in Clinical Sleep Medicine Settings: Diagnostic and Treatment Implications. Sleep. 2006;29:1313-9.
54. Krakow B, Haynes PL, Warner TD, Santana E, Melendrez D, Johnston L, et al. Nightmares, insomnia, and sleep-disordered breathing in fire evacuees seeking treatment for posttraumatic sleep disturbance. Journal of Traumatic Stress: Official Publication of The International Society for Traumatic Stress Studies. 2004;17(3):257-68.
55. Victor SE, Klonsky ED. Validation of a brief version of the difficulties in emotion regulation scale (DERS-18) in five samples. Journal of psychopathology and Behavioral Assessment. 2016;38(4):582-9.

For peer review only - http://bmjopen.bmj.com/site/about/guidelines.xhtml

56. Goodman R. Psychometric properties of the strengths and difficulties questionnaire. Journal of the American Academy of Child & Adolescent Psychiatry. 2001;40(11):1337-45.
57. Idzelis M, Ali AS, Gaaddasaar M, Bogoslaw LH, Ulstad K, Tanaka A. n. d. [Available from: https://www.sdqinfo.org/py/sdqinfo/b3.py?language=Somali].
58. Beck AT, Steer RA, Brown GK. Bdi-ii manual. 1996.
59. Selmo P, Koch T, Brand J, Wagner B, Knaevelsrud C. Psychometric properties of the online Arabic versions of BDI-II, HSCL-25, and PDS. European Journal of Psychological Assessment. 2016;35:46-54.
60. Zentrum Überleben. n. d. [Available from: https://www.ueberleben.org/].
61. Refugio Munich. n. d. [Available from: https://www.refugio-muenchen.de/].
62. Pfaltz MC, Michael T, Meyer AH, Wilhelm FH. Reexperiencing symptoms, dissociation, and avoidance behaviors in daily life of patients with PTSD and patients with panic disorder with agoraphobia. Journal of traumatic stress. 2013;26(4):443-50.
63. Price M, Ruggiero KJ, Ferguson PL, Patel SK, Treiber F, Couillard D, et al. A feasibility pilot study on the use of text messages to track PTSD symptoms after a traumatic injury. General Hospital Psychiatry. 2014;36(3):249-54.
64. Ruscio AM, Gentes EL, Jones JD, Hallion LS, Coleman ES, Swendsen J. Rumination predicts heightened responding to stressful life events in major depressive disorder and generalized anxiety disorder. Journal of Abnormal Psychology. 2015;124(1):17-26.
65. movisens GmbH. 2021 [Available from: https://www.movisens.com/de/].
66. Short SJ, Stalder T, Marceau K, Entringer S, Moog NK, Shirtcliff EA, et al. Correspondence between hair cortisol concentrations and 30-day integrated daily salivary and weekly urinary cortisol measures. Psychoneuroendocrinology. 2016;71:12-8.
67. World Medical Association. Declaration of Helsinki: Ethical Principles for Medical Research Involving Human Subjects. JAMA. 2013;310(20):2191-4.
68. General Data Protection Regulation GDPR. n. d. [Available from: https://gdpr-info.eu/].
69. Prasad L, Varrey A, Sisti G. Medical students' stress levels and sense of well being after six weeks of yoga and meditation. Evidence-Based Complementary and Alternative Medicine. 2016;2016.
70. Butollo W, Karl R, König J, Rosner R. A randomized controlled clinical trial of dialogical exposure therapy versus cognitive processing therapy for adult outpatients suffering from PTSD after type I trauma in adulthood. Psychotherapy and Psychosomatics. 2016;85(1):16-26.
71. Raudenbush SW, Bryk AS. Hierarchical linear models: Applications and data analysis methods: Sage Publications; 2002.

For peer review only

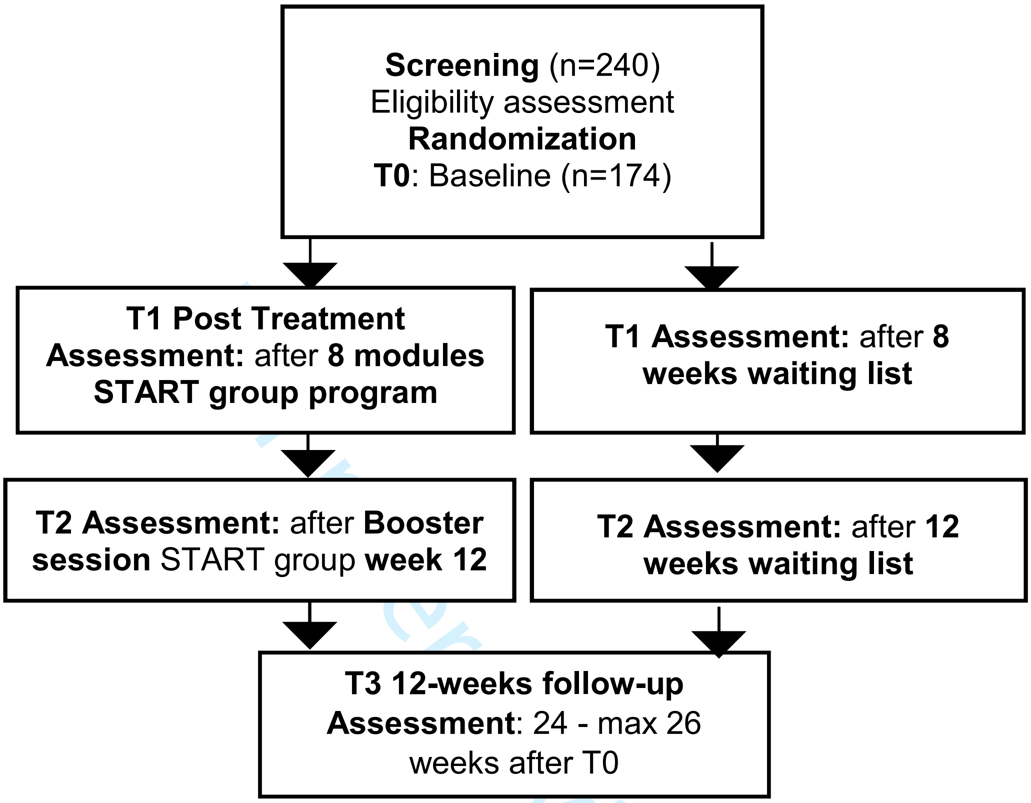
Screening (n=240)
Eligibility assessment
Randomization
T0: Baseline (n=174)
T1 Post Treatment Assessment: after **8 modules START group program**
T1 Assessment: after **8 weeks waiting list**
T2 Assessment: after **Booster session** START group **week 12**
T2 Assessment: after **12 weeks waiting list**
T3 12-weeks follow-up Assessment: 24 - max 26 weeks after T0

Reporting checklist for protocol of a clinical trial.

SPIRIT 2013 Checklist: Recommended items to address in a clinical trial protocol and related documents

		Reporting Item	Page Number
**Administrative information**			
Title	#1	Descriptive title identifying the study design, population, interventions, and, if applicable, trial acronym	1
Trial registration	#2a	Trial identifier and registry name. If not yet registered, name of intended registry	3
Trial registration: data set	#2b	All items from the World Health Organization Trial Registration Data Set	3
Protocol version	#3	Date and version identifier	6
Funding	#4	Sources and types of financial, material, and other support	21
Roles and responsibilities: contributorship	#5a	Names, affiliations, and roles of protocol contributors	1, 21
Roles and responsibilities: sponsor contact information	#5b	Name and contact information for the trial sponsor	21
Roles and responsibilities: sponsor and funder	#5c	Role of study sponsor and funders, if any, in study design; collection, management, analysis, and interpretation of data; writing of the report; and the decision to submit the report for publication, including whether they will have ultimate authority over any of these activities	21

For peer review only

Roles and responsibilities: committees	#5d	Composition, roles, and responsibilities of the coordinating centre, steering committee, endpoint adjudication committee, data management team, and other individuals or groups overseeing the trial, if applicable (see Item 21a for data monitoring committee)	7, 16ff
**Introduction**			
Background and rationale	#6a	Description of research question and justification for undertaking the trial, including summary of relevant studies (published and unpublished) examining benefits and harms for each intervention	4ff
Background and rationale: choice of comparators	#6b	Explanation for choice of comparators	---
Objectives	#7	Specific objectives or hypotheses	5,6
Trial design	#8	Description of trial design including type of trial (eg, parallel group, crossover, factorial, single group), allocation ratio, and framework (eg, superiority, equivalence, non-inferiority, exploratory)	7
**Methods: Participants, interventions, and outcomes**			
Study setting	#9	Description of study settings (eg, community clinic, academic hospital) and list of countries where data will be collected. Reference to where list of study sites can be obtained	7<br>Referen ce: DRKS00 020771
Eligibility criteria	#10	Inclusion and exclusion criteria for participants. If applicable, eligibility criteria for study centres and individuals who will perform the interventions (eg, surgeons, psychotherapists)	8,16

For peer review only

Interventions: description	#11a	Interventions for each group with sufficient detail to allow replication, including how and when they will be administered	11ff., table 2
Interventions: modifications	#11b	Criteria for discontinuing or modifying allocated interventions for a given trial participant (eg, drug dose change in response to harms, participant request, or improving / worsening disease)	12
Interventions: adherance	#11c	Strategies to improve adherence to intervention protocols, and any procedures for monitoring adherence (eg, drug tablet return; laboratory tests)	16
Interventions: concomitant care	#11d	Relevant concomitant care and interventions that are permitted or prohibited during the trial	8
Outcomes	#12	Primary, secondary, and other outcomes, including the specific measurement variable (eg, systolic blood pressure), analysis metric (eg, change from baseline, final value, time to event), method of aggregation (eg, median, proportion), and time point for each outcome. Explanation of the clinical relevance of chosen efficacy and harm outcomes is strongly recommended	5,6, 13-16, 18ff, Figure 1, Table 1
Participant timeline	#13	Time schedule of enrolment, interventions (including any run-ins and washouts), assessments, and visits for participants. A schematic diagram is highly recommended (see Figure)	9-12, table 1
Sample size	#14	Estimated number of participants needed to achieve study objectives and how it was determined, including clinical and statistical assumptions supporting any sample size calculations	17,18
Recruitment	#15	Strategies for achieving adequate participant enrolment to reach target sample size	8, 9

Methods: Assignment of interventions (for controlled trials)

For peer review only

Allocation: sequence generation	#16a	Method of generating the allocation sequence (eg, computer-generated random numbers), and list of any factors for stratification. To reduce predictability of a random sequence, details of any planned restriction (eg, blocking) should be provided in a separate document that is unavailable to those who enrol participants or assign interventions	9
Allocation concealment mechanism	#16b	Mechanism of implementing the allocation sequence (eg, central telephone; sequentially numbered, opaque, sealed envelopes), describing any steps to conceal the sequence until interventions are assigned	9
Allocation: implementation	#16c	Who will generate the allocation sequence, who will enrol participants, and who will assign participants to interventions	9
Blinding (masking)	#17a	Who will be blinded after assignment to interventions (eg, trial participants, care providers, outcome assessors, data analysts), and how	7
Blinding (masking): emergency unblinding	#17b	If blinded, circumstances under which unblinding is permissible, and procedure for revealing a participant's allocated intervention during the trial	----
**Methods: Data collection, management, and analysis**			
Data collection plan	#18a	Plans for assessment and collection of outcome, baseline, and other trial data, including any related processes to promote data quality (eg, duplicate measurements, training of assessors) and a description of study instruments (eg, questionnaires, laboratory tests) along with their reliability and validity, if known. Reference to where data collection forms can be found, if not in the protocol	9, 13ff, 16ff Table1 Addition al table2
Data collection plan: retention	#18b	Plans to promote participant retention and complete follow-up, including list of any outcome data to be	8

For peer review only

		collected for participants who discontinue or deviate from intervention protocols	
Data management	#19	Plans for data entry, coding, security, and storage, including any related processes to promote data quality (eg, double data entry; range checks for data values). Reference to where details of data management procedures can be found, if not in the protocol	16ff
Statistics: outcomes	#20a	Statistical methods for analysing primary and secondary outcomes. Reference to where other details of the statistical analysis plan can be found, if not in the protocol	17ff
Statistics: additional analyses	#20b	Methods for any additional analyses (eg, subgroup and adjusted analyses)	17ff
Statistics: analysis population and missing data	#20c	Definition of analysis population relating to protocol non-adherence (eg, as randomised analysis), and any statistical methods to handle missing data (eg, multiple imputation)	18
**Methods: Monitoring**			
Data monitoring: formal committee	#21a	Composition of data monitoring committee (DMC); summary of its role and reporting structure; statement of whether it is independent from the sponsor and competing interests; and reference to where further details about its charter can be found, if not in the protocol. Alternatively, an explanation of why a DMC is not needed	16
Data monitoring: interim analysis	#21b	Description of any interim analyses and stopping guidelines, including who will have access to these interim results and make the final decision to terminate the trial	7
Harms	#22	Plans for collecting, assessing, reporting, and managing solicited and spontaneously reported adverse events and other unintended effects of trial interventions or trial conduct	9, 10, 12 table 1

For peer review only

Auditing	#23	Frequency and procedures for auditing trial conduct, if any, and whether the process will be independent from investigators and the sponsor	16
**Ethics and dissemination**			
Research ethics approval	#24	Plans for seeking research ethics committee / institutional review board (REC / IRB) approval	19
Protocol amendments	#25	Plans for communicating important protocol modifications (eg, changes to eligibility criteria, outcomes, analyses) to relevant parties (eg, investigators, REC / IRBs, trial participants, trial registries, journals, regulators)	19
Consent or assent	#26a	Who will obtain informed consent or assent from potential trial participants or authorised surrogates, and how (see Item 32)	9
Consent or assent: ancillary studies	#26b	Additional consent provisions for collection and use of participant data and biological specimens in studies, if applicable	15,16
Confidentiality	#27	How personal information about potential and enrolled participants will be collected, shared, and maintained in order to protect confidentiality before, during, and after the trial	17
Declaration of interests	#28	Financial and other competing interests for principal investigators for the overall trial and each study site	21
Data access	#29	Statement of who will have access to the final trial dataset, and disclosure of contractual agreements that limit such access for investigators	17
Ancillary and post trial care	#30	Provisions, if any, for ancillary and post-trial care, and for compensation to those who suffer harm from trial participation	8, 9
Dissemination policy: trial results	#31a	Plans for investigators and sponsor to communicate trial results to participants, healthcare professionals, the public, and other relevant groups (eg, via publication,	19

For peer review only

		reporting in results databases, or other data sharing arrangements), including any publication restrictions	
Dissemination policy: authorship	#31b	Authorship eligibility guidelines and any intended use of professional writers	--
Dissemination policy: reproducible research	#31c	Plans, if any, for granting public access to the full protocol, participant-level dataset, and statistical code	--
**Appendices**			
Informed consent materials	#32	Model consent form and other related documentation given to participants and authorised surrogates	Additional document
Biological specimens	#33	Plans for collection, laboratory evaluation, and storage of biological specimens for genetic or molecular analysis in the current trial and for future use in ancillary studies, if applicable	--

None The SPIRIT Explanation and Elaboration paper is distributed under the terms of the Creative Commons Attribution License CC-BY-NC. This checklist can be completed online using https://www.goodreports.org/, a tool made by the EQUATOR Network in collaboration with Penelope.ai

For peer review only

Psychometric instruments

If not available psychometric properties for internal consistency and reliability, criterion validity and convergent validity will be calculated within our sample.

Inclusion and exclusion criteria

Essen Trauma Inventory for Children and Adolescents

The Essen Trauma Inventory for Children and Adolescents (ETI-CA; (1, 2)) comprises a list of traumatic events and assesses symptoms of acute adjustment disorder and PTSD according to DSM-IV criteria (3) within the last four weeks, in relation to the most disturbing traumatic event if more than one was experienced. It further assesses the degree of impairment in six areas of daily living (e.g., school, peers) due to the traumatic event. It is available as a self-report questionnaire and an interview version, with the latter being used in this study. ETI-CA has been designed for the age group 12-17 years and is available in German and English. If necessary, we will translate the ETI-CA interview into Arabic, Dari, and Somali.

Clinical Global Impression Scale – Severity of illness

The Clinical Global Impression Scale is an internationally used instrument and yields the three different measures: 1. Severity of illness (CGI-S), 2. Global improvement (CGI-I), and 3. Efficacy index (4). In our study, we will apply the CGI-S, which is a 7-point scale on which the clinician rates the severity of the patient's illness at the time of assessment relative to the clinician's past experience with patients with the same diagnosis. Possible ratings range from 1=not ill at all to 7=among the most extremely ill patients. The German version of the scale (4) is clinically rated by study staff.

Kiddie-Schedule for Affective Disorders and Schizophrenia, Present and Lifetime

For peer review only - http://bmjopen.bmj.com/site/about/guidelines.xhtml

The Schedule for Affective Disorders and Schizophrenia for School-Age Children-Present and Lifetime version (K-SADS-PL) is a widely used, validated, semi-structured diagnostic interview for the standardized assessment of mental disorders according to DSM-5 in children and adolescents aged 6-18 years (5-7). For the purpose of this study we will use the K-SASDS-PL diagnostic screening interview with subsequent diagnostic interviews if indicated, and the summary lifetime diagnosis checklist. The diagnostic screening interview reviews the most severe current and past major symptoms of all diagnoses assessed with the K-SADS-PL. If the participant displays indicative symptoms of a specific disorder according to the screening, the referring diagnostic interviews will be applied. The K-SADS-PL comprises the following diagnostic interviews: depressive and bipolar-related disorders, psychotic disorders, anxiety disorders, obsessive-compulsive disorders, trauma-related disorders including PTSD, externalizing disorders, eating disorders, and substance abuse disorders. The summary lifetime diagnosis checklist then summarizes all mental diagnoses assessed by the K-SADS-PL. The K-SADS-PL is available in German and English for the purpose of the study If needed, the interview is conducted with the assistance of an interpreter.

Alcohol Use Disorders Identification Test

The Alcohol Use Disorders Identification Test (AUDIT) is an internationally used, validated, self-report questionnaire approved by the World Health Organization to screen individuals for hazardous or harmful alcohol consumption and alcohol dependence. It can be applied from age 14 years onwards. Items are rated on a 5-point Likert scale (0-4). Total possible AUDIT scores range from 0-40. Scores of 8-10 or more are indicative of hazardous, harmful alcohol use or possible alcohol dependence. The AUDIT is freely available in many languages, including Arabic,

For peer review only - http://bmjopen.bmj.com/site/about/guidelines.xhtml

English and German (8). For the purpose of our study, we translated the questionnaire into Dari and Somali.

Columbia-Suicide Severity Rating Scale

The Columbia-Suicide Severity Rating Scale (C-SSRS) assesses suicidal ideation and behavior. Questions are phrased for use in an interview format. The C-SSRS measures the following constructs: severity of suicidal ideation, intensity of suicidal ideation, suicidal and/or self-harm behavior, and lethality of suicide attempts. It is provided in a lifetime and recent/since last visit version (9). We use the lifetime version for the first assessment at the screening visit and the recent/since last visit version for all other study visits. In addition to the original validated English version, the C-SSRS is available in a German version and has been translated into Arabic, Dari, and Somali.

Test of Nonverbal Intelligence, fourth edition

The Test of Nonverbal Intelligence, Fourth Edition (TONI 4) is a language-free, validated intelligence test that requires no reading, writing or speaking on the examinee's part. It can be used from the age of six years onwards. Test items present a variety of problem-solving tasks in ascending order of difficulty. The abstract, figural content of the test items reduces the cultural loading of the test and the result is not influenced by insufficient language skills (10).

Questionnaire to Assess Endangerment to Others

The standardized Questionnaire to Assess Endangerment to Others is a self-designed, self-rated questionnaire in accordance with the wording of the C-SSRS (9), which assesses thoughts or behaviors within the last six months displaying endangerment or threat to others. The questionnaire comprises six questions that are answered in a yes/no structure and is provided in Arabic, Dari, English, German and Somali.

For peer review only - http://bmjopen.bmj.com/site/about/guidelines.xhtml

Primary endpoints

Perceived Stress Scale, 10-item version

The Perceived Stress Scale, 10-item version (PSS-10), is a validated self-report questionnaire that assesses psychological distress during the preceding month. It measures the intensity with which individuals appraise their daily life as stressful, unpredictable, uncontrollable, and overloaded. The PSS-10 was designed for use in participants aged 14 years or older. Items are rated on a 6-point scale from 0=never to 5=often. In addition to the original English version (11), a validated German version (12) and an Arabic version are available (13). For the purpose of this study, we translated the PSS-10 into Dari and Somali.

Impact of Event Scale-Revised

The Impact of Event Scale-Revised (IES-R) is a validated, self-report questionnaire assessing traumatic symptoms on three subscales: intrusion (intrusive thoughts, nightmares, intrusive feelings and imagery, dissociative-like re-experiencing), avoidance (numbing of responsiveness, avoidance of feelings, situations and ideas), hyperarousal (anger, irritability, hypervigilance, difficulty concentrating, heightened startle), and an overall measure of traumatic distress. Items are rated on a 5-point Likert scale from 0=not at all to 4=extreme. In addition to the original English version (14, 15), a validated German version (16) and an Arabic version (17) are available. For the purpose of this study, we translated the IES-R into Dari and Somali.

Secondary endpoints

Trauma symptoms

Child and Adolescent Trauma Screen-2

The Child and Adolescent Trauma Screen-2 (CATS-2) is a validated, freely accessible self-report questionnaire based on the DSM-5 PTSD criteria. It assesses DSM-5

For peer review only - http://bmjopen.bmj.com/site/about/guidelines.xhtml

criteria A (experience of a traumatic event ever), B (intrusion, re-experiencing), C (avoidance), D (negative alterations in cognitions and mood), and E (hyperarousal). Criteria B-D are rated on a 4-point scale (0=never, 3=almost always). Trauma-related psychosocial impairment (peers, family, school/work, hobbies, well-being) is assessed with yes/no items. For the purpose of this study, we use the validated English and German CATS-2 self-report versions (18) and translated the scale into Arabic, Dari and Somali.

Posttraumatic Cognitions Inventory - child version

The Posttraumatic Cognitions Inventory - child version (cPTCI) is a validated, self-report questionnaire assessing trauma-related cognitions on the subscales 'permanent and disturbing change', 'fragile person in a scary world' and on an overall score. Items are rated on a 5-point Likert-type scale and are summed to form a total score and the two subscales scores. For the purpose of this study, we use the validated English version (19), a validated German (20) and an Arabic cPTCI version (21) and translated the cPTCI into Dari and Somali.

Disturbing Dream and Nightmare Severity Index

The Disturbing Dream and Nightmare Severity Index (DDNSI) is a validated, self-report questionnaire and assesses number of nights with nightmares per week (0-7 nights), total number of nightmares per week, severity of nightmares, intensity of nightmares (Likert-type scale: 0=no problem to 6=extremely severe problem), how often nightmares result in awakenings (0=never/rarely to 4=always). The scale provides five sub-scores and a total score (22, 23). For the purpose of this study, the DDNSI was translated into Arabic, Dari, German, and Somali.

Emotion Regulation

Difficulties in Emotion Regulation Scale

For peer review only - http://bmjopen.bmj.com/site/about/guidelines.xhtml

The 18-item version of the Difficulties in Emotion Regulation Scale (DERS-18; (24)) is a validated, self-report questionnaire assessing the following six components of emotion dysregulation on a 5-point Likert-type scale: 1) Nonacceptance of emotional responses, 2) Difficulties engaging in goal-directed behavior, 3) Impulse control difficulties, 4) Lack of emotional awareness, 5) Limited access to emotion regulation strategies, 6) Lack of emotional clarity. It can be used from the age of 13 years onwards and yields a total score as well as subscale scores. For the purpose of the study, we use the 18 items from the unpublished German validation study of the DERS-36 (25, 26) and translated the English version of the DERS-18 (24) into Arabic, Dari and Somali.

General Psychopathology

Strengths and Difficulties Questionnaire

The Strengths and Difficulties Questionnaire (SDQ) is a widely used, validated parent-/caregiver-rated and/or self-report screening questionnaire for children and adolescents that is freely available online in an English version (27), German version (28), Arabic version (29), and Somali parent/caregiver version (30). The SDQ screens for difficulties in four areas: 1) emotional symptoms, 2) conduct problems, 3) hyperactivity/inattention, and 4) peer relationship problems. It additionally assesses strengths in prosocial behavior and provides a total score that sums up all subscale scores. For the purpose of this study, we use the available Arabic, English, German, self-report, and caregiver SDQ as well as the Somali caregiver version, and translated the SDQ caregiver and self-report version into Dari and the self-report version into Somali.

Beck Depression Inventory, second edition

The Beck Depression Inventory, second edition (BDI-II) is a widely used, validated, self-report questionnaire that assesses the severity of depression according to DSM-

For peer review only - http://bmjopen.bmj.com/site/about/guidelines.xhtml

IV criteria on a 4-point Likert-like scale. It can be used in adolescents from age 13 years onwards. The BDI-II is available in a validated English (31), validated German (32) and a validated Arabic version (33), which we use in this study as well as a translated Dari and Somali version

References

1. Graham FS, W, Tagay S. ETI-CA Essen Trauma-Inventory for Children and Adolescents - Interview Essen: LVR-Klinikum Essen, Universität Duisburg; 2012.
2. Tagay S, Düllmann S, Hermans E, Repic N, Hiller R, Senf W. Das Essener Trauma-Inventar für Kinder und Jugendliche (ETI-KJ). Zeitschrift für Kinder-und Jugendpsychiatrie und Psychotherapie. 2011;39:323-40.
3. American Psychiatric Association. Diagnostic and Statistical Manual of Mental Disorders (DSM-IV) Fourth Edition. Washington, DC: American Psychiatric Association; 1994.
4. Guy W. Clinical global impression. Assessment manual for Psychopharmacology. 1976:217-22.
5. Kaufman J, Birmaher B, Brent D, Rao U, Flynn C, Moreci P, et al. Schedule for affective disorders and schizophrenia for school-age children-present and lifetime version (K-SADS-PL): initial reliability and validity data. Journal of the American Academy of Child & Adolescent Psychiatry. 1997;36(7):980-8.
6. American Psychiatric Association. Diagnostic and Statistical Manual of Mental Disorders. 5th ed. Washington, DC2013.
7. Kaufman J, Birmaher B, Axelson D, Perepletchikova F, Brent D, Ryan N. Schedule for affective and disorders and schizophrenia for school aged children (6–18 years): Kiddie-SADS-lifetime version (K-SADS-PL DSM 5). Pittsburgh, PA: Western Psychiatric Institute and Clinic; 2016.
8. National Institute on Drug Abuse. Audit. 2013.
9. Posner K, Brown GK, Stanley B, Brent DA, Yershova KV, Oquendo MA, et al. The Columbia–Suicide Severity Rating Scale: initial validity and internal consistency findings from three multisite studies with adolescents and adults. American journal of psychiatry. 2011;168(12):1266-77.
10. Brown L, Sherbenou RJ, Johnsen SK. Test of nonverbal intelligence: TONI-4: Pro-ed Austin, TX; 2010.
11. Cohen S, Williamson G. Perceived stress in a probability sample of the United States. In: Spacapan S, & Oskamp, S., editor. The Social Psychology of Health: Claremont Symposium on Applied Social Psychology Newbury Park, CA: Sage; 1988. p. 31-67.
12. Klein EM, Brähler E, Dreier M, Reinecke L, Müller KW, Schmutzer G, et al. The German version of the Perceived Stress Scale–psychometric characteristics in a representative German community sample. BMC psychiatry. 2016;16(1):1-10.
13. Chaaya M, Osman H, Naassan G, Mahfoud Z. Validation of the Arabic version of the Cohen Perceived Stress Scale (PSS-10) among pregnant and postpartum women. BMC psychiatry. 2010;10(1):111.
14. Weiss DS, Marmar CR. The Impact of Event Scale—Revised. Assessing psychological trauma and PTSD. New York, NY, US: The Guilford Press; 1997. p. 399-411.
15. Weiss DS. The Impact of Event Scale-Revised. Assessing psychological trauma and PTSD, 2nd ed. New York, NY, US: The Guilford Press; 2004. p. 168-89.
16. Maercker A, Schützwohl M. Erfassung von psychischen Belastungsfolgen: Die Impact of Event Skala-revidierte Version (IES-R). Diagnostica. 1998.
17. Davey C, Heard R, Lennings C. Development of the A rabic versions of the I mpact of E vents S cale-R evised and the P osttraumatic G rowth I nventory to assess trauma and growth in M iddle E astern refugees in A ustralia. Clinical Psychologist. 2015;19(3):131-9.

For peer review only - http://bmjopen.bmj.com/site/about/guidelines.xhtml

18. Sachser C, Berliner L, Holt T, Jensen TK, Jungbluth N, Risch E, et al. International development and psychometric properties of the Child and Adolescent Trauma Screen (CATS). Journal of affective disorders. 2017;210:189-95.
19. Meiser-Stedman R, Smith P, Bryant R, Salmon K, Yule W, Dalgleish T, et al. Development and validation of the child post-traumatic cognitions inventory (CPTCI). Journal of Child Psychology and Psychiatry. 2009;50(4):432-40.
20. de Haan A, Petermann F, Meiser-Stedman R, Goldbeck L. Psychometric properties of the German version of the child post-traumatic cognitions inventory (CPTCI-GER). Child Psychiatry & Human Development. 2016;47(1):151-8.
21. Meiser-Stedman R. Post-traumatic Cognitions Inventory - Child Version (Arabic version) n. d. [Available from: https://www.childrenandwar.org/wp-content/uploads/2019/06/CPTCI_arabisch.pdf.
22. Krakow B. Nightmare Complaints in Treatment-Seeking Patients in Clinical Sleep Medicine Settings: Diagnostic and Treatment Implications. Sleep. 2006;29:1313-9.
23. Krakow B, Haynes PL, Warner TD, Santana E, Melendrez D, Johnston L, et al. Nightmares, insomnia, and sleep-disordered breathing in fire evacuees seeking treatment for posttraumatic sleep disturbance. Journal of Traumatic Stress: Official Publication of The International Society for Traumatic Stress Studies. 2004;17(3):257-68.
24. Victor SE, Klonsky ED. Validation of a brief version of the difficulties in emotion regulation scale (DERS-18) in five samples. Journal of psychopathology and Behavioral Assessment. 2016;38(4):582-9.
25. Ehring T, Svaldi J, Tuschen-Caffier B, Berking M. Validierung der Difficulties in Emotion Regulation Scale–deutsche Version (DERS-D). Unveröffentlichtes Manuskript, Universität Münster. 2013.
26. Gutzweiler R, In-Albon T. Überprüfung der Gütekriterien der deutschen Version der Difficulties in Emotion Regulation Scale in einer klinischen und einer Schülerstichprobe Jugendlicher. Zeitschrift für Klinische Psychologie und Psychotherapie. 2019;47:274-86.
27. Goodman R. Psychometric properties of the strengths and difficulties questionnaire. Journal of the American Academy of Child & Adolescent Psychiatry. 2001;40(11):1337-45.
28. Klasen H, Woerner W, Rothenberger A, Goodman R. Die deutsche Fassung des strengths and difficulties questionnaire (SDQ-Deu)-Übersicht und Bewertung erster Validierungs-und Normierungsbefunde. 2003.
29. Emam MM, Hilal MMA, Kazem AM, Alkharousi SJ. Psychometric properties of the Arabic self-report version of the strengths and difficulties questionnaire. Research in developmental disabilities. 2016;59:211-20.
30. Idzelis M, Ali AS, Gaaddasaar M, Bogoslaw LH, Ulstad K, Tanaka A. n. d. [Available from: https://www.sdqinfo.org/py/sdqinfo/b3.py?language=Somali.
31. Beck AT, Steer RA, Brown GK. Bdi-ii manual. 1996.
32. Hautzinger M, Keller F, Kühner C. Beck depressions-inventar (BDI-II): Harcourt Test Services; 2006.
33. Selmo P, Koch T, Brand J, Wagner B, Knaevelsrud C. Psychometric properties of the online Arabic versions of BDI-II, HSCL-25, and PDS. European Journal of Psychological Assessment. 2016;35:46-54.

For peer review only
