# Supplementary material for: START adolescents: study protocol of a randomised controlled trial to investigate the efficacy of a low-threshold group treatment programme in traumatised adolescent refugees
Source: BMJ Open. 2021 Dec 28;11(12):e057968. doi: 10.1136/bmjopen-2021-057968 (PMC8719222; doi:10.1136/bmjopen-2021-057968)
Supplement: Reviewer comments [file bmjopen-2021-057968.reviewer_comments.pdf]

PEER REVIEW HISTORY

BMJ Open publishes all reviews undertaken for accepted manuscripts. Reviewers are asked to complete a checklist review form (**http://bmjopen.bmj.com/site/about/resources/checklist.pdf**) and are provided with free text boxes to elaborate on their assessment. These free text comments are reproduced below.

ARTICLE DETAILS

**TITLE (PROVISIONAL)**	START Adolescents: Study protocol of a randomized controlled trial to investigate the efficacy of a low-threshold group treatment program in traumatized adolescent refugees
**AUTHORS**	Sobanski, Esther; Hammerle, Florian; Dixius, Andrea; Möhler, Eva; Koudela-Hamila, Susanne; Ebner-Priemer, Ulrich; Merz, Christian; In-albon, Tina; Pollitt, Brigitte; Christiansen, Hanna; Kolar, David; Ocker, Susanne; Fischer, Nicole; Burghaus, Ina; Huss, Michael

This article was not externally reviewed at BMJ Open. Protocols that have been independently assessed prior to submission to BMJ Open are usually fast-tracked to publication on the grounds that further substantial changes will not be possible. This independent assessment will usually be external, independent review for both a substantial grant award from a non-commercial or government funder and ethics approval.
